# Being a first generation university graduate, the impact on a career in science†

**DOI:** 10.1039/d5sc00205b

**Published:** 2025-04-30

**Authors:** Mariam Yacoub, Sarah Koops, Panagiota Axelithioti, Claudia Caltagirone, Emily R. Draper, Cally J. E. Haynes, Charlotte K. Hind, Marion Kieffer, Larissa K. S. von Krbek, Anna J. McConnell, Sarah J. Pike, Anna G. Slater, Jennifer R. Hiscock, Jennifer S. Leigh

**Affiliations:** a University of Kent, Canterbury Kent CT2 7NZ UK J.R.Hiscock@kent.ac.uk J.S.Leigh@kent.ac.uk; b Christian-Albrechts-Universität zu Kiel Kiel Germany; c University of Cagliari, Monserrato (CA) Italy; d University of Glasgow Glasgow UK; e University College London London UK; f UKHSA – Vaccine Development and Evaluation Centre Salisbury UK; g Independent researcher Zurich Switzerland; h Kekulé-Institut für Organische Chemie und Biochemie Bonn Germany; i University of Siegen Siegen Germany; j University of Birmingham Birmingham UK; k University of Liverpool Liverpool UK

## Abstract

Being in the first generation to access Higher Education (First Gen) is a barrier to academic success. First Gens face difficulties transitioning into, completing, and attaining competitive grades in undergraduate studies despite intervention strategies. Triangulating data across studies, we reveal the unique challenges resulting from being First Gen in science and show how these persist at every stage of a career in academia. We propose that invitation practices, *i.e.* behaviors that encourage, guide, and/or affirm others, need to be intentionally directed towards First Gens throughout their career journey to successfully support their retention and progression in science. As First Gens are overrepresented in other intersectionally marginalised groups, such actions will contribute to building a more inclusive and diverse scientific community.

## Abstract 2

We investigate the impact of being a first-generation student on academic success, not only on an individual's postgraduate studies but throughout a person's career, offering solutions to remove the barriers faced by these individuals.

## Graphical abstract

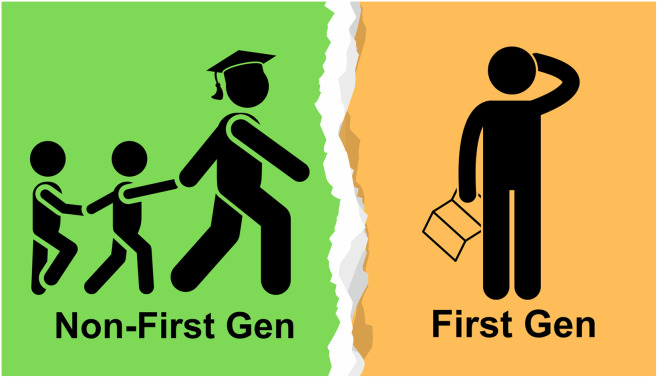

## Introduction

Being First Gen, that is, being in the first generation of a family to access Higher Education, is widely recognised as an obstacle to academic success.^1^ First Gen students are less likely to receive or pursue career guidance,^2,3^ more likely to struggle with the transition from school,^4^ have a higher risk of dropping out of university,^5^ and are less likely to attain competitive grades.^6^ These discrepancies are thought to be due to factors such as worries about finances^7^ and lack of family support.^8^ While there are different definitions of what constitutes First Gen,^9^† there is broad agreement that First Gens are disproportionately identified within underrepresented racial and/or ethnic groups and those from low-income backgrounds.^10,11^ In order to address the underrepresentation of diversity in academia, supporting these individuals has been the subject of many institutional programmes and initiatives across the globe.^12^ However, for the most part these initiatives focus on known challenges that impact all First Gens,^13^ such as supporting the transition into Higher Education,^14^ or the successful completion of a first degree.^15^ They do not address or differentiate between specific disciplinary contexts and rarely support First Gens as they navigate an academic career beyond obtaining their first degree. Building on the success of current global initiatives,^16–18^ we believe that achieving diversity in science demands more than just supporting an individual to obtain their first degree.

While there is a plethora of advice for would-be academics in general,^19,20^ few studies have been conducted that specifically focus on building a career in science.^21^ There is a notable gap in the literature when it comes to developing academic identity and a successful career in science, particularly for underrepresented groups. In addition, there are a range of disciplinary specific challenges that cannot be generalised. For example, thriving as a successful scientist almost always demands continuing study in the form of graduate degree(s).^21^ Postgraduate (PGR) study is known to be a period that is particularly challenging to navigate for individuals from groups historically underrepresented in academia.^22^ A scientist seeking to obtain an academic scientific position must normally obtain a PhD, followed by securing post-doctoral researcher position(s), fellowship(s), and/or successfully obtain grant funding^23,24^ in addition to maintaining a stellar record of research publications and collaborations to achieve success.^25^ There are well recognised barriers for women,^26^ Black,^27^ those with a minority racial and/or ethnic background,^28^ disabled,^29^ and LGBTQIA+^30^ scientists. It is no surprise that underrepresented groups report feeling isolated, suffer from imposter syndrome, and feel they do not belong.^28,31^ Given the overrepresentation of First Gens within these groups, individuals are likely to experience compounding intersectional barriers to achieving academic success.^32^ However, due to the conflation in many studies of being First Gen and being an individual with protected characteristics, the barriers specific to being First Gen for those wanting to establish or continuing an academic career in science are less understood.

Retention and progression of underrepresented groups in science is essential to achieve a diverse and inclusive scientific community.^33–35^ To support retention and progression of First Gens within science, it is essential to first understand the distinct ways in which First Gen scientists perceive and experience intersectional barriers to building their research career. From this unique vantage point, we can determine what is needed to build a more inclusive scientific community. Within the study presented, we seek to:

(1) Understand the unique ways in which First Gen scientists perceive and experience intersectional barriers to building their career and development;

(2) Identify features necessary for successful support interventions introducing the notion of ‘invitation’ as a practice for the continued support of First Gens throughout their academic careers.

Herein, we present findings from a semi-systematic review of literature on First Gen chemists and scientists together with the first data on a mixed cohort of researchers across multiple career stages involving over 300 international scientists who predominantly identified as supramolecular chemists. Supramolecular chemistry is a somewhat unusual field within chemistry as it encompasses physical, inorganic, and organic chemistry.^36^ In addition, supramolecular chemistry is adjacent to other physical science and bioscience disciplines,^37–39^ and collaboration and movement between these disciplines is common.^40,41^ As such, supramolecular chemistry can be viewed as a microcosm of scientific disciplines and representative of some of the more challenging conditions for retention and progression of underrepresented groups. Our sample had an overrepresentation of participants who identified as having one or more protected characteristic(s) (36%), and an overrepresentation of women across all career stages. Women are generally considered to be underrepresented in chemistry and science and this underrepresentation is known to increase with career stage.^42^

## Experimental

Building an inclusive scientific community necessitates novel approaches that instigate cultural change.^43^ This paper triangulates findings from three studies: a semi-systematic literature review; a community-led online survey; and community-based in-person creative and reflective workshops. Embodied Inquiry was used as the conceptual framework and theoretical approach for this research.^44^ See Section S2† for an overview of this approach and details of the combination of methods used within each study. The theoretical framework for this research is Embodied Inquiry,^44^ which is a theoretical and methodological approach to research design, data generation, capture and analysis. When used as a theoretical framework, Embodied Inquiry enables rigorous research through gathering robust data for a mixed-methods analysis whilst encouraging reflection, emotional engagement, and connection so that research is with rather than on participants. This study builds on previous work developed to support underrepresented groups in supramolecular chemistry,^43,45,46^ and biology based doctoral students.^47^ The focus on the lived and embodied experiences of First Gens arose from community led discussions on the barriers and obstacles for specific groups identified within a collaborative autoethnography.^43^ Here we use a selection of creative and embodied methods with the community to gather multi-layered data, facilitate meaningful participation, build trust, allow people to share and process experiences and the emotional impacts of barriers, and reflect on how they might be overcome or ameliorated. Throughout all the studies the data were analysed iteratively and reflexively with themes identified as a result of the data analysis. This enabled the community to raise the themes which impacted on them, as opposed to the research leads introducing predetermined themes.

### Study 1: semi-systematic literature review

In order to obtain a broad overview of the current state of research on First Gen scientists and identify areas for further exploration, we conducted a semi-systematic literature review. This combined a systematic approach to surveying and selecting literature^48^ with a written discourse^49^ to allow for a diversity of ways of understanding or “plurality of knowing”.^50^ The aim was to identify literature sources concerned with First Gens and chemistry, though our search was widened to include more general literature encompassing First Gens, wider science disciplines, EDI (equity, diversity and inclusion), chemistry, outreach, and widening participation. This broad search led to identification of 156 sources. After applying inclusion and exclusion criteria 10 sources were excluded, 136 were categorised as background literature which was still analysed and coded, and 10 sources were selected for inclusion (Table S2†). Of these published works, half measured the success and/or impact of various interventions for undergraduate (UG) First Gen chemists. Five considered transitions into Chemistry UG programmes and/or introductory courses to UG Chemistry for First Gens.^15–17,51,52^ Two examined the experiences of First Gen chemistry UGs.^53,54^ One included doctoral Chemistry students in addition to UGs and measured levels of satisfaction with financial aid and peer support for minority and First Gen students.^55^ One source focused on high school students and beliefs around maths and science.^56^ The final source explored the impact of a professional development programme for faculty on inclusion of First Gens.^57^ Our review confirmed significant gaps in the literature relating to:

(1) First Gen experiences of building a career in scientific research;

(2) Identification and inclusion of challenges specific to First Gens in science as part of intersectional inclusion and support initiatives.

In contrast, the key areas of interest identified through inductive and reflexive thematic analysis of the background literature included general barriers experienced by First Gens, a lack of capital or knowledge, and a lack of support and/or access to resources (Tables S3 and S4†). A survey, Study 2, was subsequently designed to explore how these key areas of interest were experienced by the scientific community.

### Study 2: community-led online survey

**Fig. 1:**
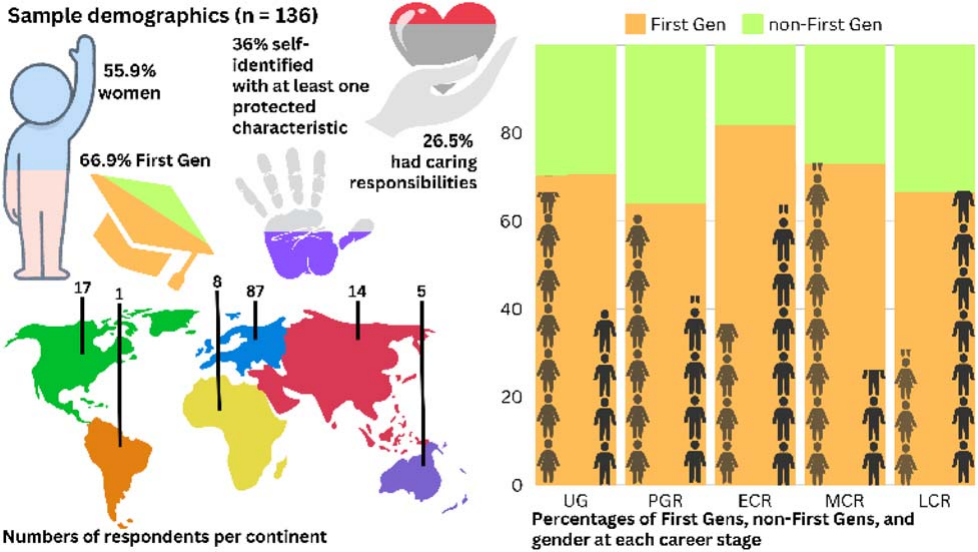
Survey sample demographics (*n* = 136) showing numbers of respondents across continents, proportion of women, First Gens, people identifying with at least one protected characteristic (*e.g.* race, ethnicity, disability, sexuality), and caring responsibilities for a child and/or others. UG = Undergraduate, PGR = Postgraduate Researcher, ECR = Early Career Researcher, MCR = Mid-Career Researcher, LCR = Late Career Researcher.

A survey containing questions informed by Study 1 was completed online by 136 international participants from the scientific community. The questions were designed to elicit mixed data to enable both qualitative and quantitative analysis (Table S4†). The structure was modelled on previous successful community-led surveys from the International Women in Supramolecular Chemistry (WISC) network.^44,46^ It was open to participants of all genders and career stages, whether they identified as First Gen or not. The data were cleaned then analysed in three stages; (i) categorisation of answers to demographic questions before simple descriptive statistical analysis, (ii) reflexive thematic analysis of long-text answers, and (iii) categorisation of coded long-text answers for regression analysis.^58^ This mixed-methods analysis allowed us to identify broad patterns and then ascertain whether these patterns were statistically significant. The participants were broad in terms of geographic spread, career stage, gender, and caring responsibilities (Fig. 1). The survey questions were designed to better understand First Gen experiences, and whilst our sample was skewed towards First Gens, 33% of participants identified as non-First Gen, enabling comparison between these two groups. The majority of respondents were born and/or raised in Europe. Participants originated from six continents, although now only resided in five. Of the total number of survey participants; 45% were PGR researchers completing Masters or PhD studies, 19% were mid-career stage (MCR), 13% UG, 9% were classified as late-career stage (LCR), and 8% early-career (ECR). Most stated they had no caring responsibilities, though 18% reported caring for a child or children, 5% caring for an elderly person, and 2% a mix of caring responsibilities (see Section S3.2† for full descriptive statistics and demographics).

### Study 3: reflective workshops

Finally, in-person creative research approaches were used in three workshops held as part of disciplinary conference events. The workshops were designed to facilitate an environment where participants were encouraged to reflect, process, and share their lived experiences, including the experience of being First Gen. Although students of the physical sciences are not routinely taught to reflect as part of the curriculum, when given the opportunity this approach has proved effective at building a sense of community whilst gathering robust data.^59^ Within these workshops 108 pieces of hard-copy data and two online notice boards (individual and group responses) were collated and analysed using reflexive thematic analysis. When reporting on these data where a participant clearly stated their demographics, these have been included, otherwise not.

## Results and discussion

Study 1 was used to guide the questions asked in Study 2. Studies 2 and 3 were used to generate data enabling insight into the experiences of First Gen scientists and identify where those experiences were specific to First Gens as well as where they connected and overlapped with experiences of other underrepresented groups, and where they were common to many individuals in science. As previously reported,^60^ some Study 2 participants commented that being First Gen was not always an identity they had considered:

“*I didn't really think about this in the beginning. Only when I started my PhD*.” First Gen, man, LCR, white.

Others owned their First Gen identity completely:

“*It means a lot to me and is a thing of pride/joy*” First Gen, woman, PGR, black.

**Fig. 2:**
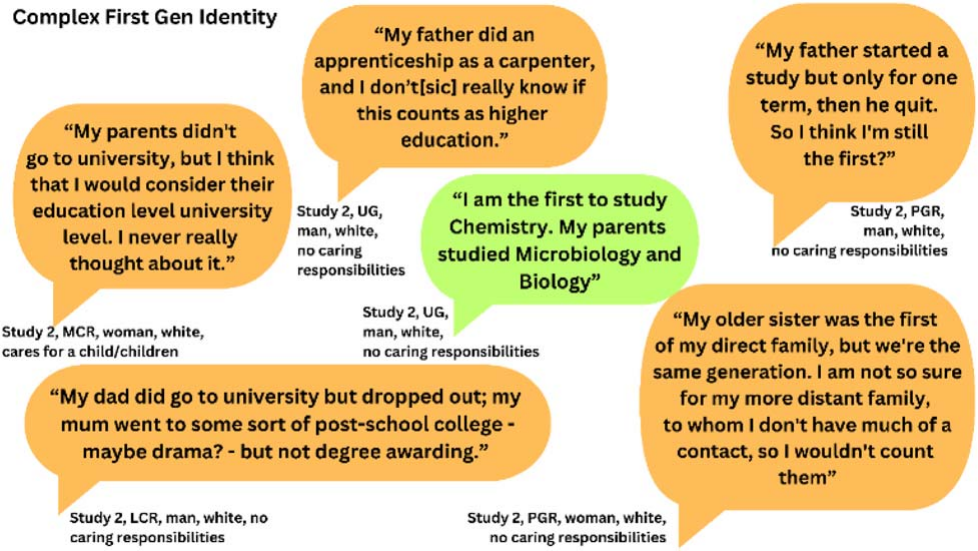
Quotes from individuals that illustrate the complexities of First Gen identity. Quotes from individuals categorised as First Gen are shown in orange, non-First Gen are shown in green. The study that generated these data are also provided alongside details of the individual that provided the quote.

For many, the idea of being First Gen was complicated, or they were conflicted about their status (Fig. 2). When categorising participants in our analysis, five were labelled First Gen as their parents had not successfully completed a course of Higher Education study. The sixth, who said he was the first to study chemistry, was not.

Having identified the complex nature of First Gen identity, we report and discuss our findings under three broad themes:

(1) Capital;

(2) Barriers, with sub-themes,

(a) Financial burden,

(b) Isolation,

(c) Imposter syndrome;

(3) Access, with sub-themes,

(a) Invitation,

(b) Community.

### Capital

This theme directly relates our findings to the existing literature around First Gen experiences in Higher Education. A well-established barrier for First Gens, as reported by Gangitano,^52^ Uche,^15^ and corroborated from the data here, is a lack of social and cultural capital.^61^ Social and cultural capital refers to the understanding and knowledge of norms and systems in society that are afforded to someone through their networks. It “*is acquired over time*...*It can enable an individual to navigate a field, knowing the ‘rules of the game*”.^62^ For most people our family form our primary network and source of building capital prior to adulthood. Families therefore play a huge role when it comes to choosing a career or life path and understanding how to navigate it. Beyond the family, an individual's peers and colleagues are likely the next port of call to build social and cultural capital. Please see ESI materials Section S1† for more context on Bourdieu's conceptualisations of social and cultural capital.

Study 2 data showed First Gens are 10% more likely than non-First Gens to say they had no support at all– from family or elsewhere. Unsurprisingly, First Gens were also 26% less likely to say they had family support than non-First Gens (Table S8c†). This lack of family support was not found to be significantly compounded by gender, having a protected characteristic, caring responsibilities or moving country (Table S8d†). One respondent said:

“*I have felt slightly disadvantaged or like I am a step behind everyone else as I can't really go to my parents for advice or help as they don't fully understand the higher education system or how it works etc. So I've had to spend a lot of time finding out this information myself, which obviously puts me behind other people who have people in their life that have been through the university experience*.” Study 2, First Gen, PGR, woman, white, no caring responsibilities.

While others used family networks to receive support from university faculty:

“*I went to talk to a professor at the chemistry Dep because my dad knew her personally*” Study 2, non-First Gen, MCR, woman, white, cares for a child/children.

“*I remember my PI bringing his son to work along with us in the laboratory so that he could have his first experiences in the lab*.” Study 3 participant.

Non-First Gens described how family networks supported them emotionally and financially:

“*[In response to sources of support] My bank account my parents did for my studies*” Study 2, non-First Gen, UG, man, white, no caring responsibilities.

*“My parents encouraged me to apply to University”* Study 2, non-First Gen, PGR, man, white, cares for parents.

The advantages of social and cultural capital can manifest in many areas of academic life such as being pre-exposed to the academic environment or having confidence navigating the systems and social networks necessary for career advancement. A deficit of social and cultural capital can lead to intense feelings that last throughout even a successful scientific career:

“*My whole career has been a surprise- I have landed on my feet so far, but not because I knew what I was aiming for!*” Study 2, First Gen, LCR, woman, white, no caring responsibilities.

Being First Gen often intersects with lower socioeconomic backgrounds, leading to a demonstrable discomfort and lack of capital around ‘middle class’ social interactions as well as research specific activities:

“*The whole thing, start to finish. Trying to understand influence networks, the old boys club, how funding works, social interaction in the middle class, etc.*” Study 2, First Gen, ECR, man, white, no caring responsibilities.

As seen in Study 1, current research on or with First Gens is skewed towards UG or PGR cohorts and their experiences of transitioning into Higher Education. Here, we show that the landscape, networks, and environment in scientific research can be equally difficult to navigate for First Gens throughout their career. A full Professor shared:

“*I am still learning the nuanced ways the career of others can be advanced by a strong pedigree and a good mentor (who advocates and promotes their former co-workers). The majority of careers in the field spring forth from a relatively small number of (big) groups, and having never been part of this can feel quite isolating sometimes. If the field is a river, those main currents are quite distant, and being a first generation researcher is akin to being a small pool on the bank. One spends an inordinate amount of time clearing boulders and debris in an attempt to become an acknowledged part of the main flow*.” Study 2, First Gen, LCR, man, white, cares for a child/children.

Overall, the data captured and generated as a result of our embodied inquiry supports and extends understanding of how a lack of social and cultural capital can be linked to barriers around networking, guidance and expectations, accessing resources, and feeling isolated or alone in Higher Education and research environments.

### Barriers

**Fig. 3:**
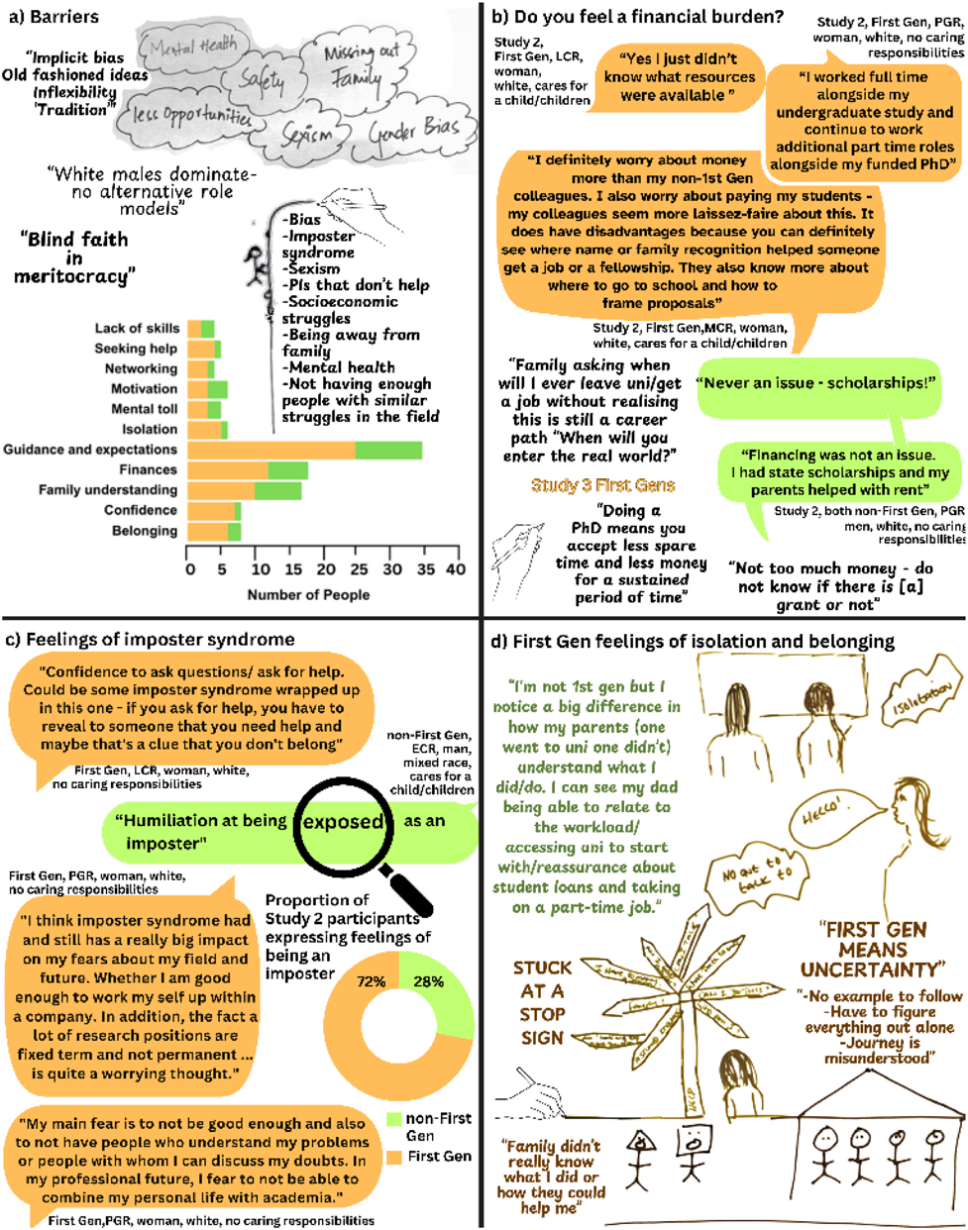
Quotes and drawings from participants of Studies 2 and 3. Data from First Gens are shown in orange, Non-First Gens are shown in green. (a) Illustrates key barriers to Higher Education and/or research careers shared by participants of Studies 2 and 3. (b) Quotes from Studies 2 and 3 relating to feelings of financial burden. (c) Quotes from Study 2 relating to participants' imposter feelings. (d) First Gen reflections on belonging and isolation from Study 3.

All the participants in Studies 2 and 3 were asked about the barriers and opportunities they experienced in Higher Education and through their academic careers. Examples of their responses are provided in Fig. 3a. Many of these, such as “*bias*”, “*sexism*” and “*unhelpful P.Is* [Principal Investigators]” suggest that underrepresented groups in particular feel the impact from a lack of role models, supportive networks, and low levels of diversity within the scientific working environment. First Gen participants also identified barriers specific to being First Gen including financial burden, isolation, and a lack of guidance resulting in not knowing what to expect, reduced feelings of confidence and belonging, and an inability to network.

When asked explicitly about the barriers First Gens might face, two-thirds of non-First Gen participants either did not provide an answer or indicated that they did not feel there were significant challenges for this group. Of the non-First Gens who did understand there were particular challenges for First Gens, 86% were postgraduate or undergraduate students. This indicates awareness of the unique experiences of First Gens amongst people early in their careers in Higher Education and science:

“*I believe they had to work against more[,] most likely. Rather than being assumed to go into a bachelors or graduate degree, they likely had to motivate themselves to continue through it despite family potentially feeling like it is unnecessary or a waste of time*.” Study 2, non-First Gen, PGR, woman, white, cares for a child/children.

However, we believe that unless they are themselves First Gen, many senior scientists with the authority and resources to support, guide, or mentor early career researchers are likely to remain largely unaware of barriers for this group.

#### Financial burden

First Gens are reported to feel an increased financial burden compared to non-First Gen peers or other underrepresented groups.^7,15^ Our data corroborates this, and uniquely demonstrates the long-lasting impact that financial burden places on First Gens throughout a scientific career, not only during transition into or completion of UG study. In Study 2, analysis showed First Gens were 28% more likely to feel a financial burden compared to their non-First Gen counterparts, with double the proportion of First Gens mentioning financial burdens as non-First Gens. This correlation was not confounded by gender, protected characteristics, caring responsibilities or by the participant moving country. In fact, the impact of being First Gen increased in the presence of these variables, staying significant and rising to 28.6% (Tables S8a and S8b†). First Gens are often international students or first-generation immigrants,^9^ resulting in intersecting financial barriers. For example, one participant described coming from a middle class Pakistani family who could not offer financial support whilst she studied in the UK. The comparison between groups was striking. Non-First Gens commonly referred to sources of additional family support and accumulating various types of financial aid. They demonstrated knowledge of systems and scholarships as much as familial support. Conversely, very few First Gens included within Studies 2 and 3 mentioned sources of financial support. Whilst efforts have been made in the UK and other countries to set up scholarships, grants, and bursaries to ‘widen participation’ in Higher Education and support underrepresented groups,^16,17,52,53^ the individuals requiring these resources seemed largely unaware of them. The majority of our First Gens described working one or multiple jobs whilst studying to finance education and living costs. As indicated by the literature reviewed in Study 1, our data show First Gens experience a greater financial burden than non-First Gens. We hypothesise this is in part due to a lack of knowledge and/or guidance on how to access resources.

#### Isolation and imposter syndrome

The concepts of imposter syndrome, self-confidence, and belonging are closely related and often used interchangeably.^10–12^ Imposter syndrome is defined as a behavioural health condition resulting in depressive tendencies, harsh self-criticism and social anxiety. Sufferers attribute success to luck, timing, or even charm rather than their own competence.^13^ Many of these behaviours, such as self-criticism, are associated with low self-confidence. Discourses on student experiences of belonging often refer to connectedness and socialisation leading to better education and life outcomes.^63,64^ Essentially, a sense of belonging is the opposite to isolation. Feelings of isolation result in low self-confidence and other mental health challenges.^65^ Imposter syndrome, self-confidence, and belonging have been shown to have varying effects on student experience.^66–68^ However, they are neither well explored in relation to First Gen status, nor well investigated in the research community beyond UG and PGR study. In order to shed light on First Gen experiences we included questions within Study 2 relating to fears, apprehensions, and general experiences of studying and researching. Where participants expressed fears of being an outsider, inadequacy and/or fraudulence, the data were coded to ‘imposter feelings’. Twenty-nine Study 2 participants described experiences categorised as imposter feelings, for example:

*“I was worried when I got to college I would be out of my league.”* Study 2, First Gen, MCR, woman, white, cares for a child/children.

We also found a significant correlation between feelings of isolation and feelings of imposter syndrome, though imposter syndrome was more widespread across the sample. Whilst the majority of expressions of imposter syndrome (72%) were from First Gens, non-First Gens were not exempt from feeling as though they did not belong (see Fig. 3c). In all, 23% of First Gens spoke about feeling like an imposter, compared to 2% of non-First Gens. Participants in both Studies 2 and 3 used terms such as “*exposed*’ and “*not good enough*” to describe academic life. Workshop participants from Study 3 reflected on their experiences (see Fig. 3d). One participant wrote “*you had to be part of the “club” in this group (to get a chance to speak at meetings for example)*”. The use of the word ‘club’ emphasises the experience of being an outsider without an invitation to join the exclusive inner circle who are afforded additional respect or privilege. They continued: “*Women or members of other marginalised groups would have a harder time breaking in.*” The phrase ‘breaking in’ implies a wall, barrier, or separation that must be overcome if individuals are to participate fully, even within a research group. Similar sentiments are seen in another participants' drawing showing individuals (un)intentionally denied access to an institution or identity through a building housing figures with the same shape head with others excluded. First Gen participants shared a unique sense of isolation resulting from a lack of family support, understanding, or other supportive networks:

“*Understand that many of us do not have networks of friends and supporters in what we do. Many of us also carry heavy financial burdens into careers if from poorer backgrounds, so self-funding internships, conferences and events is often impossible*.” Study 2, First Gen, ECR, man, white, no caring responsibilities.

“*There's no one in your immediate family whom you can look up to*” Study 3.

“*Have to figure out everything alone*” Study 3.

It is well documented that supportive familial and/or social networks and interactions are vital for well-being.^69,70^ Given the level of isolation First Gen participants described experiencing, it is likely they will encounter significant negative impacts on their wellbeing and mental health throughout their career.

### Access

**Fig. 4:**
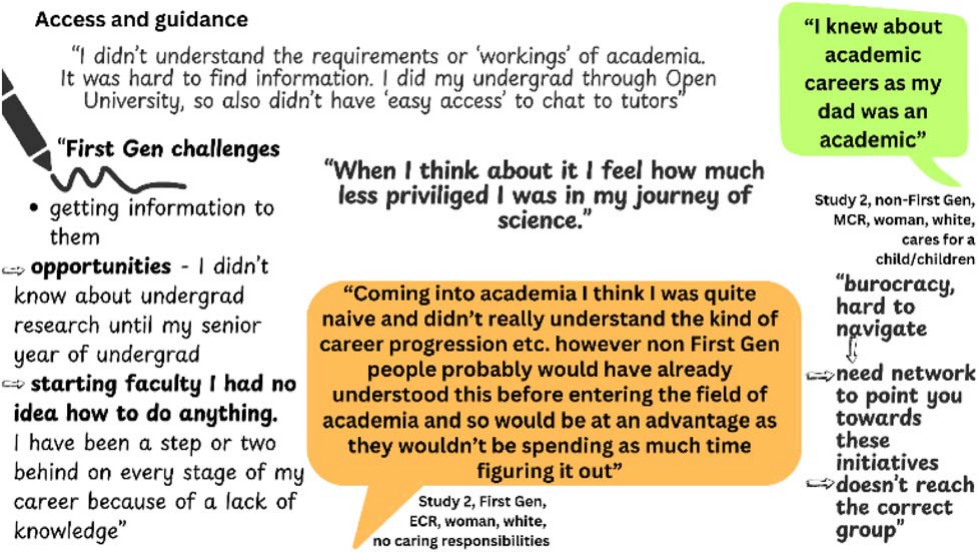
Reflections from First Gens (Study 3) and quotes from Study 2 participants on access and guidance. Quotes provided by First Gens are shown in orange, those from non-First Gens are shown in green.

Those from underprivileged backgrounds have less access to good quality resources and guidance, particularly around careers advice.^2^ A lack of guidance or expectations was the most frequently mentioned challenge in Study 2 for both First Gens and non-First Gens. We defined ‘guidance’ to include signposting towards resources, options, and opportunities. Expectations were defined as being exposed to or told directly what to expect and/or how to navigate upcoming experiences specifically related to academic progression. As shown in Fig. 4, participants across Studies 2 and 3 described challenges around career paths and reflected on the scarcity of resources and guidance.

Interestingly, when it came to accessing resources, we found men were 12% less likely to use online resources than women and other minoritized genders. This could be connected to the additional barriers and increased feelings of isolation experienced by women in science: Women are less likely to be credited for their contributions in science.^71^ They are less likely to be confident even when they are competent,^72^ and with fewer role models to look up to and be guided by, it makes sense they would be more likely to source resources online than risk being seen as incompetent or needing help. However interestingly, we found no relationship between being First Gen and accessing resources online. Being First Gen is a hidden identity, and unless explicitly disclosed, it would not necessarily be obvious from the outside. In Study 2, First Gens frequently expressed experiencing inadequate guidance and lack of expectations as they described persisting in and completing study, or progression through an academic career. They used phrases including “*never encouraged*”, “*clueless*” and “*I hadn't considered continuing down the academic route*”.

As seen in the sub-theme ‘financial burdens’, a lack of access to information and opportunities compounds other barriers. Our analysis has led us to believe that the key to understanding and addressing disparities between the experiences of First Gens and their non-First Gen counterparts is recognizing that First Gens experience a deficiency of invitation behaviors compared to their non-First Gen counterparts.

#### Invitation

This sub-theme was identified through analysis of data from Studies 2 and 3. Invitation in this context can be defined as a direct message of encouragement, support, guidance and/or validation extended from someone within the academic community to someone either outside of it or less experienced than themselves. Invitation practices or behaviours are deliberate actions that extend these messages either directly or indirectly. Many Study 2 non-First Gens shared how an academic figure had been influential to them:

*“My mentor supported me in pursuing a PhD”* Study 2, non-First Gen, MCR, woman, white, no caring responsibilities.

“*University careers advice centre and members of the university lab I worked in after my degree (because I did a vacation placement in a uni lab)*” Study 2, non-First Gen, LCR, woman, white, cares for a child/children.

**Fig. 5:**
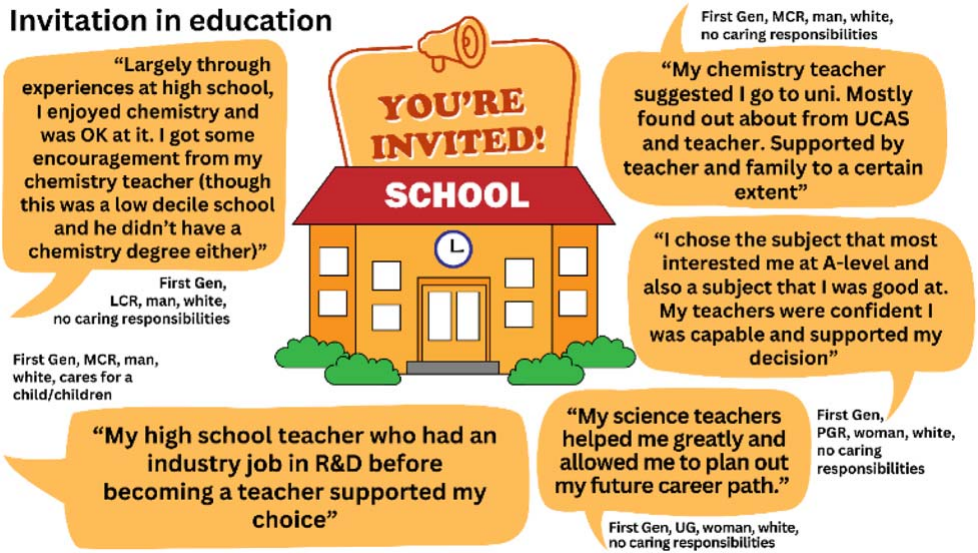
First Gen participant responses from Study 2 demonstrating the impact of invitation from teachers and school staff. “UCAS” mentioned by one participant is the University and College Admissions Service, operating in the United Kingdom.

In other words, these individuals were ‘invited’ onto the pathway of a career in scientific research, which emphasises the positive influence of invitations and interactions with faculty. This also highlights how much more common it is for non-First Gens to access the kind of positive relationship with a faculty member that will facilitate an invitation both into and then within Higher Education. Being ‘invited’ when a student is in secondary education is incredibly influential.^73^ It is well established that invitation behaviours, although not defined as such, are key for encouraging underrepresented groups to progress into Higher Education.^74–76^ Teachers are recognised as significant players in raising the science capital of their students.^77^ Invitation makes people aware of their potential, affirms their capabilities and aspirations, and directs them towards opportunities and resources to guide their journey. Our data corroborate these findings. Many First Gens described invitation behaviours from teachers and education practitioners in their secondary education that positively influenced their choices, see Fig. 5.

In addition, our data demonstrates how First Gens were successfully supported transitioning into science and Higher Education through invitation behaviours that allowed them to build self-confidence and develop capital. However, when reflecting past this transition point, there was little mention of invitation behaviours. We propose intentionally implemented invitation behaviours within Higher Education practiced throughout the academic career is the key to providing targeted support for First Gens and other underrepresented groups in science. Intentional invitation behaviours will increase the chances of First Gens and other underrepresented groups to initially pursue and later thrive in a career in scientific research. Our conceptualisation of ‘invitation’ requires the inviter to be embedded in academia. However an ‘invitation’ can take many forms. It might be asking an UG student if they have considered graduate study, and/or pointing them towards scholarship opportunities. It could be a group leader ‘inviting’ a post-doctoral fellow to consider applying to a fellowship or lectureship, or a post-doctoral fellow complimenting the research abilities of a PhD student and encouraging them to continue, alerting them to opportunities to do so. For anyone wanting to practice invitation behaviours, thoughtful consideration and reflection on unconscious bias is vital to ensure particular demographics or groups are neither prioritised nor neglected. These practices must reach beyond immediate circles of colleagues and be intentionally targeted towards historically underrepresented groups to help create a more diverse and inclusive scientific community.

#### Community

Students and ECRs are often advised to find a community for support, particularly if they are from a historically underrepresented group.^69,70,78,79^ For example, finding a trusted network of peers and mentors is vital for the retention and progression of women in science.^80–83^ The importance of a community or network as a means of sharing lived experiences, disciplinary specific support, and signposting guidance was clear for the participants of Studies 2 and 3. They used networks to find and connect with peers and mentors. This was even more important for those without family support such as First Gens:

*“Use your network, your chosen family for help if you don't have it in your family*.*”* Study 2, First Gen, ECR, woman, white, cares for a child/children.

“*Networking is your friend and will only help you in your future endeavours. The more people you interact with in the field the more you can develop working relationships for example working with others on a project or getting ideas and thoughts from them*” Study 2, non-First Gen, PGR, woman, white, no caring responsibilities.

**Fig. 6:**
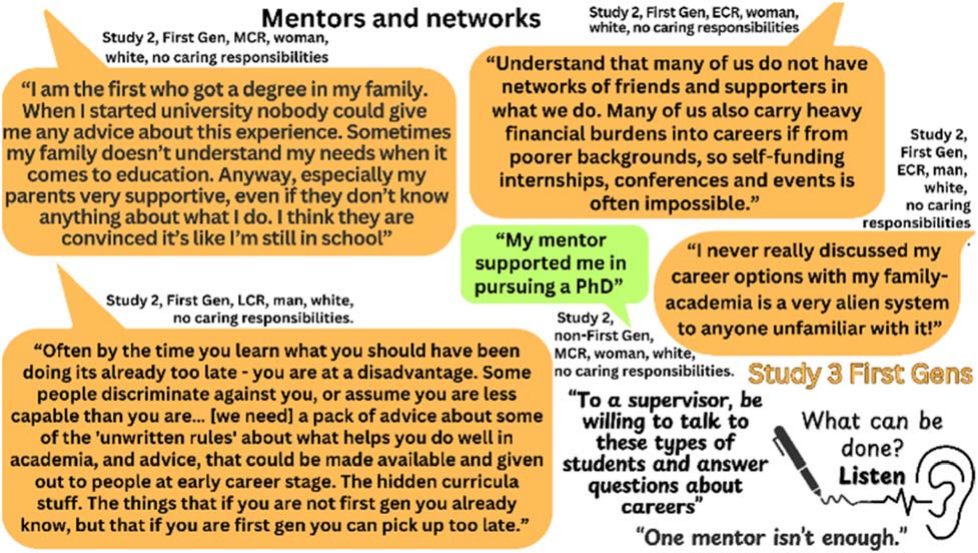
Quotes from participants reflecting on their experiences of having mentors and guidance. Quotes provided by First Gens are shown in orange, those provided by non-First Gens are shown in green.

Guidance is essential to navigate career progression in scientific research, with the positive impact of mentors for underrepresented groups^26^ well recognized (see Fig. 6):

“*My grad student that I worked with and my undergraduate research mentors helped me with choosing a grad school. My dissertation mentors helped me with a postdoc and career choice. My parents and now spouse always supported me. My PhD advisor sometimes was like* “ok if that's what you really want to do - I'll help.” Study 2, First Gen, MCR, woman, white, no caring responsibilities.

“*I needed a mentor that would explain their career journey, offer other career paths and introduce me to other people they may know on other career paths for me to meet and question*.” Study 2, First Gen, ECR, woman, white, no caring responsibilities.

*“Get mentors---several of them and listen!”* Study 2, First Gen, MCR, woman, Black, no caring responsibilities.

Networks and mentors are particularly key for First Gens who lack social and cultural capital and are unable to access information through family networks. Study 2 participants advised other First Gens to establish their own networks if they did not already have them:

“*Contact people already in the system, postgraduate students, academics, support staff. People are typically very happy to help or provide advice. If you don't feel confident approaching such people, write them an email*.” Study 2, First Gen, ECR, man, white, no caring responsibilities.

Communities and networks provided opportunities to share lived experiences with people who had been through something similar. They provided safer spaces to connect and helped ameliorate feelings of isolation and loneliness:

“*A safe space to ask questions, guidance on the different paths available within academia- and also information about alternative career paths*.” Study 2, First Gen, LCR, woman, white, no caring responsibilities.

“*Understand that it will be extremely overwhelming at first, but it really does get better with time. You'll get used to how the system works and what's expected of you over time. Having a routine and keeping a list of all of your responsibilities and expectations definitely helps*” Study 2, First Gen, PGR, man, white, no caring responsibilities.

*“You are not alone. You are not the only one”* Study 2, First Gen, LCR, man, white, no caring responsibilities.

**Fig. 7:**
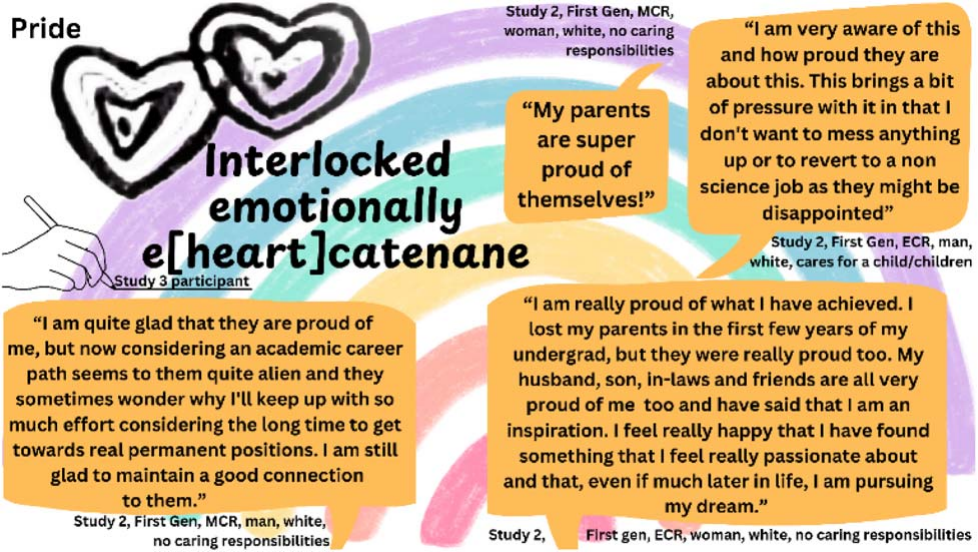
Quotes and an illustration highlighting First Gen reflections and expressions of pride, including a ‘catenane’ shaped as two interlocking hearts.

Not all community has to be academic. Despite many First Gens reporting feeling alienated and alone because their families did not understand the challenges they faced, many others spoke of strong support systems and pride from their families, akin to pride associated with other marginalisations. Whilst a lack of capital meant families were unable to share information or set expectations about Higher Education, First Gens gained emotional support and encouragement as they persevered through studies and careers (Fig. 7). One Study 3 participant drew an image of interlocking hearts, labelling it “*interlocked emotionally e[heart] catenane*”. A catenane is a supramolecular molecule consisting of two or more interlocking macrocycles– in this case represented by the two interlocking hearts.

Communities and networks are also vital for raising awareness about the hidden needs of underrepresented groups. This research stems from and was supported by the international Women in Supramolecular Chemistry (WISC) network. WISC itself grew from peer support established by a small group of women who felt isolated, to become an international community with its own mentoring programme and community events.^44^ Within Study 2, participants were asked how initiatives (specifically WISC) could support First Gens. Many responses mentioned the positive impact of raising awareness:

“*Raising awareness that academic careers are a greater challenge for 1st generation scientists is already a great start and I very much appreciate it, since so far I think barely any colleagues have considered this fact. This might partially be related that most of them actually came from families with at least one member having academic background. I hope in future this initiative will lead to additional support in terms of funding for scientists in later career steps or at least provide early guidance to 1st gen students in terms of what they are going to face and how they should be prepared for this*.” Study 2, First Gen, MCR, man, white no caring responsibilities.

“*The more conversations we have and the more people involved the better*” Study 3, online noticeboard.

“*WISC could organize seminars to mentor specifically 1st Gen supramolecular chemists or guest edit a special issue!*” Study 2, non-First Gen, MCR, woman, cares for a child/children.

Raising awareness is necessary while people still remain unaware of the specific challenges facing groups such as First Gens, for example the isolation due to a lack of family support and the obstacles to accessing guidance and resources such as mentors:

“*I do not think there are disadvantages as long as they (First Gens) have supporting mentors and families*” Study 2, non-First Gen, MCR, woman, white, cares for a child/children.

As previously discussed, positive influences and guidance from mentors and communities are known to be of particular benefit to First Gens and other underrepresented groups.^26,44^ However, our data indicate a clear disparity between the experiences of First Gens and their non-First Gen counterparts when it comes to the ability of First Gens to identify, access, and use support from networks, communities and mentors to benefit their career.

## Conclusions

Barriers to retention and progression such as discrimination, bias, and imposter syndrome impact many groups historically underrepresented in science. First Gens face unique and intersecting challenges. The impact of these challenges lasting throughout career journeys has been clearly demonstrated by the data capturing the embodied and lived experiences of First Gen scientists at different career stages. In addition to being more likely to come from an underrepresented group, First Gens lack social and cultural capital, family support, are less likely to access guidance, resources or make informal networks, and feel isolation and financial burden more than non-First Gens. Being First Gen is invisible and complex, complicating effective signposting, identification, and eligibility for targeted initiatives. Reduced access to career guidance together with a lack of capital suggests First Gens are less likely to feel part of communities, networks or pull on those they do have to advance their career than non-First Gens. We have evidenced that even First Gens who are well established and senior within their field remain aware of the persistent and negative impact of barriers to their progression: First Gens remain *“a step or two behind”* at every stage.

The overrepresentation of First Gens in other underrepresented groups means effective, targeted, and intersectional interventions from individuals at established events such as conferences could help build a more inclusive scientific community. Intervention is needed to address First Gens' lack of access to the guidance, resources, supportive networks and mentors that facilitate feelings of belonging, career satisfaction, retention, and progression. Given the impact of invitation behaviours for initiating First Gens into Higher Education, practicing simple, affirmative, and intentional invitation behaviours towards First Gens and other underrepresented groups should become commonplace for all people working within scientific research. Feeling ‘invited’ by mentors and to networks throughout an academic career offers the best chance for progression and success. However, invitations must be personal or come from a supportive community, not be a ‘tick box’ approach. Successful mentoring requires a mentor invested in their mentee's success. Successful networks facilitate authentic connections and a sense of belonging to a community. Both ameliorate the feelings of isolation and imposter syndrome endemic in First Gens and other underrepresented groups.

In order to initiate the cultural changes that are needed to diversify chemistry and science, action needs to come from professional bodies and Higher Education institutions as well as individuals. As a first step, raising awareness and recognising the impact being First Gen can have on progression and careers is vital. Acknowledging the existence of different types of barriers immediately extends discourses around diversity and inclusion to include groups who might not conventionally be considered to be marginalised (such as white men from low socio-economic backgrounds). Similarly, widening access to initiatives that have been implemented to support specific groups will embrace intersectional identities. Secondly, it is important that institutions and professional societies practice scaled-up invitation practices. They can do this by taking responsibility for inviting and welcoming individuals into different spaces without assuming that everyone knows what to do or how to behave. Clearly stating what is expected without a sub-text or ‘hidden handbook’ known only to those familiar with academia would help First Gens and other underrepresented groups make the most of academic conferences, talks, question and answer sessions, open days, or social events. Institutions and professional societies can easily facilitate the expansion of people's existing academic networks and relevant knowledge and therefore play a role in raising social and cultural capital for students and faculty at all stages of their career. Invitation practices are a simple yet potentially transformational means to facilitate inclusion so everyone has the opportunity to achieve success within the chemical sciences.

## Author contributions

MY: analysis, validation, writing – original draft, review & editing; SK, PA: investigation, analysis; CC, ERD, CJEH, CKH, MK, LvK, SP: conceptualization, investigation, writing – review & editing; AJM: funding acquisition, conceptualization, investigation, writing – review & editing; JRH, JSL: conceptualization, investigation, data collection, analysis, validation, funding acquisition, project administration, supervision, writing – original draft, review & editing.

## Conflicts of interest

There are no conflicts to declare.

## Supplementary Material

SC-016-D5SC00205B-s001

## Data Availability

The data supporting this article have been included as part of the ESI.†
