# Supplementary material for: Being a first generation university graduate, the impact on a career in science
Source: Chem Sci. 2025 Apr 30;16(23):10386–96. doi: 10.1039/d5sc00205b (PMC12062832; doi:10.1039/d5sc00205b)
Supplement: SC-016-D5SC00205B-s001 [file SC-016-D5SC00205B-s001.pdf]

Supplementary Information (SI) for Chemical Science.
This journal is © The Royal Society of Chemistry 2025

Being a first generation university graduate, the impact on a career in science: Supplementary Materials

Mariam Yacoub, Sarah Koops, Panagiota Axelithioti, Claudia Caltagirone, Emily R. Draper, Cally J. E. Haynes, Charlotte K. Hind, Marion Kieffer, Larissa K. S. von Krbek, Anna J. McConnell, Sarah Pike, Anna G. Slater, Jennifer R. Hiscock and Jennifer S. Leigh.

Corresponding authors: J.R.Hiscock@kent.ac.uk and J.S.Leigh@kent.ac.uk

Contents

Section S1 First Gen, ‘Underrepresented Minorities’, and belonging 3
Section S2 Theoretical and methodological approach 5
Section S3 Study 1 6
3.1 Semi-systematic literature review *6*
3.2 Analysis *6*
Section S4 Study 2 8
4.1 Online survey *8*
4.2 Participant demographics and descriptive statistics *8*
4.3 Analysis *9*
4.3.1 Qualitative analysis 9
4.3.2 Quantitative analysis 11
4.3.3 Regression Analyses 12
Section S5 Study 3 14
5.1 Reflective workshops *14*
5.1.1 1st International Supramolecular Summer School (ISSS) 14
5.1.2 International Symposia on Macrocyclic and Supramolecular Chemistry (ISMSC) 2022 14
5.1.3 Calix 2022 15
5.2 Analysis *15*
5.3 Limitations *15*
Section S6 Ethical approval 17
Section S7 Data 18
7.1 Examples of data from Study 3 participants at ISSS 2022 ..... *18*
7.2 Online note board from ISMSC 2022 ..... *24*
7.3 Examples of data from ISMSC 2022 ..... *25*
7.4 Online note board from CALIX 2022 ..... *43*
Section 8 Tables 44
Table S1: Categorized sources by main theme. *44*
Table S2: Summaries of 10 texts included in category one from the 158 found in the semi-systematic literature review. *45*
Table S3: Codebook from Study 1. Child codes indicated in italics. *48*
Table S4: Full list of sources found in Study 1 by category. *51*
Table S5: Full list of survey questions. *63*
Table S6: Study 1 participant demographics. *65*
Table S8a: Correlations between being First Gen & feeling financial burden. *79*
Table S8b: Correlations with feeling financial burden. *80*
Table S8c: Correlations being First Gen & receiving family support. *81*
Table S8d: Correlations with receiving family support. *82*
Table S8e: Correlation of no support and being First Gen. *83*
Table S8f: Correlation of isolation with imposter syndrome. *84*
Table S8g: Correlation of UG with caring responsibilities. *85*
Table S8h: Correlation of MCR with moving country. *86*
Table S8i: Correlation of gender and online resources. *87*
Table S8j: Correlation of MCR with caring responsibilities. *88*
Table S8k: Correlation of peer influence with family support. *89*
Table S9: Cross-tabulation of gender and career stage. *90*
Section S9 Supplementary References 91

Section S1 First Gen, 'Underrepresented Minorities', and belonging

First Gen students can be defined as those who are the first in their family attending university/college, completing an undergraduate degree within higher education.[85] However, there are alternative definitions of First Gen: Some define First Gen as students whose family members have no postsecondary education at all;[86, 87] while others broaden the definition to include students whose family members have at least some postsecondary education but without completing a degree.[11, 88-91] Another ambiguity is the meaning of 'family'; the term mainly refers to parents yet sometimes includes siblings or is not defined. However, the education of more extended family such as grandparents, aunts or uncles can also influence students' academic careers.[92] Because the definition of First Gen is inconsistent, the way data are collected and interpreted is also inconsistent[85] which leads to a muddled understanding of the barriers experienced by First Gens.[9]

Being First Gen is often conflated with markers for low socioeconomic status and correlated with other forms of marginalization. First Gens disproportionately come from marginalized racial and ethnic groups and low-income backgrounds.[93, 94] Indeed, some authors include First Gen status when using the term 'underrepresented minority' (URM).[16, 95] However, URM is most often used to refer to racial and ethnic identities.[96] Although the impacts of being marginalized due to being First Gen, a minority racial or ethnic background, and/or low socioeconomic status are closely associated, they are not the same. Different combinations of these characteristics will result in unique experiences.[96] Factors including gender, race, transfer status, faculty-student interactions, frequency of study groups, dependent children, institution type, goals and aspirations, high school grades, high school science, and high school mathematics all contribute to differences in outcomes between First Gens and non-First Gens in science.[13] Bettencourt et al. tried to understand how students interpreted being First Gen. They showed students saw being First Gen as an organizational and familial rather than a social identity.[60] In addition, many shared they were unaware of being First Gen until informed by others. This indicates First Gens may not see themselves as an underrepresented minority within wider society. Additionally, being First Gen is not a characteristic that can be seen or attributed by others, which changes the issues/barriers which confront people. These two factors underline why it is necessary to distinguish between URM and First Gens and clarify what is included when discussing being First Gen and other marginalized characteristics to allow for intersectionality.

First Gens are known to experience barriers not felt by their non-First peers as they enter higher education,[97] specifically in science or STEM (Science, Technology, Engineering, Maths/Medicine) subjects.[15, 16, 20] These additional barriers have been reported to reduce STEM course completion rates by up to 31%.[15] These barriers include, but may not be limited to: reduced faculty interaction when compared to non-First Gens;[98] increased family responsibilities;[98] lack of financial resources limiting available study time;[100] reduced ability to access and decode information that enables informed academic and social decision making (e.g.

anticipating costs and academic requirements, general preparation, and acquiring familial support);[101] combined with the lack of advice and council to guide a student throughout their studies.[102]

One explanation proposed for these correlations is a deficit of social and cultural capital. Social capital, cultural capital, and 'habitus' are tools used by Pierre Bourdieu[103] to conceptualise the feeling of belonging:

> "Habitus describes the individual's way of seeing, interpreting and acting in the world, in accordance with their social position. It is internalised and consolidated in childhood through family and educational structures and circumstances. Bourdieu's field conceptualises structured social space within which social agents - individuals, groups, institutions act i.e.: employ strategies to hold or enhance their position. Their position is determined by capital, a concept fundamental to Bourdieu's project of demonstrating how social inequality is reproduced in both economic and symbolic spheres. Cultural capital is acquired over time and through exposure to a particular habitus and is embodied in the practices of social agents. It can enable an individual to navigate a field, knowing the 'rules of the game'"[104]

Pierre Bourdieu conceptualized the role different types of capital play in maintaining social inequalities.[103] Cultural capital can be understood as the degree of ease and familiarity one has with the dominant culture in a society.[105] Access to human and cultural capital is gained through one's networks, and a lack of human and social capital could give First Gens disadvantages compared to their non-First Gen peers because they '*experience considerable limitations in accessing and decoding information for making pertinent academic and social decisions*'.(*15pp35*) This in turn leads to difficulties adapting to college.[106] Uche et al. identified that for First Gens *"having advisors and counsellors who can guide them in choice of classes, and other academic decisions will improve their chances of achieving success in STEM"*.(*15pp35*) Students with less social capital and lower socioeconomic status '*may have a more difficulties regarding transitioning and being academically successful in a STEM institution of higher education*'.[52] It is clear from the literature that feeling connected and building social capital are deemed to be essential for First Gens if they are to persevere and complete undergraduate studies. The higher rates of student attrition found for First Gens and other marginalised groups are also associated with feelings of not-belonging. When individuals are or feel marginalised, they will feel as though they do not belong.[107] While there are many theories of belonging,[108] individual experiences are multi-faceted, and the amount a person feels they belong is subject to change.[109] Aspects of belonging and identity have to be considered intersectionally: "In order to encourage greater diversity in STEM, it is vital that students feel that they have a place in the discipline and that they belong. This demands that they witness people like them succeeding and progressing in STEM careers."[110]

Section S2 Theoretical framework and methodological approach

A theoretical framework in a study of this kind "conveys the deepest values of the researcher(s) and provides a clearly articulated signpost or lens for how the study will process new knowledge".[84] The overarching theoretical approach for this study was Embodied Inquiry.[44] When used as a theoretical framework, Embodied Inquiry enables rigorous research design, data generation, analysis, and dissemination that align with the values of the researchers and the aims of the research. Unlike more conventional social science methodologies and approaches, Embodied Inquiry breaks down traditional dynamics of power. Embodied Inquiry is a means of listening closely; capturing robust data to understand the impact of barriers and how to overcome them while allowing people to process their own experiences. Here, we used Embodied Inquiry to develop an analytic approach that was reflexive, inductive, and iterative to enable understanding and build knowledge. Embodied Inquiry adds depth, richness, honesty and emotion; facilitating the capture and connection with voices whose stories are less often heard, or subjects that are challenging to put into words. It is perfectly suited to explore personal experiences that relate to work: "Combining social science with scientists ensures that the work that is carried out humanises the reasons why this work is important, highlighting the parity and diversity of experience both men and women face whilst maintaining the rigour and validity of the research within the scientific community and beyond."[45, p11577] When disseminating research from an Embodied Inquiry the aim is to do so in a way that allows audiences to emotionally engage with stories of intersectional marginalisation.

This study builds on previous work that aimed to explore and understand individual experiences of barriers to equality/equity, inclusivity, and diversity in science,[45] capture lived experiences of managing research through COVID-19,[46] and explore the intersectional lives of women in STEM through reflective, creative methods and collaborative autoethnography.[43] The conception of the study and the focus on the lived and embodied experiences of First Gens arose from the authors' positionality together with discussions of the barriers and obstacles for specific groups. Embodied Inquiry has also been used successfully as a means of developing reflexivity with doctoral students to address attrition and support progression.[47]

The combination of methods was designed to ensure the research was participatory and meaningful to both First Gens and non-First Gens: "Incorporating these techniques of data capture and analysis allows us to acquire and disseminate a picture of what people are feeling and experiencing even when those emotions and feelings are not easy to put into words. It allows us to research *with* rather than *on* our community."[43,p2] In addition to generating data, participation in the research encouraged awareness and reflection on aspects of identity and the ways in which people might experience different forms of privilege and barriers. Combining a semi-systematic literature review with an online survey that could be analysed both quantitatively and qualitatively and in-person reflective workshops allowed us to gather multi-modal data, conduct a reflexive, inductive, and iterative in-depth mixed-methods analysis, and present an evocative multi-layered account. Together, these data constitute an authentic picture of feelings about being a First Gen scientist across different career stages, and how being First Gen intersects with other barriers to progression such as gender.

Section S3 Study 1

3.1 Semi-systematic literature review

While many literature reviews in social sciences take a narrative approach through a reasonably comprehensive and critical reading of a field, systematic reviews are more common in traditional sciences. A systematic review applies a transparent and replicable methodology to identify and review literature. A semi-systematic literature review allows "*an emphasis on such features as transparency about searching, and the potential for comprehensiveness".*[111] This semi-systematic literature search was mainly conducted via Google Scholar.[112] An additional source for finding relevant literature was the database of the Centre for First Generation Students Success (now known as First Gen Forward).[113] Searches were structured through the use of different keyword combinations including: supramolecular chemistry; chemistry; STEM; academia; science*;* academic roles and identities; university transition; First Gen. In total, 129 keyword combinations were used for the literature search. The results were then filtered by publication period: 2000-2009; 2010-2014; 2015-2022; and 2022-2024. This enabled us to curate a database to answer questions relating to research activity with respect to time. **Table S1** summarises the numbers of texts found organised into key themes. The criteria for inclusion to category one 'Included texts' was specific reference to First Gens in chemistry. Inclusion to categories three and four required specific reference to First Gens in science or STEM. Sources in categories two, five, six and seven included texts concerned with women and careers, First Gens in general, racism, URMs, intersectionality, mental health in science, retention and the like. Each source was examined for inclusion or exclusion into each category by three independent reviewers. Only 10 texts met the inclusion criteria for category one (see **Table S2**). The remaining sources were either excluded (10) or kept as background literature (137). The background literature was read and coded as it was deemed relevant though not specifically concerned with First Gens *and* Chemistry. In total, 147 texts were analysed and informed our methodological design of studies 2 and 3.

3.2 Analysis

All 147 pieces of literature were coded reflexively to identify themes using NVivo software. A generative or inductive approach was used to create codes based on the key themes identified. This resulted in a codebook of 'parent' and child' codes, see **Table S3**. Four overarching themes specific to First Gen experiences were identified, and these informed the design of the online questionnaire for Study 2 and areas to focus on in Study 3. These were:

1) Barriers for First Gens and how they are the same or different to barriers faced by other marginalised groups;

2) Capital (social and cultural);

3) Academic support, and support from an institution;

4) Access to resources for support.

The 137 texts categorized as background literature were further categorized into the following themes based on their content: Academia, STEM, Chemistry, EDI, Women, and Mentoring.

Section S4 Study 2

4.1 Online survey

The online survey was launched in 2022 and was open to respondents for 12 months. The link was shared on the WISC website,[114] promoted on social media platforms such as X (formally Twitter) and at in-person disciplinary conferences and events. Participants were asked 25 questions, provided in **Table S5**.

The first 10 questions were used to collect broad demographic data. The next seven questions were informed by Study 1. We asked participants about their fears, apprehensions and general experiences of studying and researching chemistry. The questions explored how individuals made choices on what to study, finances, where they found support and careers advice which had all been identified as areas in which First Gens faced additional barriers in Study 1. The final nine questions were generated by the WISC community and were also reflected in the literature and asked participants to reflect on feelings of belonging and alienation, self-consciousness or awareness of First Gen status, as well as mobility, supervision, and support. Our aim was to generate new knowledge by focusing on broader scientific researchers rather than solely focus on student cohorts.

4.2 Participant demographics and descriptive statistics

In total, 136 participant responses were collected and carried forward for cleaning and analysis. The first stage of cleaning the data consisted of categorising the demographic questions for statistical analysis in SPSS. The demographic questions collected open text information on participants' geographical location, mobility, caring responsibilities, and career stage. As identity characteristics are not always easily encapsulated by tickboxes[115-117] even when following best practice,[118] these questions gave the participants an opportunity to put answers in their own words. An example of the categorizing process is participants' career stage. We grouped PhD students and Masters students as Post-Graduate Researchers (PGR). Full Professors and Group Leaders were categorized as Late Career Researchers (LCR). Associate/Assistant Professors, Lecturers and Junior Group Leaders were categorized as Mid-Career Researchers (MCR). Other answers such as Research Fellows and Post-Doctoral Fellows were grouped as Early Career Researchers (ECR). The categorization of responses was particularly important when it came to asking whether an individual identified as being First Gen or not. As detailed in the supplementary text, the idea of being First Gen is not always one that individuals identify with; it can be complicated, or the individual might feel conflicted about their status:

> *"My dad did go to university but dropped out; my mum went to some sort of post-school college - maybe drama? - but not degree awarding."*

> *"My parents didn't go to university, but I think that I would consider their education level university level. I never really thought about it."*

“My father started a study but only for one term, then he quit. So I think I'm still the first?”

“My older sister was the first of my direct family, but we're the same generation. I am not so sure for my more distant family, to whom I don't have much of a contact, so I wouldn't count them”

“My father did an apprenticeship as a carpenter, and I don’t[sic] really know if this counts as higher education.”

“I am the first to study Chemistry. My parents studied Microbiology and Biology”

Of these responses, all bar the last were categorized as being First Gen. In addition to coding and categorizing the open text response to the question asking whether an individual was First Gen, the team checked responses for internal consistency and re-allocated categories where necessary. For example, if a respondent ticked ‘yes’ to being First Gen then later said “*As I am not a first gen…*” their response was corrected to not being First Gen for the first question. A note was made on the data set every time a correction such as this was applied. Detailed categories were created for race/ethnicity as well as a broader white/non-white category. Geographical locations for where participants were born/raised and lived/worked were grouped into continents. Yes/no dummy variables e.g. ‘work/study in Europe’ Yes=1 No=0 were created for each category to allow statistical analysis of the demographic variables. This cleaned data set was then exported to NVivo and the long-text questions were analyzed. The full demographic information of all survey participants is detailed in **Table S6**. Simple cross-tabulations to show the breakdown of participants are provided in **Tables S6a** - **S6g.**

4.3 Analysis

4.3.1 Qualitative analysis

The long-text responses were analyzed in two stages. Firstly, through a reflexive thematic analysis,[119] then, a further process of reductive categorization to allow later quantitative analysis. Reflexive thematic analysis, like many forms of qualitative research, is subjective. It relies on the reflexivity of the researcher and awareness of positionality. However, unlike quantitative research, the purpose of qualitative analysis is very different as it is aimed at extending understanding rather than generalising results.[120] Our reflexive thematic analysis was informed by the codes generated in Study 1 and identified broad themes such as *“Family support”, “Imposter feelings”* and *“Unfamiliarity”.* These larger themes became ‘parent codes’ containing more specific child codes. For example, “*Unfamiliarity*” became parent to “*Academic challenge*”, “*Funding*”, “*Cultural*”, “*Career progression*”, “*Academic processes*” and the like. The analysis was an iterative process, in that codes generated from Study 1 informed the coding for Study 2, and this framework later informed analysis for Study 3.

Next, each respondents’ long-text answers were categorised into dichotomous variables. For example, answers coded to the theme *“Financial burden”* (parent code) were sub-coded into either *“Yes”* or *“No”* (child codes) depending on whether the participant described experiencing

financial burden or not. These coded data were then exported back into SPSS to determine the statistical significance of any potential correlations. For instance, in the survey question "*Was the financing of your studies an issue that worried you before or during your studies? If so, why?"* An answer such as *"Yes. Had no idea about student finance, I was from a low-income family. I applied for a bursary for women studying science from the Church so that I could buy a laptop, as I had no other way of financing it. I worked two jobs for my whole undergrad"* was categorised as a "Yes". Alternatively, an answer such as*, "Surprisingly, it has not. I have always worked more than one or two jobs and just believed that all would work out if I could find a job. I only had one loan as a undergrad and I just worked to support myself and not burden my parents"* was categorised as a "No". There are clear limitations to this method of reducing long form answers to dichotomous variables. There is a reliance on the subjective interpretation of the researchers and the reduction of detailed, personal information and stories into yes/no answers. These limitations are mitigated by including more traditional narrative qualitative analysis using verbatim quotations from participants in addition to statistical analysis. Detailed examples of the coding and categorization process for different survey questions are given below:

Question: ***"Did you know a lot about career paths for the future generally or in supramolecular chemistry specifically when you started studying? If not, where and when did you get this information?"***

Response: *"I wasn't prepared for how tough I found the Masters degree and I didn't know that a PhD was a research position through working until I was in the work/lab environment"*

Code ***-> 'Unfamiliarity'*** **Child code** ***-> 'Academic Challenge***

Question: ***"Who did you go to for the resources and support that you needed to begin your studies or academic career?"***

Response: *"My parents supported me economically but without having a lot of resources for themselves and friends or family supported me emotionally but without understanding the real challenges."*

Code ***-> 'Family support'*** **Child code** ***-> 'Financial'***

The term *"a lot of resources"* is vague and could be defined and used differently depending on someone's previous life experience and personal views. In this quote, the respondent described emotional support from family and how they felt this was limited due to lack of understanding. The answer was thus also coded:

Code -> '***First Gen disadvantages'*** **Child code** ***-> 'Family understanding'***

Question: ***"Has there been anything that feels unfamiliar or alien to you regarding your academic studies or career?"***

Response: *"This is my first time being full-time at a 'brick' university, so a lot of things were unknown. 'Academia' still seems quite alien but I am getting to know more about it from my supervisor and fellow group members and general observations. I am worried about my career prospects once I complete my DPhil, but I am trying to network as much as I can so I can find out about potential opportunities and options for when that day comes."*

Code -> *'Unfamiliarity',* child code -> *'Career progression'*

This answer was coded in this way because the answer is primarily concerned with career progression. However, due to the limitations of this method, this coding essentially hides the participant's statement, *"a lot of things were unknown.".* The full list of codes and child codes is given in **Table S7**.

4.3.2 Quantitative analysis

In order to ascertain the significance of the relationships between the demographic we created dichotomous variables for the following:

financial_burden	do they feel a financial burden? (Yes=1 No=0)
confidence	do they struggle with confidence? (Yes=1 No=0)
networking	do they struggle with networking or seeking help? (Yes=1 No=0)
imposter	do they have imposter syndrome? (Yes=1 No=0)
isolation	do they struggle with isolation (Yes=1 No=0)
peers	peer influence (Yes=1 No=0)
seniors	occupational and or academic seniors (Yes=1 No=0)
online_res	online resources (Yes=1 No=0)
family	family support (Yes=1 No=0)
unfamiliar	unfamiliarity categorised (Yes=1 No=0)

We created dummy variables for the demographics as follows:

Career_ESR	dummy for early stage researcher (Yes=1 other career stages=0)
Career_MSR	dummy variable for mid-stage researcher (Yes=1 other career stages=0)
Career_PG	dummy variable for PG students (Yes=1 other career stages=0)

Career_LSR dummy variable for Late stage researchers (Yes=1 other career stages=0)

Career_UG dummy variable for undergraduates (Yes=1 other career stages=0)

Career_other dummy variable for other (Yes=1 other career stages=0)

Gender_man dummy variable man (Yes=1 No=0)

Gender_woman dummy variable woman (Yes=1 No=0)

Gender_other dummy variable other (Yes=1 No=0)

Moved_None dummy for did moved country (Moved country=1 did not move=0)

4.3.3 Regression Analyses

We used linear regression as a linear probability model to identify correlations within those data collected.[59] A summary of the correlations within the data set is provided in **Tables S9a - S9d**.

As shown in **Table S8a**, First Gens are 28.3% more likely to feel a financial burden than non First Gens, and this is highly significant. This effect could be confounded by gender (due to the gender pay gap), protected characteristics (there are pay gaps for protected characteristics), caring responsibilities (which place an additional financial burden on people), and moving country (there are additional costs involved). The connections to being First Gen for these confounding variables remain the same. As can be seen in **Table S8b**, the impact of being First Gen increases in the presence of the confounding variables, staying significant and rising to a 28.6% impact. Being a woman or other minority gender makes it more likely that respondents will feel a financial burden but this is not significant. Similarly, having a protected characteristic, caring responsibilities, or moving country make it more likely that a respondent will feel a financial burden, but the effect is not significant.

First Gens are 25.8% less likely to say they have family support. Therefore, lack of family support is shown to be very impactful on the First Gen students included within this study (**Table S8c**). As shown in **Table S8d**, we wanted to know if lack of family support could be compounded by gender (men may be more likely to receive family support and less likely to be First Gen) having a protected characteristic (there is an overlap with being First Gen and other marginalised characteristics and they may require more support or be less likely to be accepted by their family and get support), caring responsibilities (they may be more driven to study and they may need more family support) and moving country (they may be more determined to succeed and be less able to get family support). In summary, our findings show that being a man meant that respondents were less likely to receive family support, but this was not significant. Having a protected characteristic or caring responsibilities meant respondents were

slightly more likely to receive family support but these were not significant. Moving country meant that respondents were less likely to receive family support but again this was not significant. These findings mean that being First Gen is not confounded by these control variables, and that it is a factor in being less likely to receive family support.

Other significant findings (**Tables S9e – S9k**) included that First Gens were 9% more likely to say they had no support at all than non First Gens (**Table S8e**). People who felt isolated were 42.8% more likely to say they felt imposter syndrome (**Table S8f**). Undergraduates were 33.6% were less likely to have caring responsibilities (**Table S8g**). Mid career researchers were 25% more likely to have moved country (**Table S8h**). Men were 12% less likely to use online resources than women and other minority genders (**Table S8i**). Mid career researchers were 44.5% more likely to have caring responsibilities than those at other career stages (**Table S8j**). People who said they were influenced by their peers were 20.3% less likely to say they had family support (**Table S8k**).

4.4 Limitations

The limitations of the survey are that it was a relatively small, self-selecting sample. Women were overrepresented (see **Table S9**), as they comprised 55% of respondents, yet women are considered to be underrepresented in chemistry and science, and this underrepresentation increases with career stage.[43] This was to be expected given that the survey originated from the Woman In Supramolecular Chemistry (WISC) network.[109] Other limitations could be that the sample was predominantly formed of supramolecular chemists, and as such their experiences may not be representative of scientists as a whole. This clearly shows that the sample has a higher than expected proportion of women at most career stages (27.2-73.1%). The sample is predominantly postgraduate research students (44.9%) which is unsurprising given that the survey was advertised and promoted at conferences and symposia targeted at research students and early career researchers.

Section S5 Study 3

5.1 Reflective workshops

5.1.1 1st International Supramolecular Summer School (ISSS)

The first workshop was held in-person at the 1st International Supramolecular Summer School in July 2022 in Cagliari, Italy. Attendance was limited to 50 early-career supramolecular chemists of all genders; mainly graduate research students with some post-doctoral researchers. The majority came from Europe though there were attendees from USA. The reflective workshop was scheduled as part of the main programme. The first section of the workshop was a presentation on WISC activities and research, during which some creative non-fiction vignettes drawn from research were shared with the audience.(*58*) The rest of the workshop was more interactive. Participants were given pens and coloured sticky notes and were provided with the following prompts:

1. **What does First Gen mean to you?**
2. **What is specific to a science journey?**
3. **What could be in that 'hidden handbook'?**

The 'hidden handbook' refers to the idea that those who were not First Gen had access to information that First Gens did not and that this information eased their career path and progression. It was also linked to ideas of what success meant as a supramolecular chemist and/or scientist. Forty-Seven sticky notes were collected from this workshop. See **Figure S1** for example data.

5.1.2 International Symposia on Macrocyclic and Supramolecular Chemistry (ISMSC) 2022

The second workshop was held in-person at the 2022 annual International Symposia on Macrocyclic and Supramolecular Chemistry (ISMSC2022), in Eugene, Oregon (USA). The reflective workshop was programmed into the five-day long conference, timetabled for early-afternoon on the only short day of the event. It was open to all conference delegates and attendance was voluntary. Despite this being the only afternoon free from research talks, around half the 500 delegates chose to attend the session. The conference included postgraduate researchers, post-doctoral researchers, research fellows, early and mid-career independent researchers, as well as established academics from USA and international institutions e.g. Australia, China, and UK. The delegates had access to coloured pens, paper, and a virtual note board. They were given three prompts:

1. **What are the barriers and opportunities you have?**
2. **What can be done to address these barriers and challenges as a community?**
3. **In your view what are the specific challenges for First Gen chemists?**

After each prompt they were given time to think on their own, write, draw, or mark make, contribute to the virtual noteboard (**Figure S2**), and talk to the people near them before discussing as a whole group. Unlike the first workshop, the prompts here allowed for a broader discussion of intersectional barriers, opportunities and challenges. As can be seen in **Figure S2**, some participants chose to include images of their hand-written or hand-drawn notes on the online noteboard. In addition to the online noteboard, 59 hand-drawn notes were collected and are displayed in **Figure S3**.

5.1.3 Calix 2022

The final workshop was held as part of the 16th International Conference on Calixarenes (Calix 2022), in New Orleans (USA). It was not possible to attend the event in person, and so the workshop was delivered remotely, with around 30 participants in a room and the facilitators joining in a video call. As in the Summer School, the first part of the workshop was an overview and introduction to WISC's activities and research. The event was aimed at addressing EDI issues, and so the interactive and participatory section included the prompts:

1. **What do you think could be the next steps for WISC? What should we focus on next?**
2. **How can we facilitate more conversations on DEI [Diversity, Equity/Equality, Inclusion] and how can we make these more inclusive?**
3. **In your view, what are the specific challenges facing First Gen chemists in developing their career?**

Responses were collected on a virtual note board shown in **Figure S4**. It was more challenging to engage the attendees in discussion at this event, largely in part due to remote facilitation.

5.2 Analysis

The data from Study 3 was digitally scanned into NVivo and analysed using reflexive thematic analysis, initially against the codes generated in Study 2 which was in turn informed by the codes generated in Study 1.

5.3 Limitations

The limitations of Study 3 are that the participants were potentially unrepresentative of the population of scientists as a whole, and there is no demographic data to confirm or dispute this as attendance at the workshops was not monitored and all responses were anonymous. There were no control workshops given at conferences in other disciplines, in part because the supramolecular chemistry community had already been introduced to the creative, reflective approach used and were receptive to it. The workshops were not intended to be replicated at each event, instead they were designed specifically for the audiences at each. However, there was a large crossover between the populations that participated in the workshops and those

who completed the survey in Study 2, as the survey was heavily promoted during the workshops and conferences. In this case it might be accepted that the views are representative, particularly of early career and marginalised scientists. The workshop at ISMSC 2022 was voluntary, and as a result the participants were self-selected.

Section S6 Ethical approval

This study was given full ethical approval through the University of Kent's Research Ethics Committee in the Centre for the Study of Higher Education in November 2021.

Section S7 Data

7.1 Examples of data from Study 3 participants at ISSS 2022

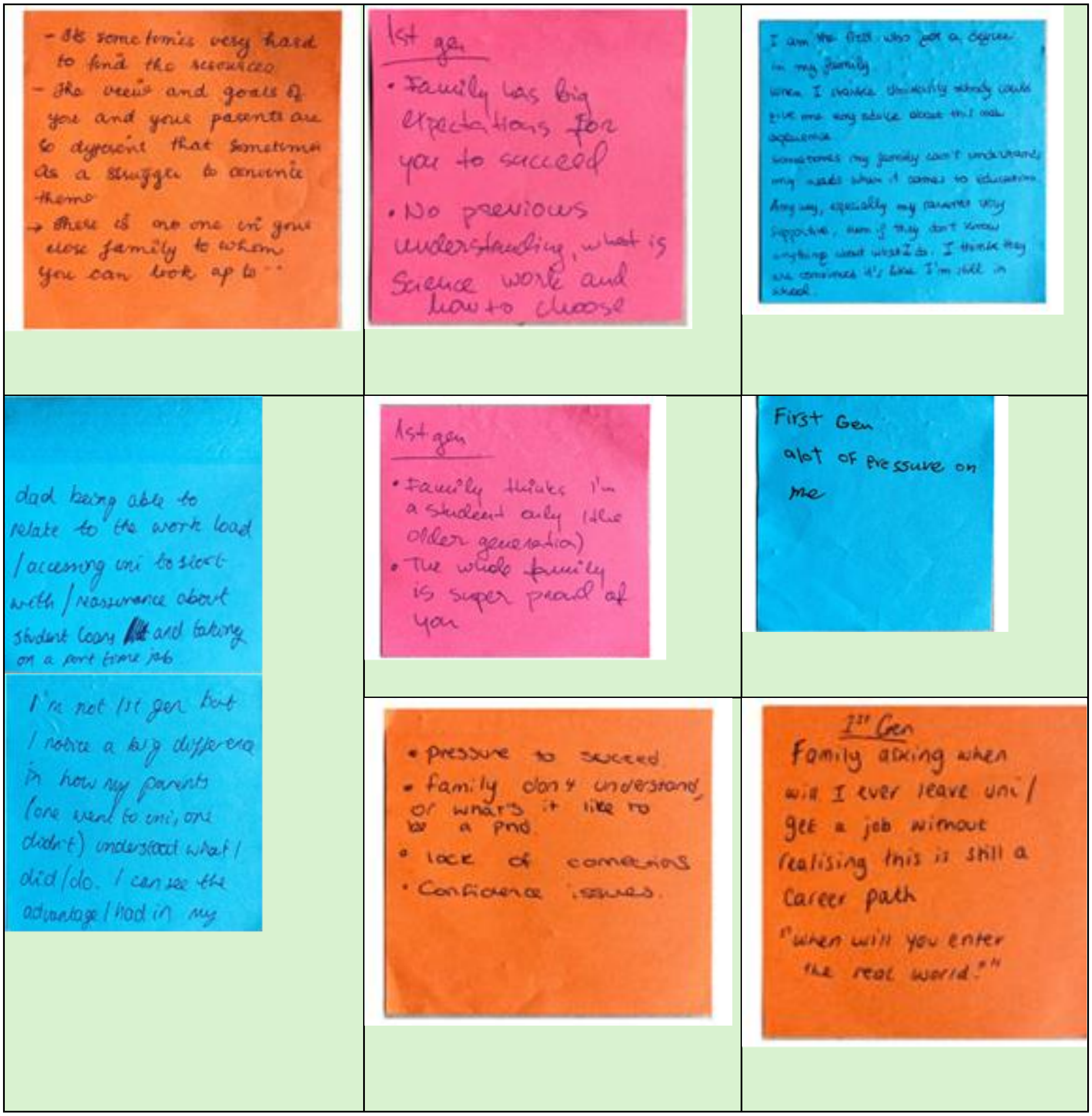

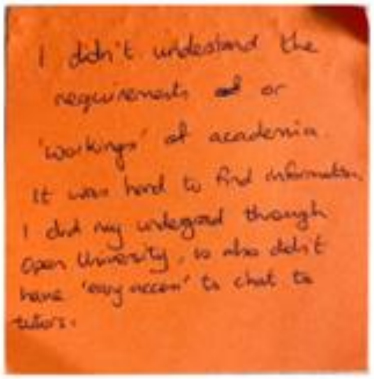
I didn't understand the
requirements or
'workings' of academia.
It was hard to find information.
I did my undergrad through
Open University, so also didn't
have 'easy access' to chat to
tutors.

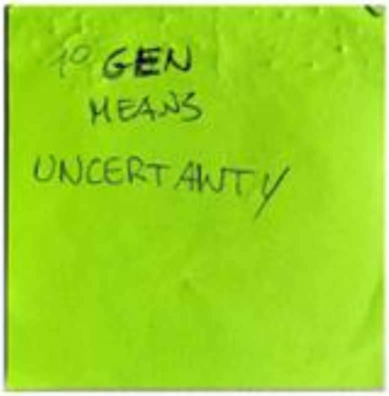
1º GEN
MEANS
UNCERTAINTY

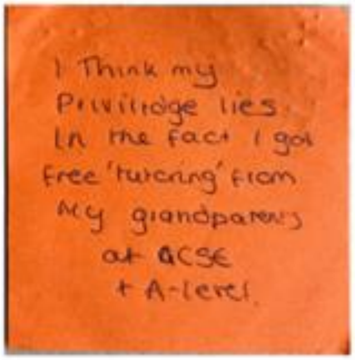
I Think my
Privilidge lies
In the fact I got
Free 'tutoring' from
My grandparents
at GCSE
+ A-level.

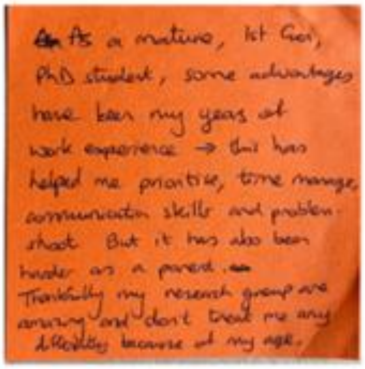
As a mature, 1st Gen,
PhD student, some advantages
have been my years of
work experience → this has
helped me prioritise, time manage,
communication skills and problem-
shoot. But it has also been
harder as a parent.
Thankfully my research group are
amazing and don't treat me any
differently because of my age.

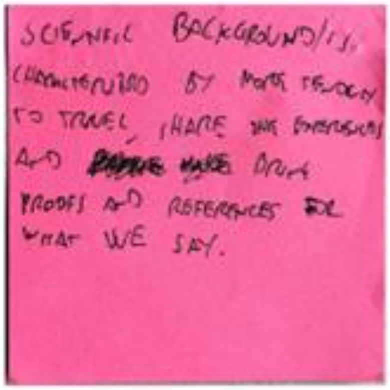
SCIENTIFIC BACKGROUND/S,
CHARACTERIZED BY MORE TENDENCY
TO TRAVEL, SHARE THE EXPERIENCES
AND DEMAND PROOFS AND REFERENCES FOR
WHAT WE SAY.

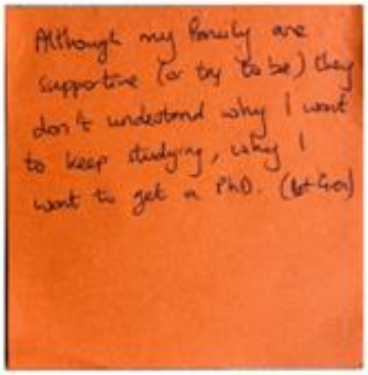
Although my family are
supportive (or try to be) they
don't understand why I want
to keep studying, why I
want to get a PhD. (1st Gen)

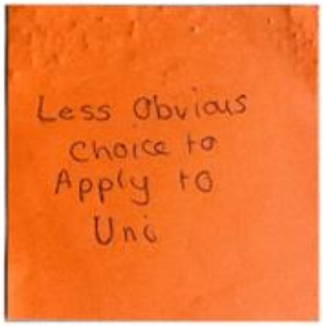
Less Obvious
Choice to
Apply to
Uni

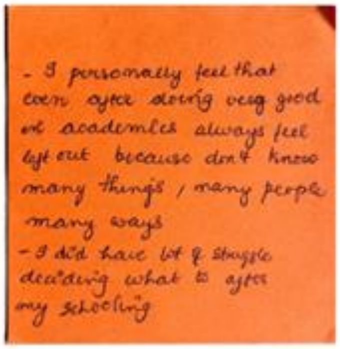
- I personally feel that
even after doing very good
at academics always feel
left out because don't know
many things, many people
many ways
- I did have lot of struggle
deciding what to after
my schooling

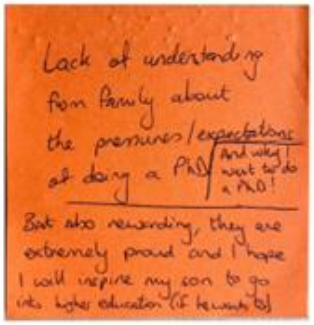
Lack of understanding
from family about
the pressures/expectations
of doing a PhD
And why I want to do a PhD!
But also rewarding, they are
extremely proud and I hope
I will inspire my son to go
into higher education (if he wants to)

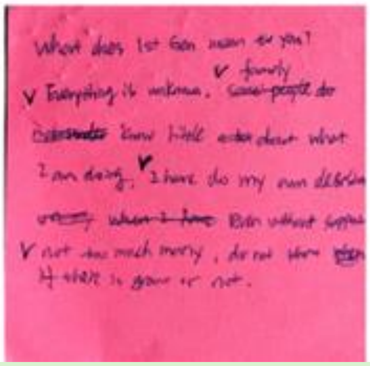
What does 1st Gen mean to you?
Everything is unknown.
I am doing.
not so much money, do not know if there is grow or not.

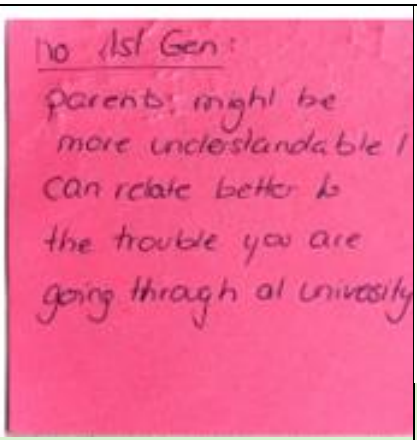
No 1st Gen:
parents might be
more understandable I
can relate better to
the trouble you are
going through at university

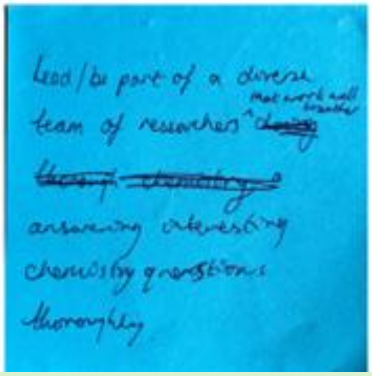
Lead / be part of a diverse
team of researchers
answering interesting
chemistry questions
thoroughly

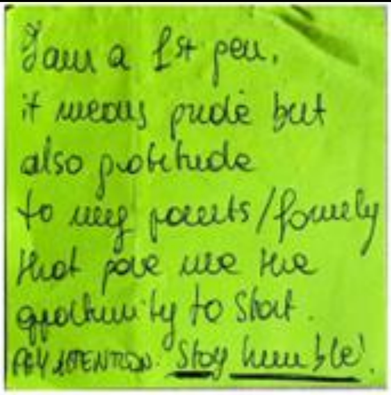
I am a 1st gen.
it means pride but
also gratitude
to my parents / family
that gave me the
opportunity to start.
PAY ATTENTION: Stay humble!

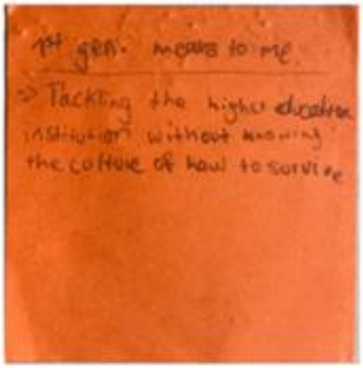
1st gen. means to me
-> Tackling the higher education
institution without knowing
the culture of how to survive

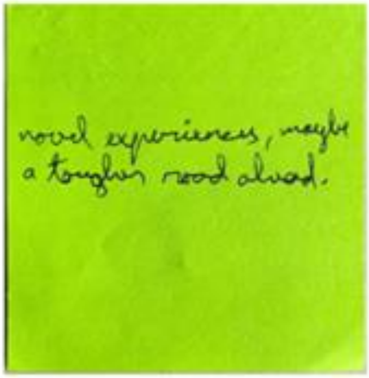
novel experiences, maybe
a tougher road ahead.

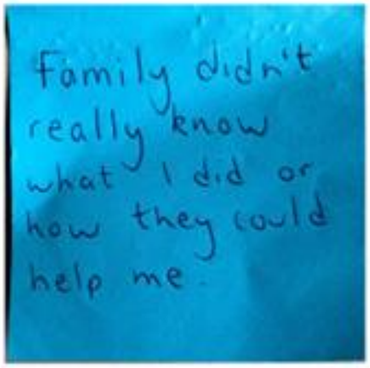
Family didn't
really know
what I did or
how they could
help me.

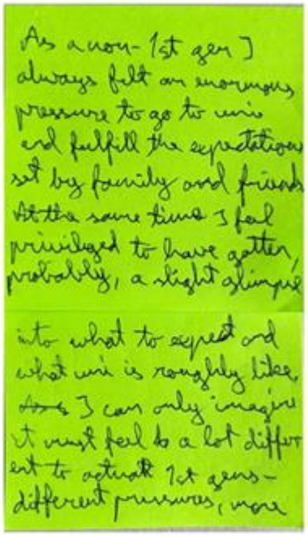
As a non-1st gen I
always felt an enormous
pressure to go to uni
and fulfill the expectation
set by family and friends
At the same time I feel
privileged to have gotten,
probably, a slight glimpse
into what to expect and
what uni is roughly like.
As I can only imagine
it must feel to a lot differ
ent to actual 1st gens -
different pressures, more

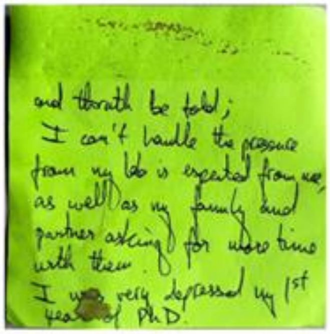
and the truth be told;
I can't handle the pressure
from my lab is expected from me,
as well as my family and
partner asking for more time
with them
I was very depressed my 1st
year of PhD.

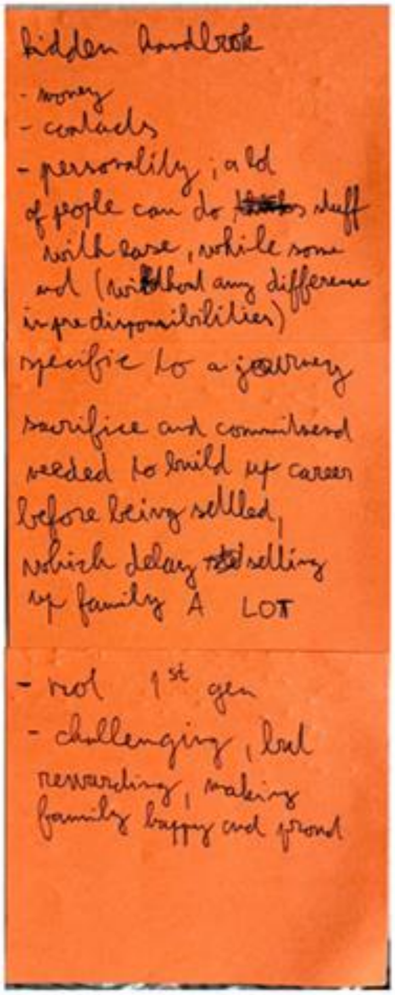
hidden handbook
- money
- contacts
- personality; a lot
of people can do stuff
with ease, while some
not (without any difference
in predispositions)
specific to a journey
sacrifice and commitment
needed to build up career
before being settled,
which delay settling
up family A LOT
- not 1st gen
- challenging, but
rewarding, making
family happy and proud

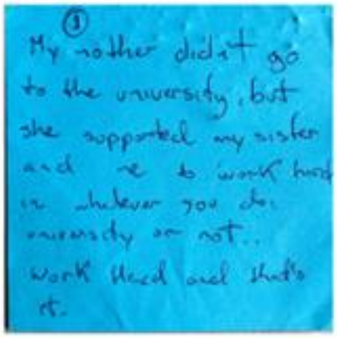
①
My mother didn't go
to the university, but
she supported my sister
and me to work hard
in whatever you do.
university or not..
work Hard and that's
it.

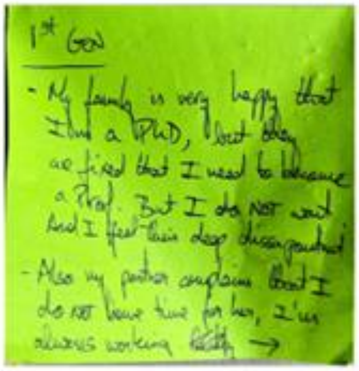
1st GEN
- My family is very happy that
I do a PhD, but they
are fixed that I need to become
a Prof. But I do NOT want
And I feel their deep disappointment
- Also my partner complains that I
do not have time for her, I'm
always working →

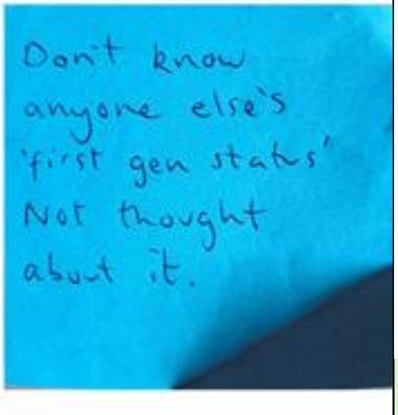
Don't know
anyone else's
'first gen status'
Not thought
about it.

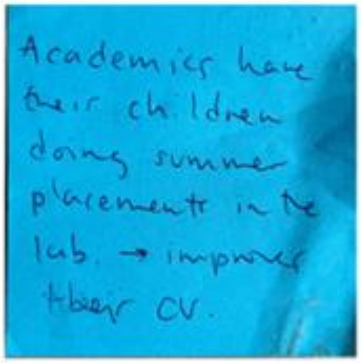
Academics have
their children
doing summer
placements in the
lab. → improve
their CV.

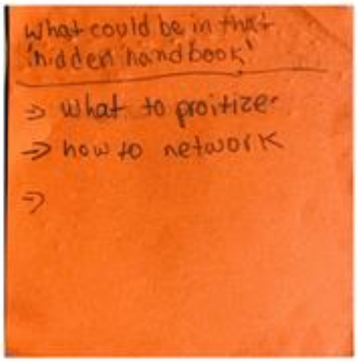
What could be in that
'hidden handbook'
⇒ what to proitize
⇒ how to network
⇒

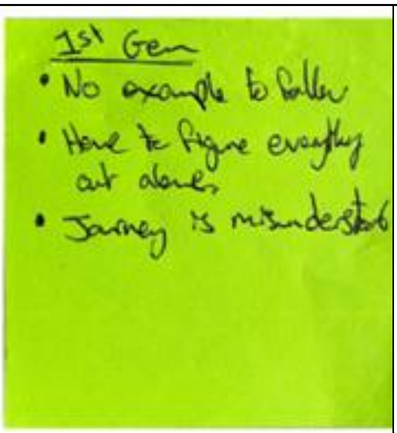
1st Gen
• No example to follow
• Have to figure everything out alone,
• Journey is misunderstood

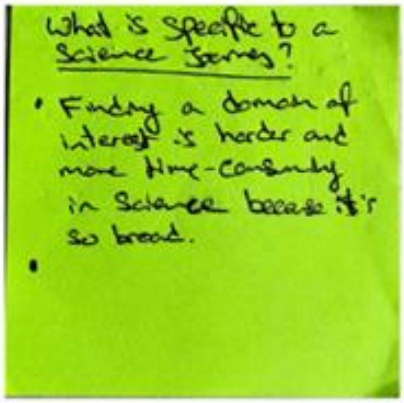
What is specific to a Science Journey?
• Finding a domain of interest is harder and more time-consuming in Science because it's so broad.

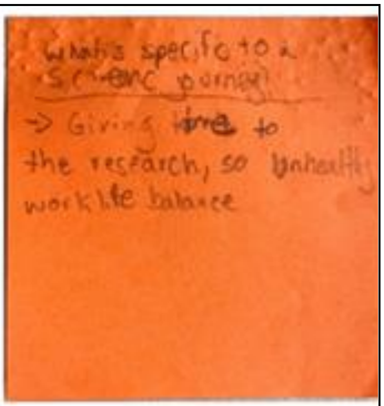
What is specific to a
-> Giving time to the research, so unhealthy work life balance

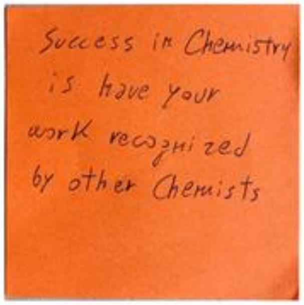
Success in Chemistry is have your work recognized by other Chemists

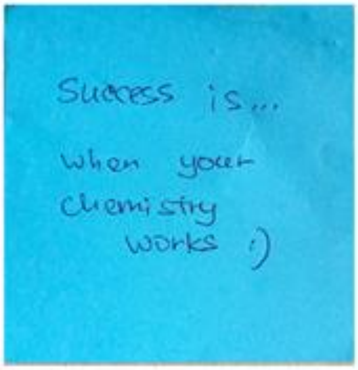
Success is...
when your chemistry works :)

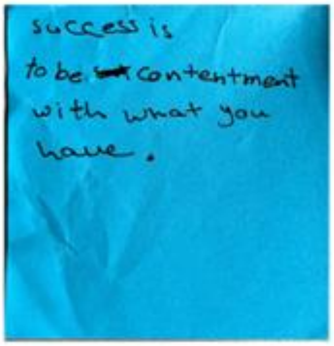
success is
to be contentment with what you have.

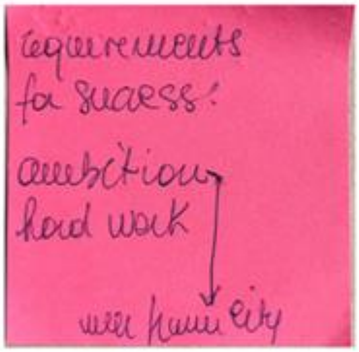
requirements for success:
ambition
hard work

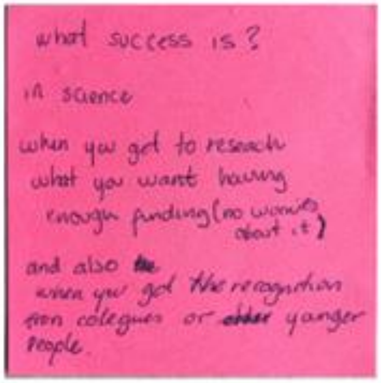
what success is?
in science
when you get to research what you want having enough funding (no worries about it)
and also when you get the recognition from collegues or younger people.

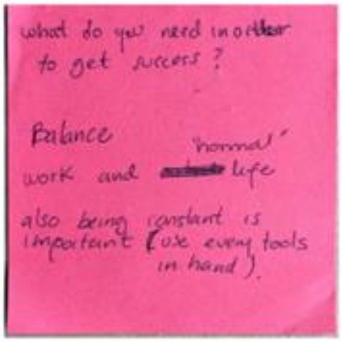
what do you need in order to get success?
Balance work and "normal" life
also being constant is important (use every tools in hand).

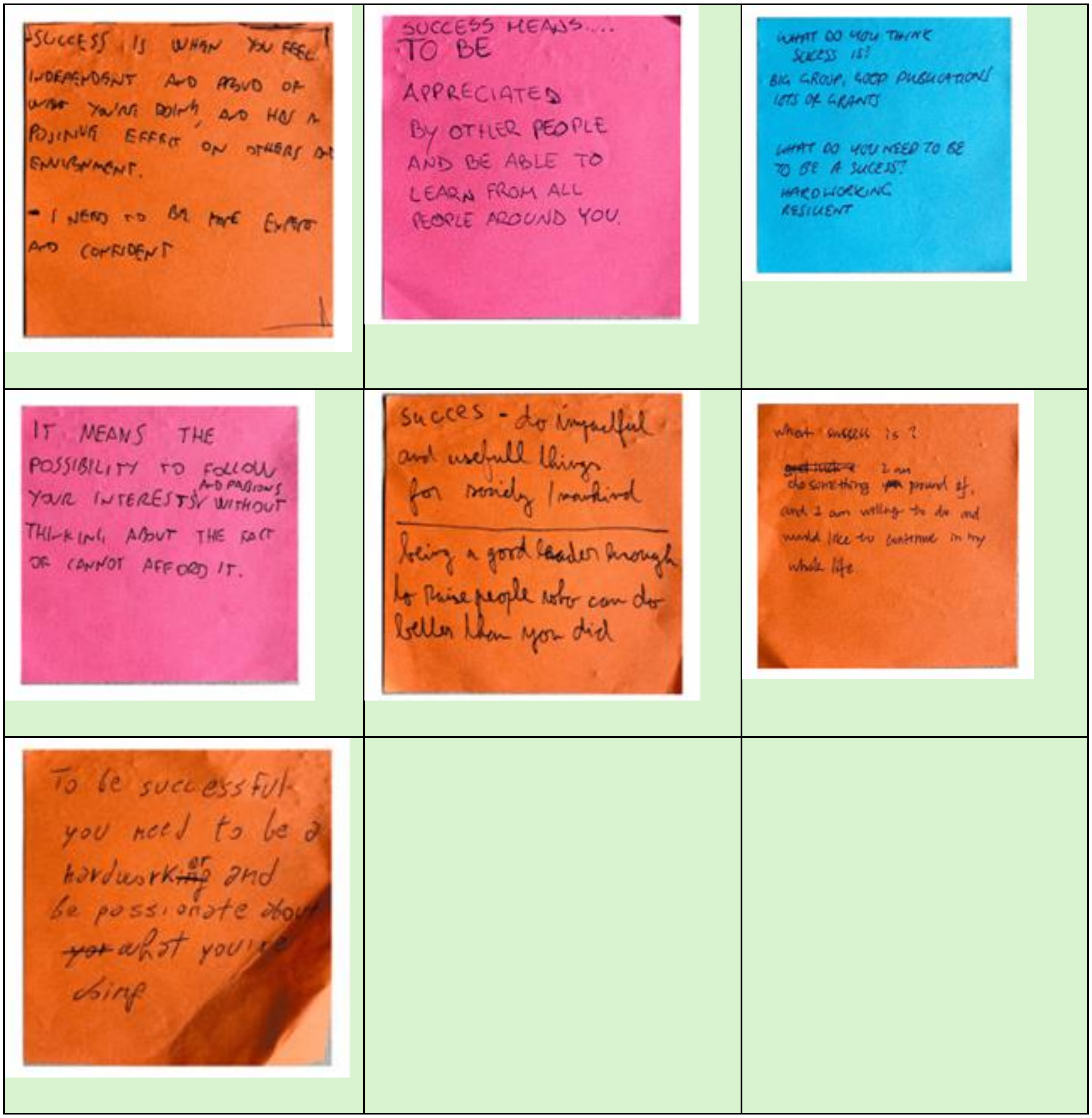

Fig. S1: Examples of data from ISSS 2022

7.2 Online note board from ISMSC 2022

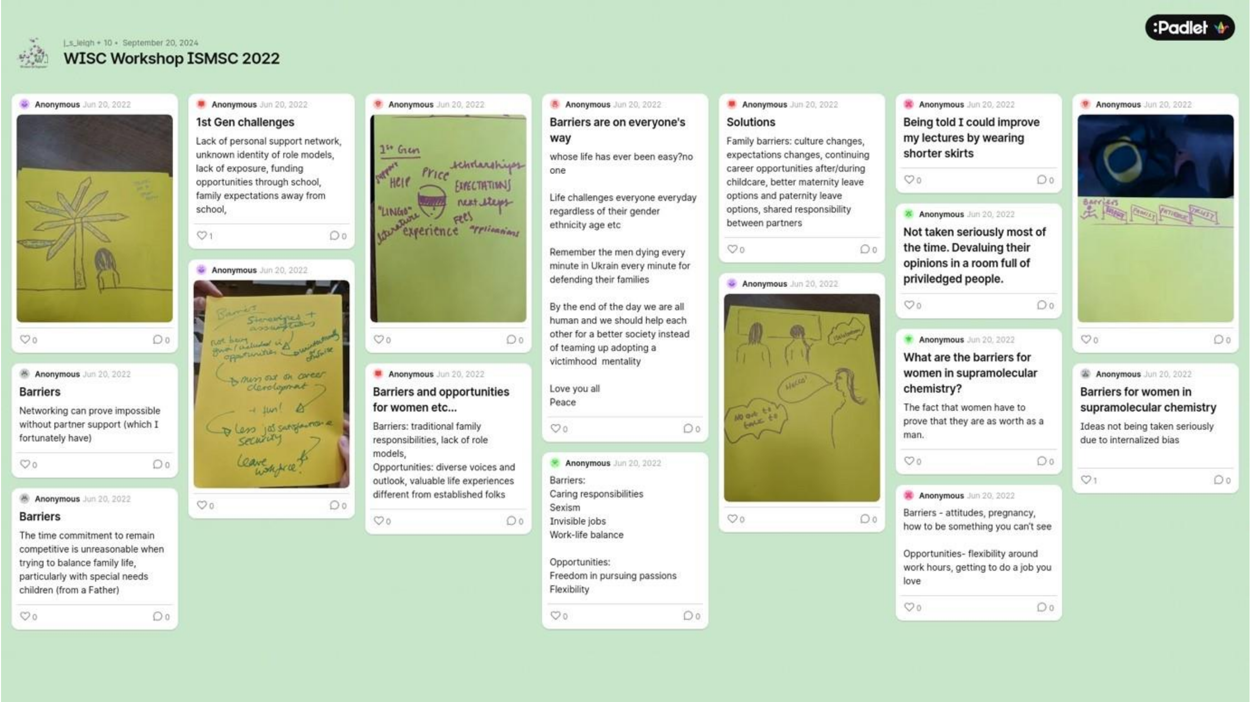

Fig. S2: Virtual note board from ISMSC2022.

7.3 Examples of data from ISMSC 2022

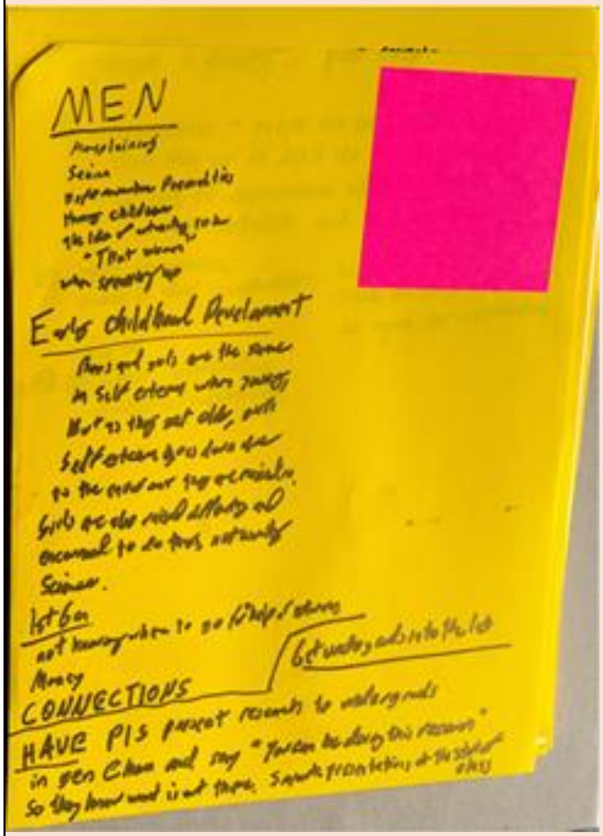

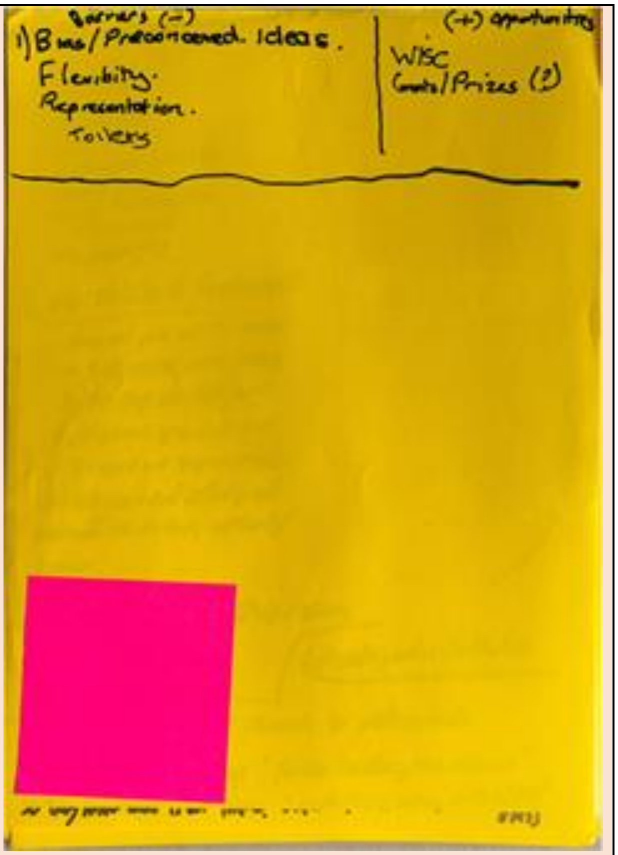

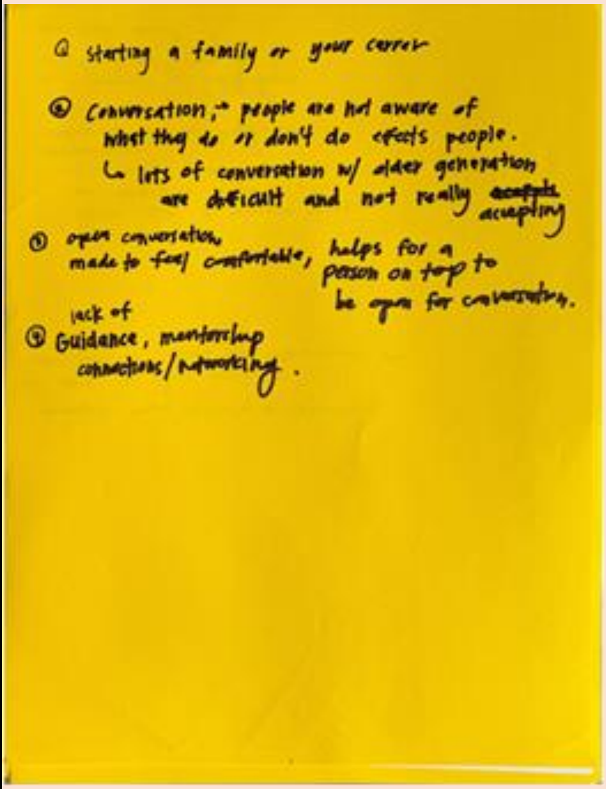

④ starting a family or your career
⑤ Conversation; people are not aware of what they do or don't do effects people.
↳ lots of conversation w/ older generation are difficult and not really accepting
① open conversation, made to feel comfortable, helps for a person on top to be open for conversation.
lack of
③ Guidance, mentorship connections/networking.

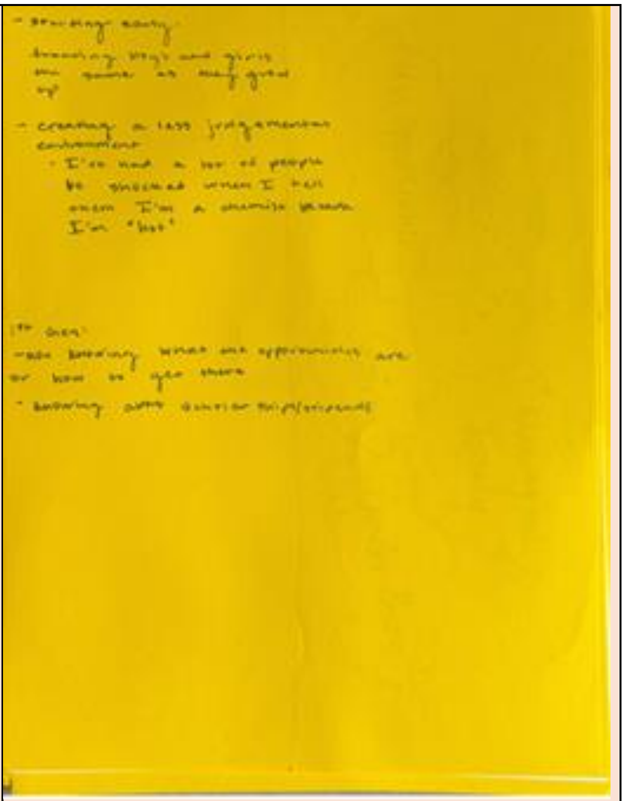

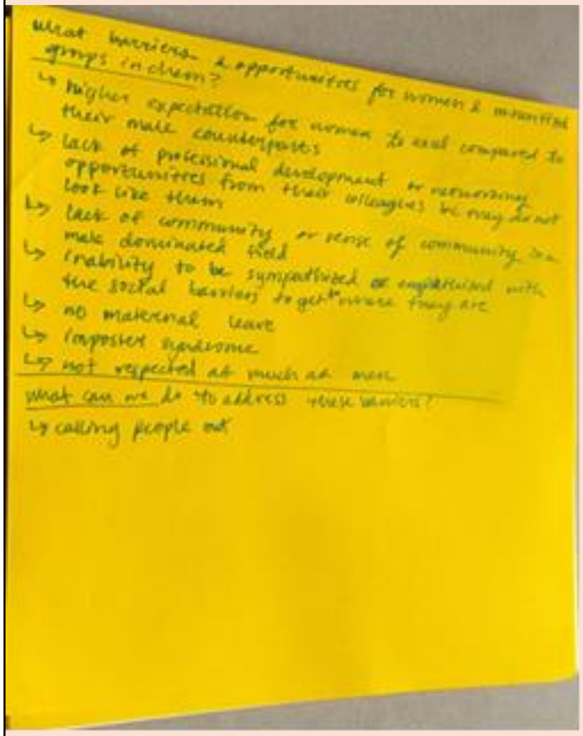

What barriers & opportunities for women & minorities groups in chem?
↳ higher expectation for women to excel compared to their male counterparts
↳ lack of professional development or networking opportunities from their colleagues b/c they do not look like them
↳ lack of community or sense of community in a male dominated field
↳ inability to be sympathized or empathized with the social barriers to get where they are
↳ no maternal leave
↳ imposter syndrome
↳ not respected as much as men
What can we do to address these barriers?
↳ calling people out

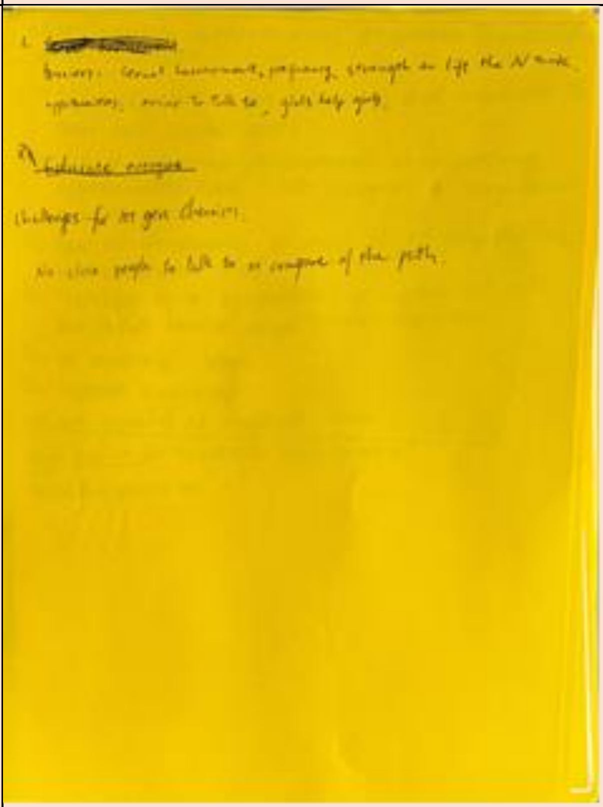

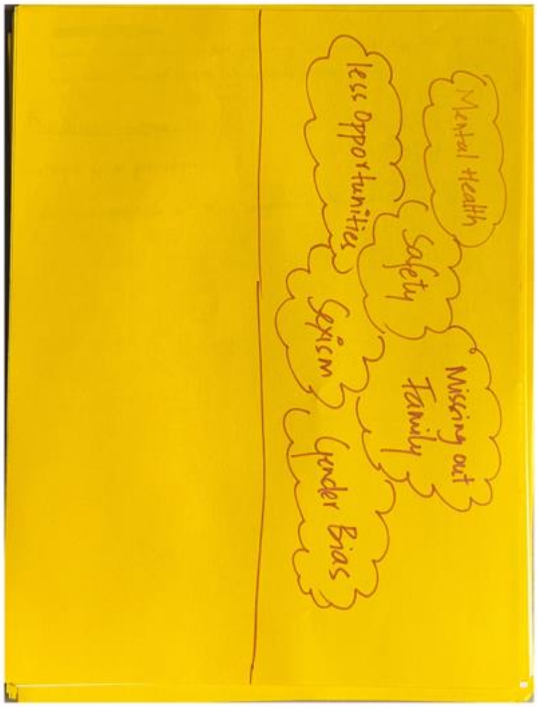
Mental Health
less Opportunities
Safety
Missing out Family
Sexism
Gender Bias

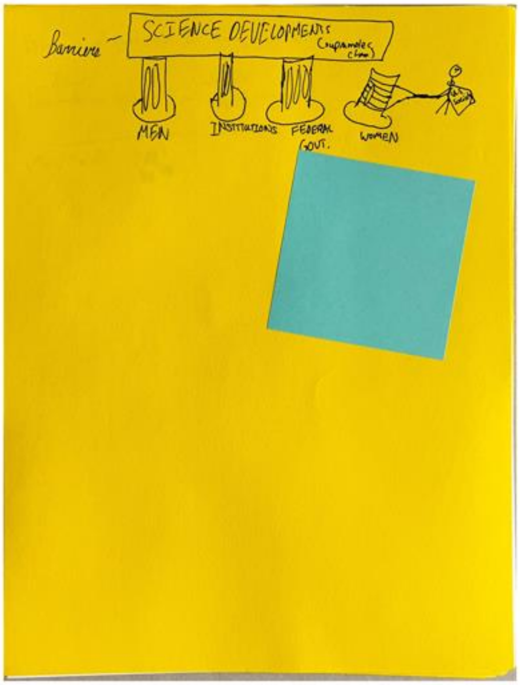
Barriers
SCIENCE DEVELOPMENTS
MEN
INSTITUTIONS
FEDERAL GOVT.
WOMEN

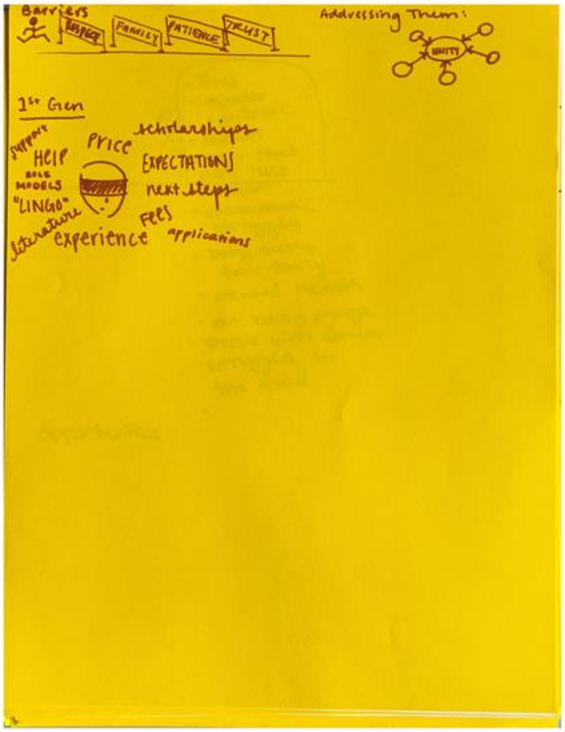
Barriers
RESPECT
FAMILY
PATIENCE
TRUST
Addressing Them:
UNITY
1st Gen
support
Help
Price
scholarships
role models
EXPECTATIONS
"LINGO"
next steps
literature
fees
experience
applications

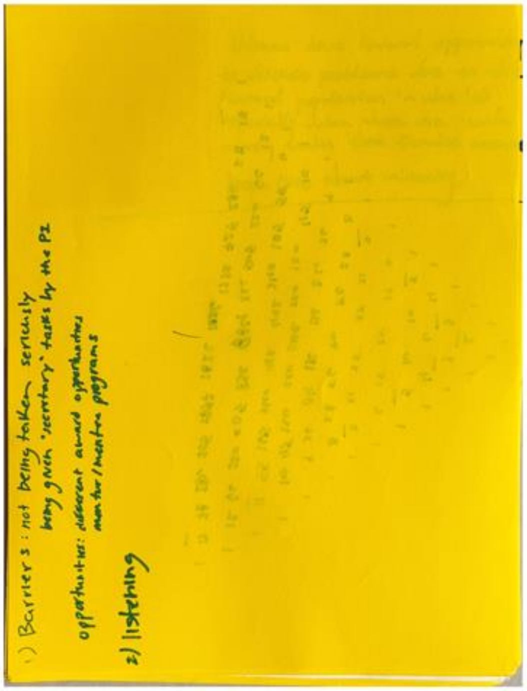
1) Barriers: not being taken seriously
being given "secretary" tasks by the PI
opportunities: different award opportunities
mentor/mentee programs
2) listening

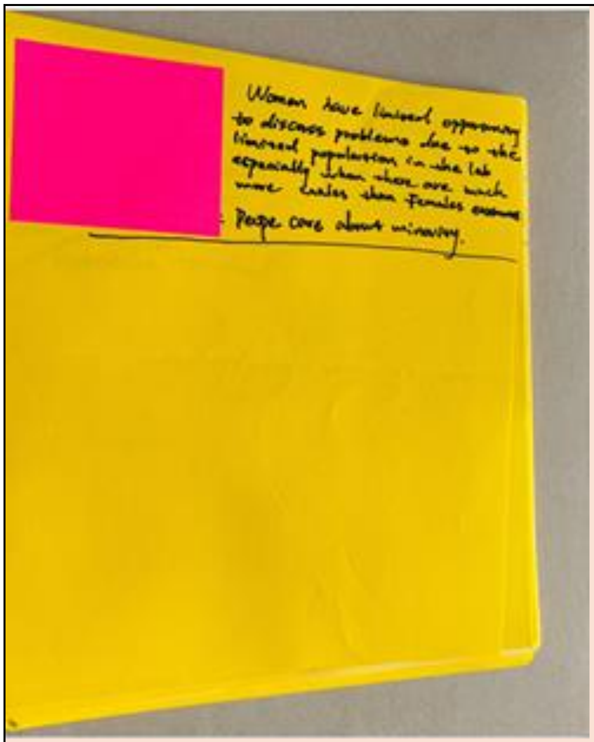
Women have limited opportunity to discuss problems due to the limited population in the lab especially when there are much more males than females
People care about minority

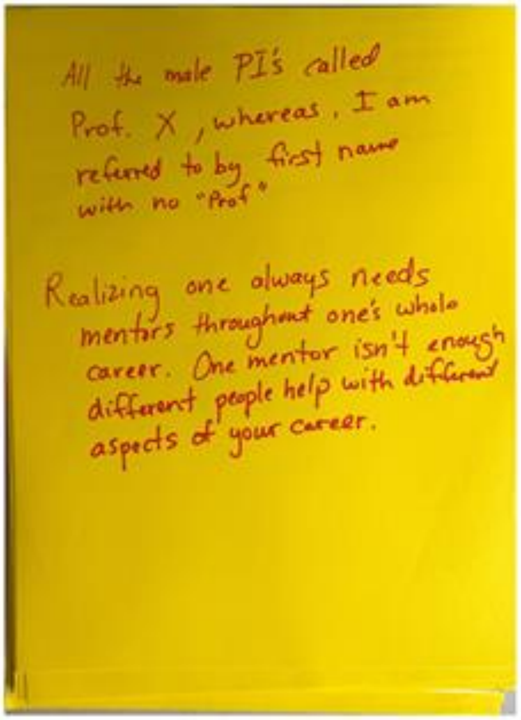
All the male PI's called Prof. X, whereas, I am referred to by first name with no "Prof"
Realizing one always needs mentors throughout one's whole career. One mentor isn't enough different people help with different aspects of your career.

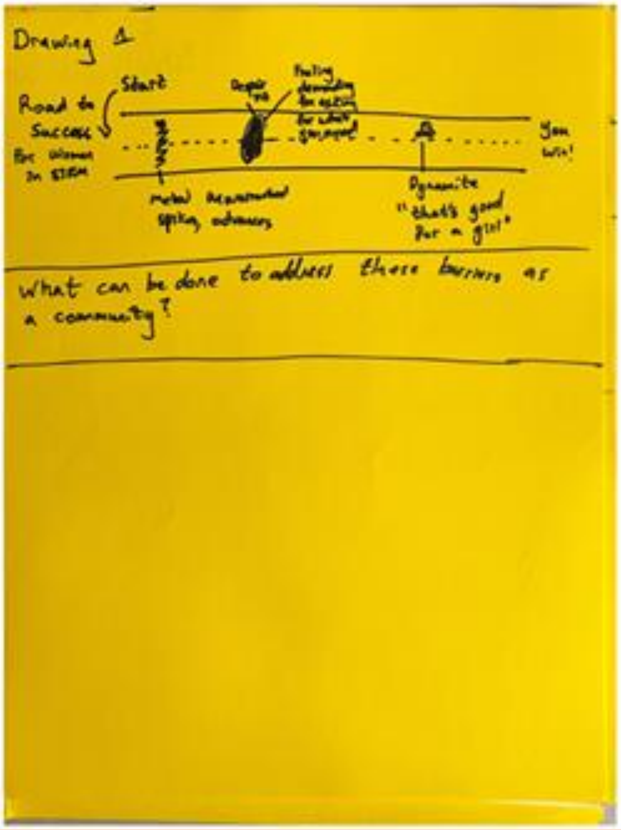
Drawing 1
Start
Road to Success for Women in STEM
Dynamite "that's good for a girl"
You win!
What can be done to address these barriers as a community?

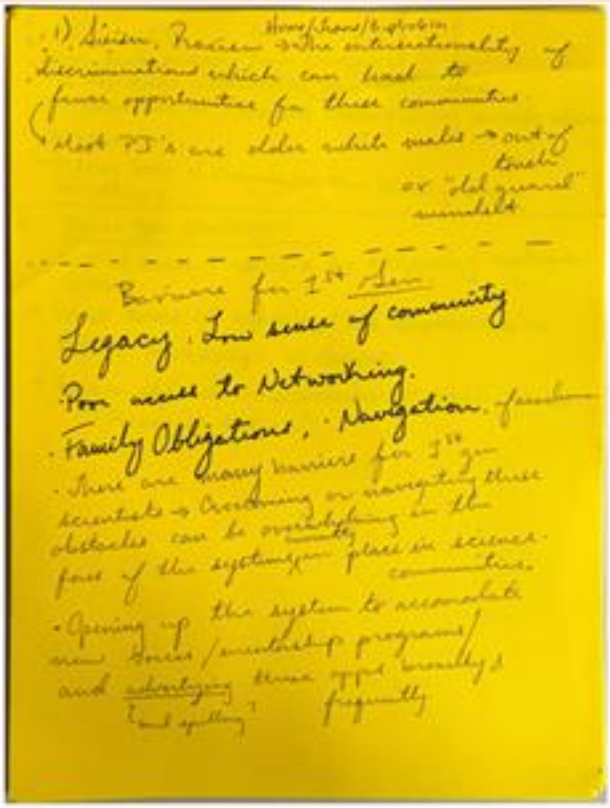
Legacy. Low sense of community
Poor access to Networking.
Family Obligations, Navigation.

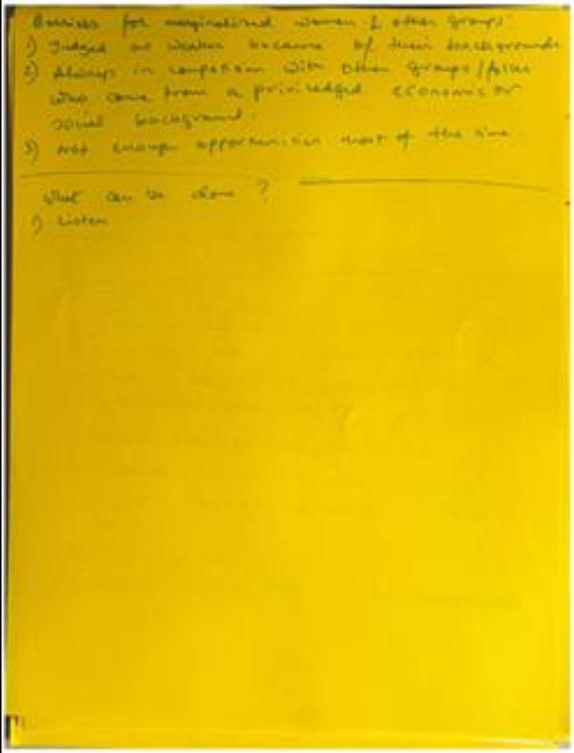
Barriers for marginalized women & other groups
What can be done?
1) Listen

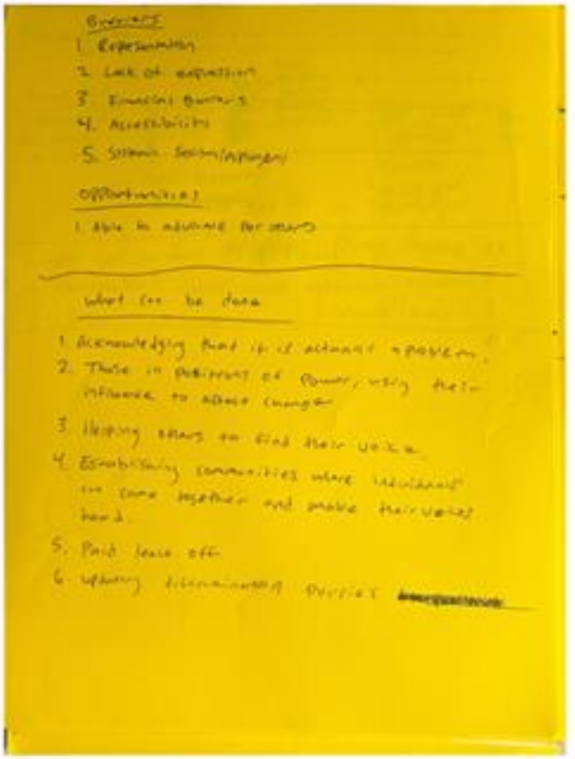
Barriers
1. Representation
3. Financial barriers
4. Accessibility
Opportunities
What can be done
1. Acknowledging that it is actually a problem
3. Helping others to find their voice
5. Paid leave off

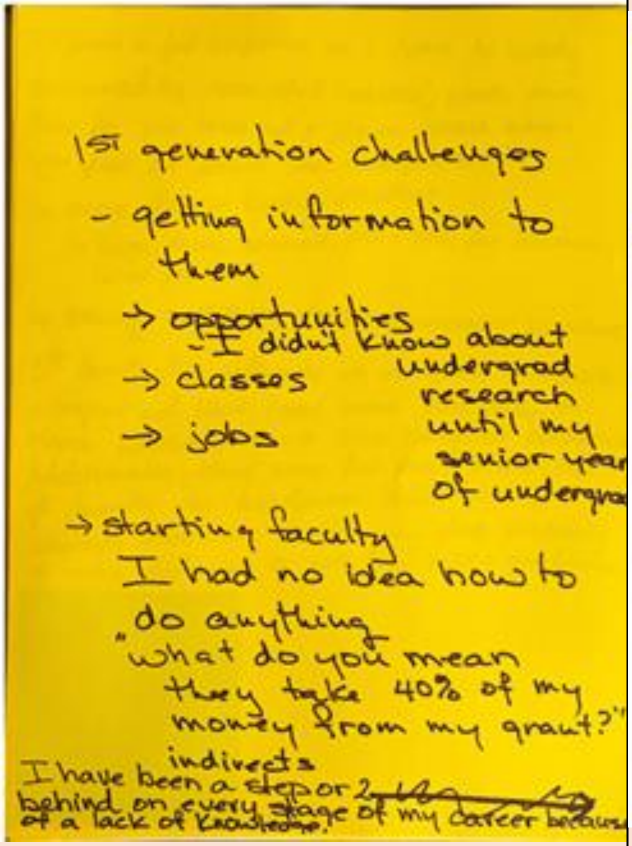
1st generation challenges
- getting information to them
→ opportunities
→ classes
→ jobs
I didn't know about undergrad research until my senior year of undergra
→ starting faculty
I had no idea how to do anything
"what do you mean they take 40% of my money from my grant?"
indirects
I have been a step or 2 behind on every stage of my career because of a lack of knowledge.

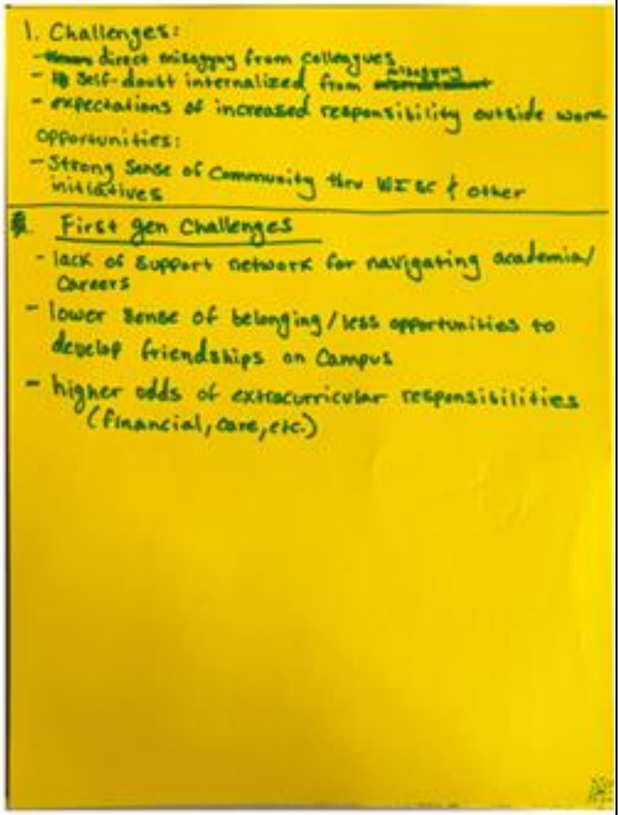
1. Challenges:
- direct misogyny from colleagues
- Self-doubt internalized from misogyny
- expectations of increased responsibility outside work
Opportunities:
- Strong sense of community thru & other initiatives
First gen Challenges
- lack of support network for navigating academia/careers
- lower sense of belonging / less opportunities to develop friendships on campus
- higher odds of extracurricular responsibilities (financial, care, etc.)

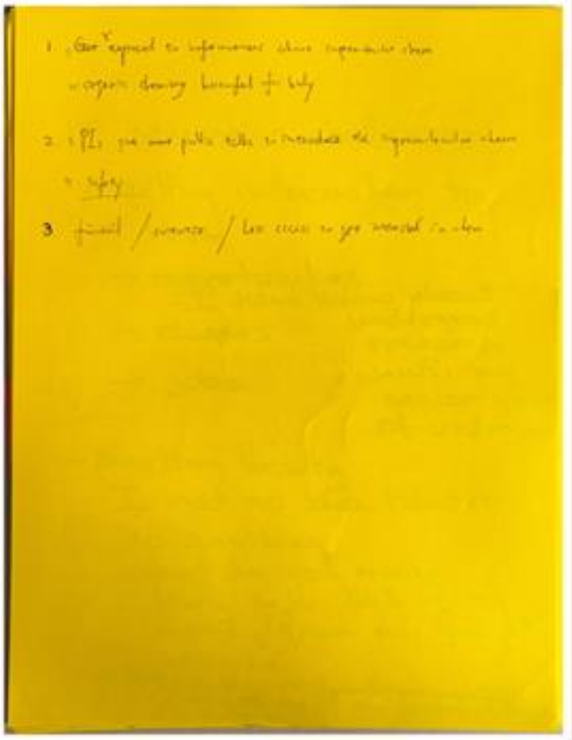

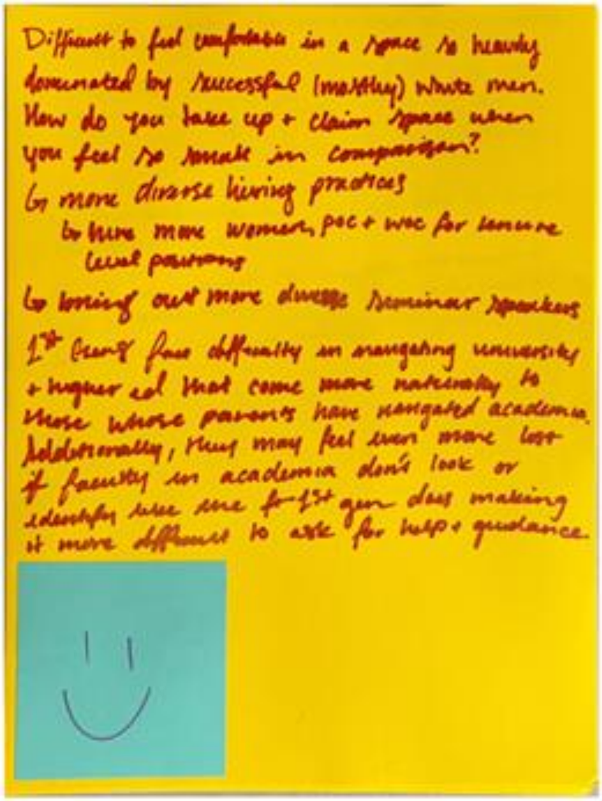
Difficult to feel comfortable in a space so heavily dominated by successful (mostly) white men.
How do you take up + claim space when you feel so small in comparison?
more diverse hiring practices
hire more women, poc + woc for tenure level positions
bring out more diverse seminar speakers
more difficult to ask for help + guidance

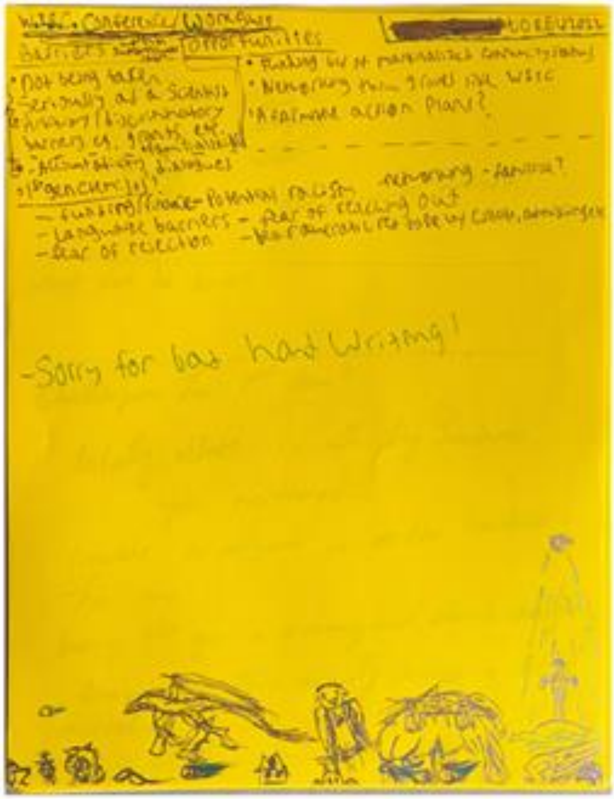

Barriers
Opportunities
- Language barriers - fear of reaching out
- fear of rejection
-Sorry for bad hand Writing!

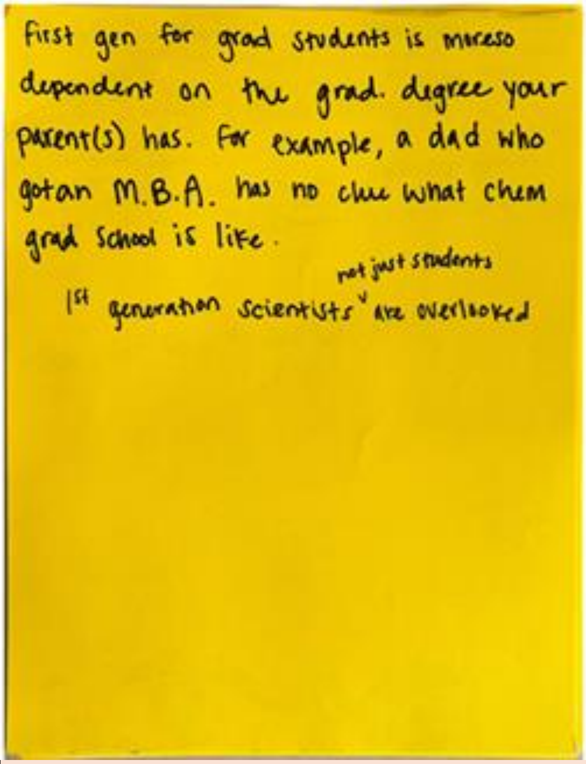

First gen for grad students is moreso dependent on the grad. degree your parent(s) has. For example, a dad who got an M.B.A. has no clue what chem grad school is like.
1st generation scientists not just students are overlooked

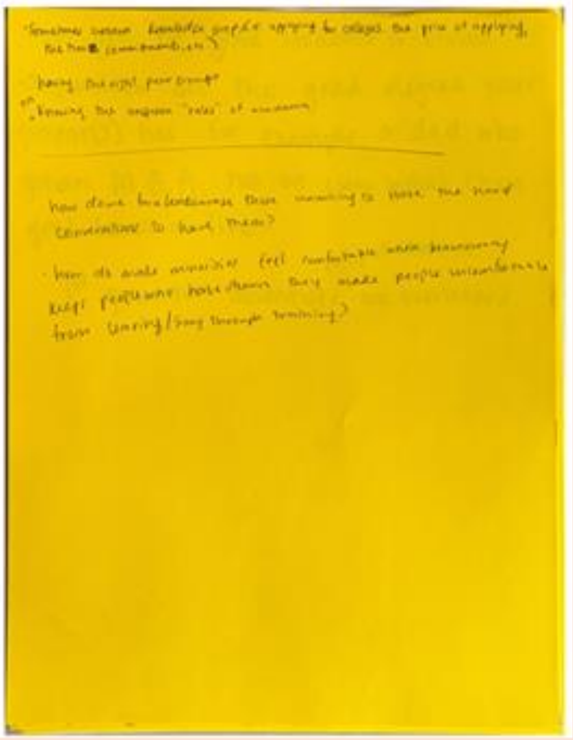

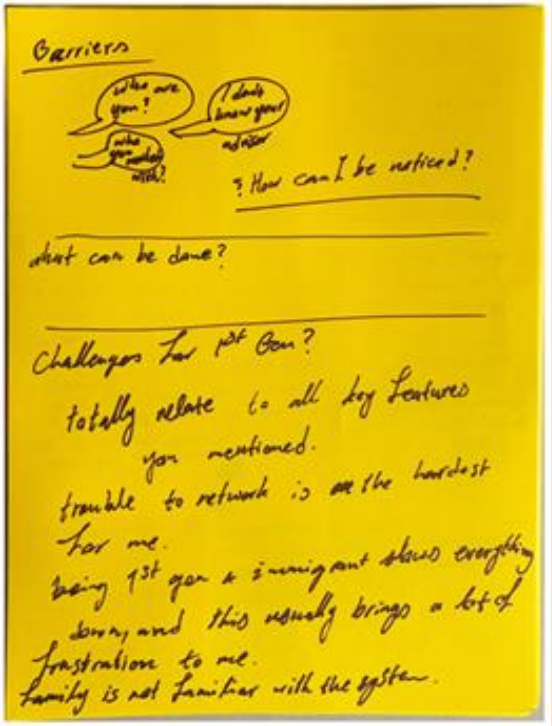

Barriers
who are you?
I don't know your advisor
who do you work with?
? How can I be noticed?
what can be done?
Challenges for 1st Gen?
totally relate to all key features you mentioned.
trouble to network is ~~as~~ the hardest for me.
being 1st gen & immigrant slows everything down, and this usually brings a lot of frustration to me.
family is not familiar with the ~~sys~~ system.

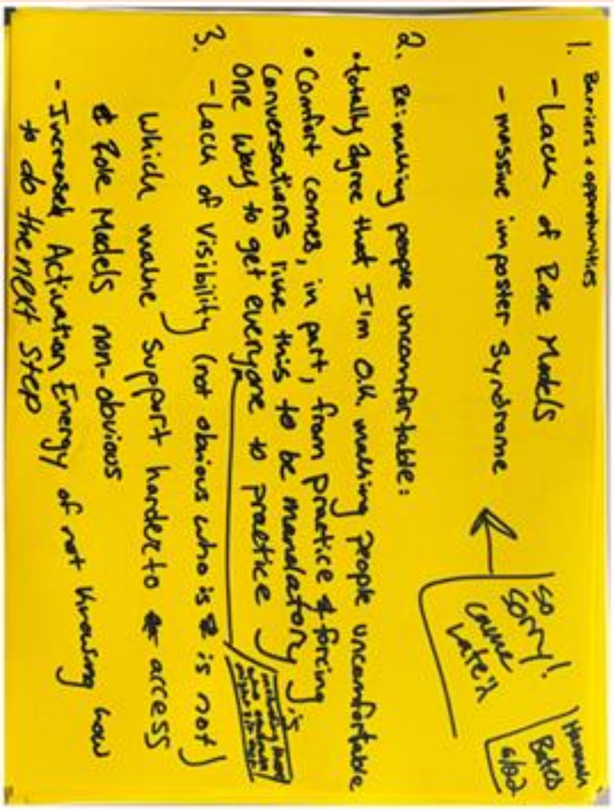
Barriers + opportunities
1. - Lack of Role Models
- massive imposter Syndrome
so sorry! came late!
2. Re: making people uncomfortable:
• totally agree that I'm O.K. making people uncomfortable
• Comfort comes, in part, from practice & forcing conversations like this to be mandatory is one way to get everyone to practice
3. - Lack of Visibility (not obvious who is & is not) which makes support harder to access & Role Models non-obvious
- Increased Activation Energy of not knowing how to do the next step
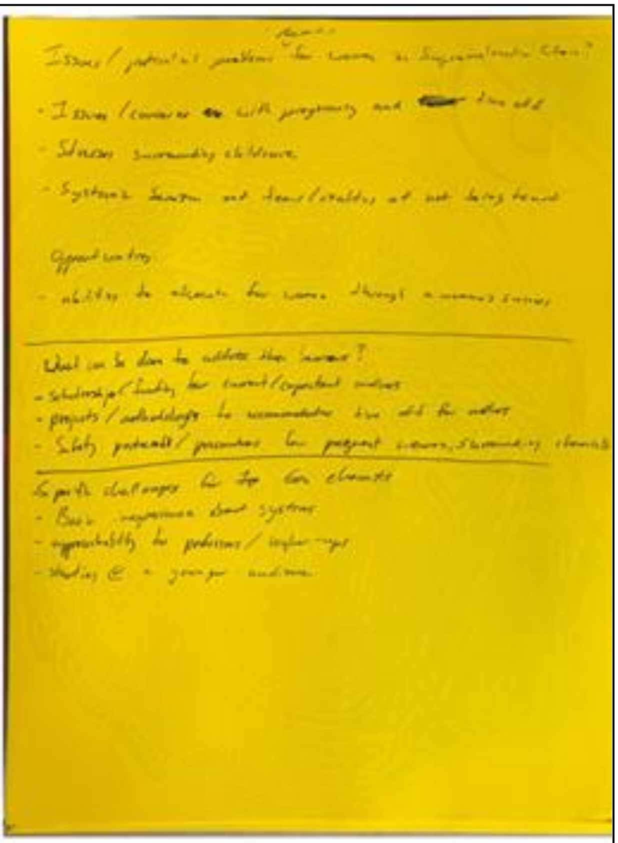

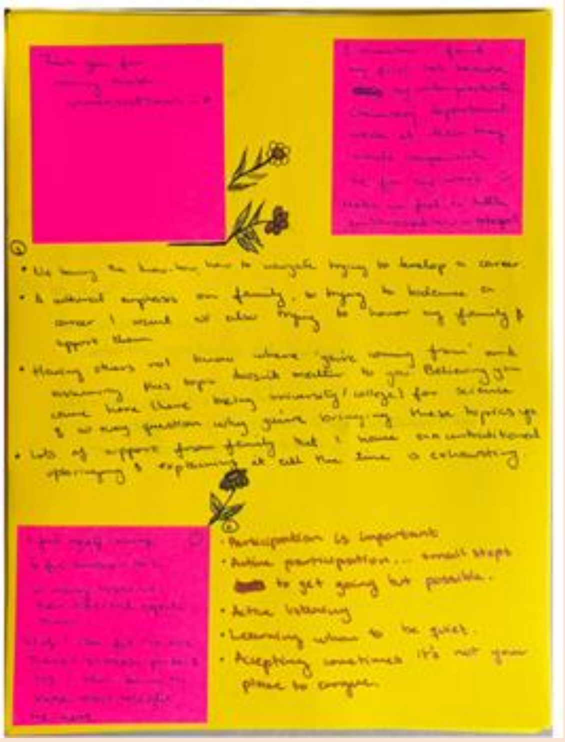

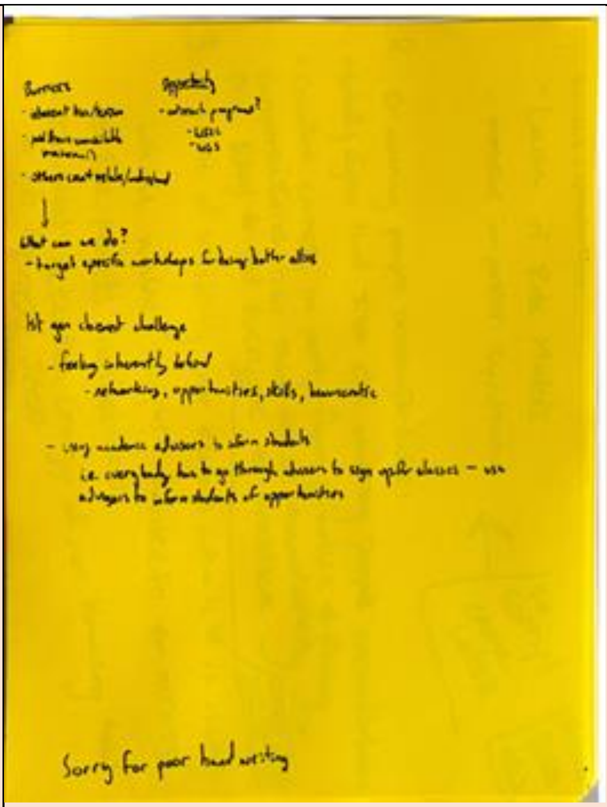
What can we do?
Sorry for poor handwriting

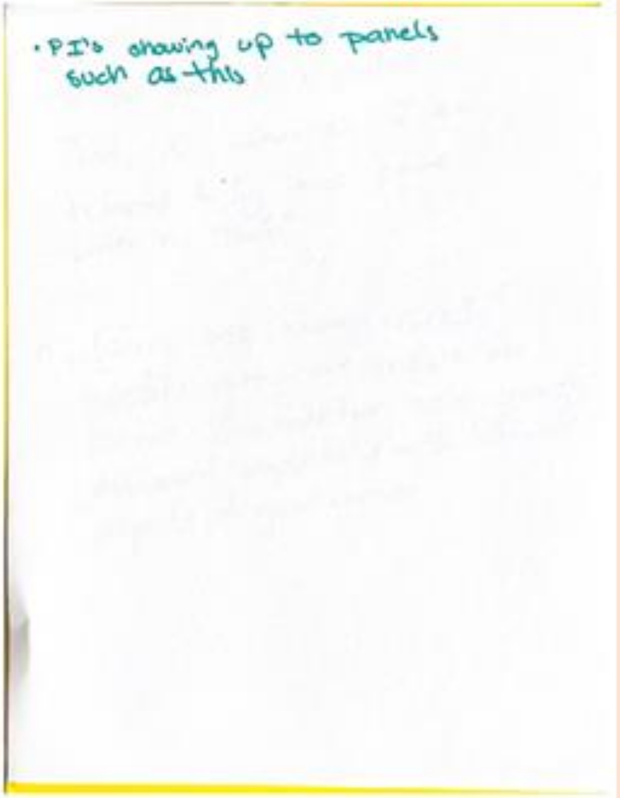
· PI's showing up to panels such as this

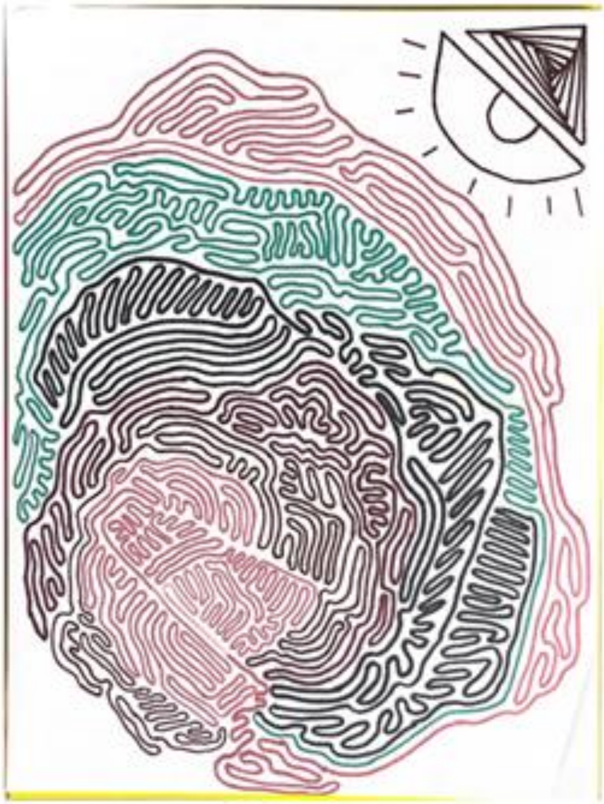

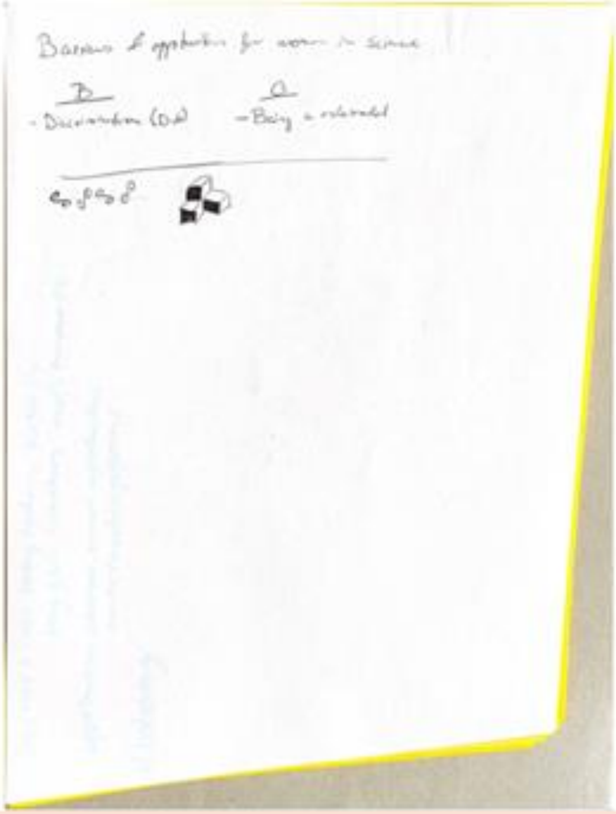

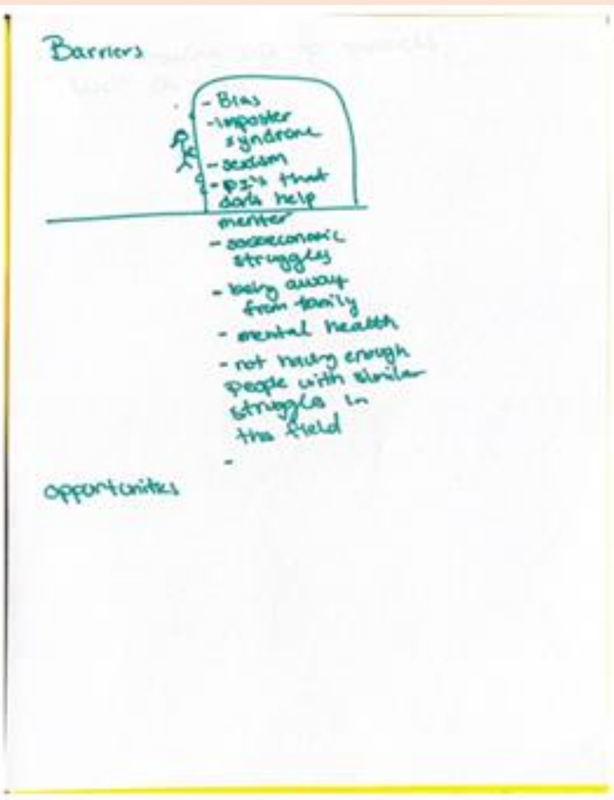
Barriers
- Bias
- imposter syndrome
- sexism
- PI's that don't help mentor
- socioeconomic struggles
- being away from family
- mental health
- not having enough people with similar struggles in the field
opportunities

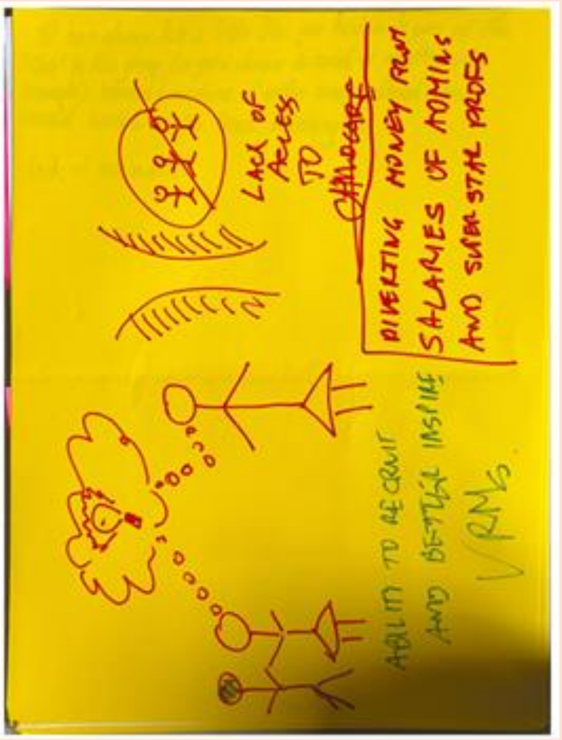
LACK OF ACCESS TO CHILDCARE
DIVERTING MONEY FROM SALARIES OF ADMINS AND SUPERSTAR PROFS

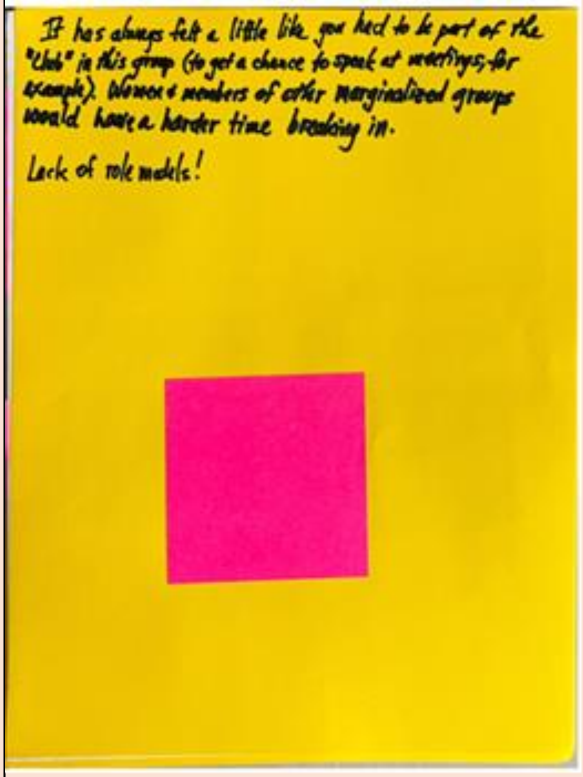
It has always felt a little like you had to be part of the "club" in this group (to get a chance to speak at meetings, for example). Women & members of other marginalized groups would have a harder time breaking in.
Lack of role models!

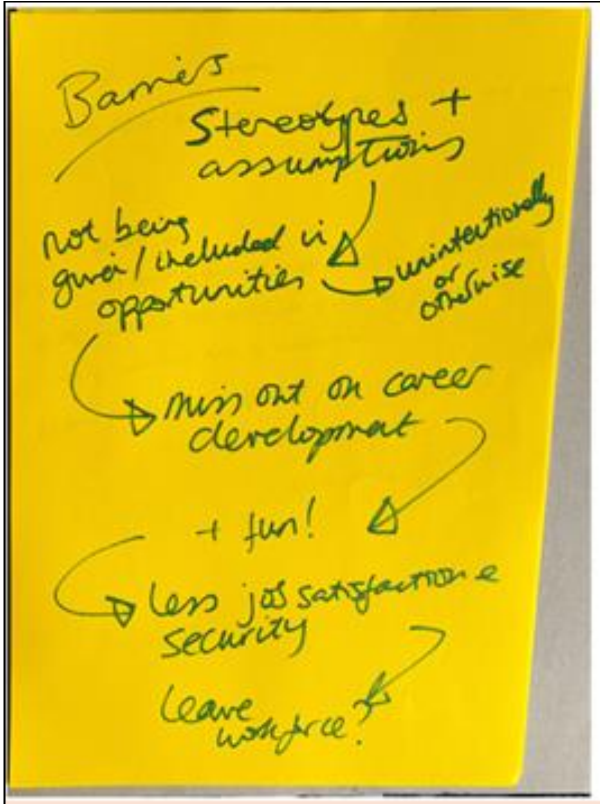
Barriers
Stereotypes + assumptions
not being given / included in opportunities
unintentionally or otherwise
miss out on career development
+ fun!
less job satisfaction + security
leave workforce?

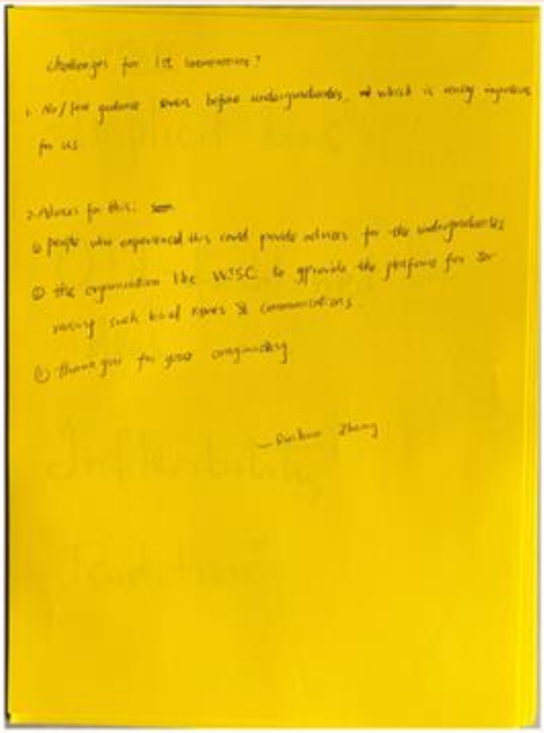

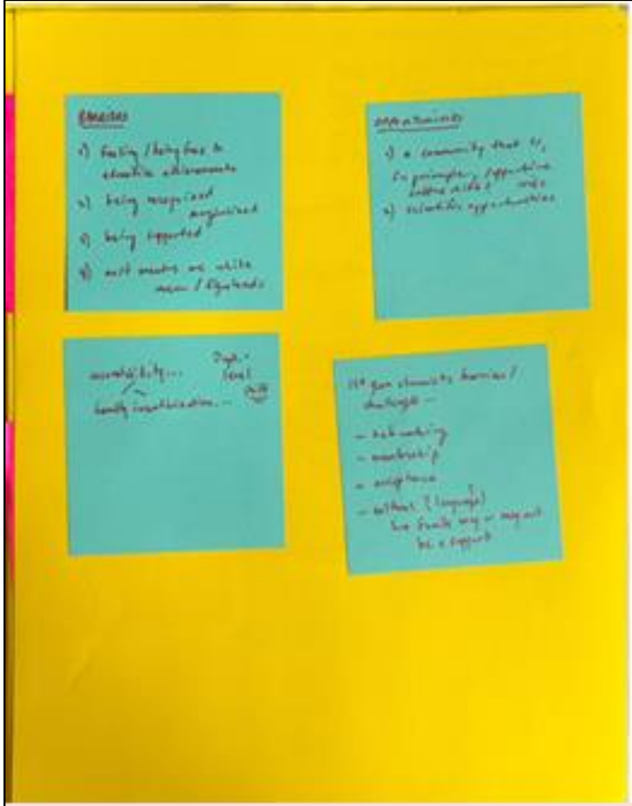

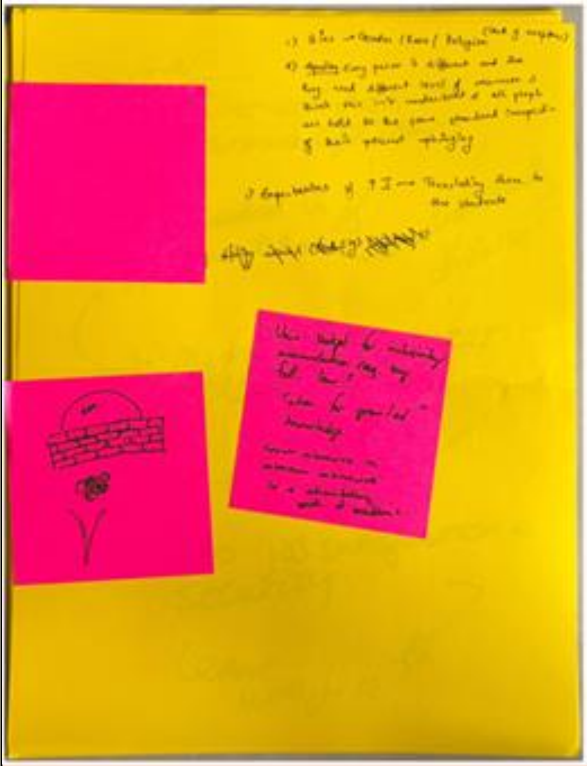

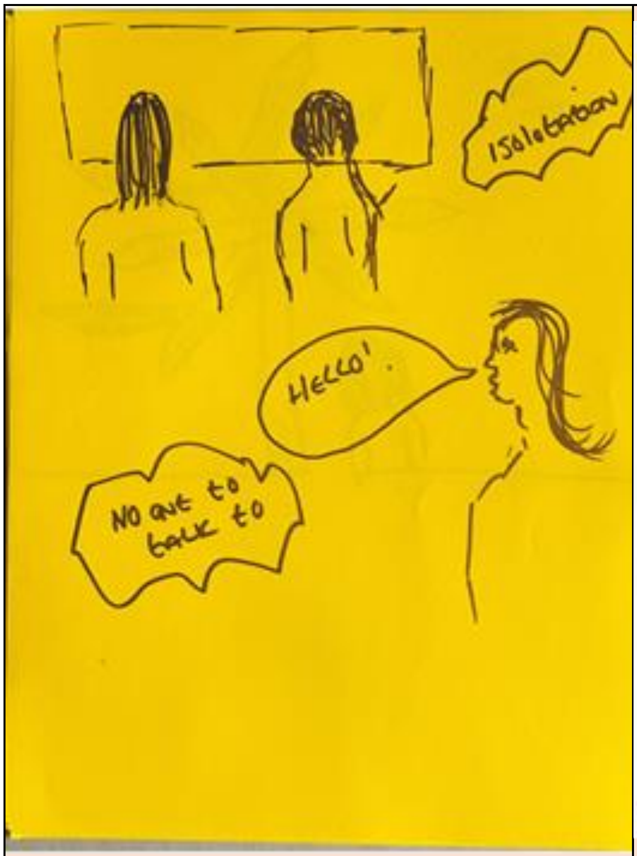
HELLO!
NO ONE to talk to

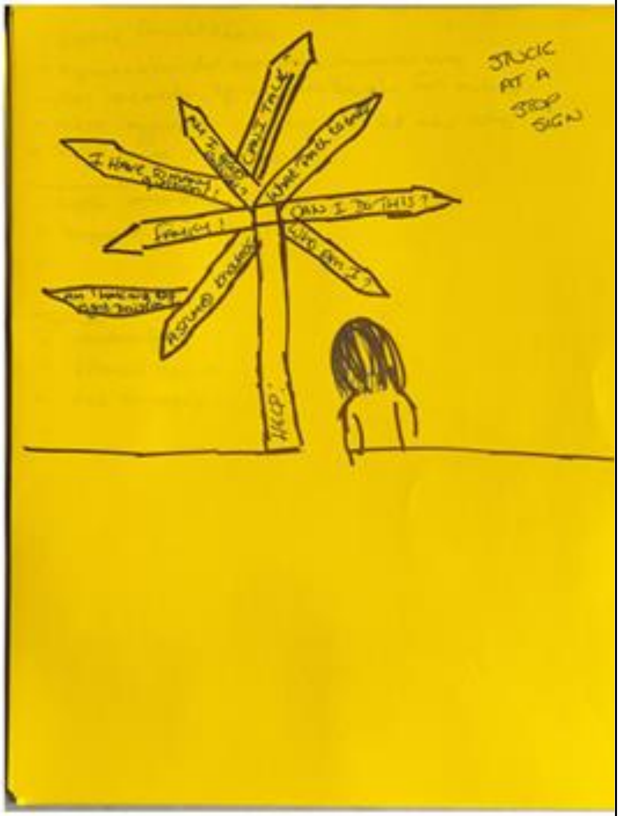
CAN I TALK?
CAN I DO THIS?
FAMILY!
WHO AM I?
HELP!
STUCK AT A STOP SIGN

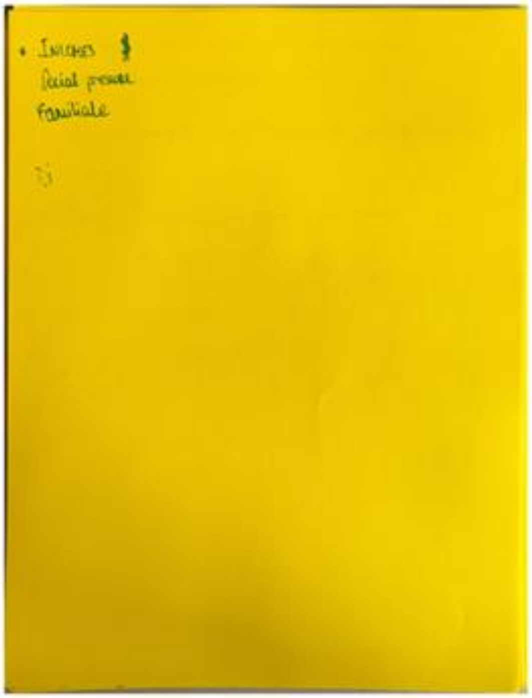

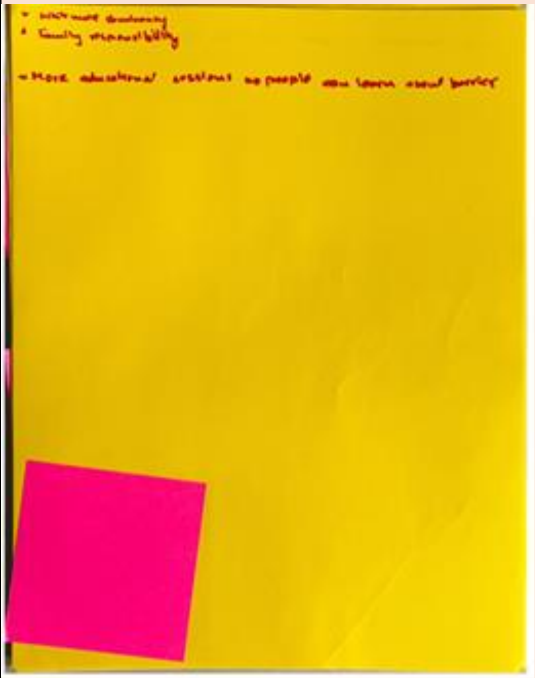

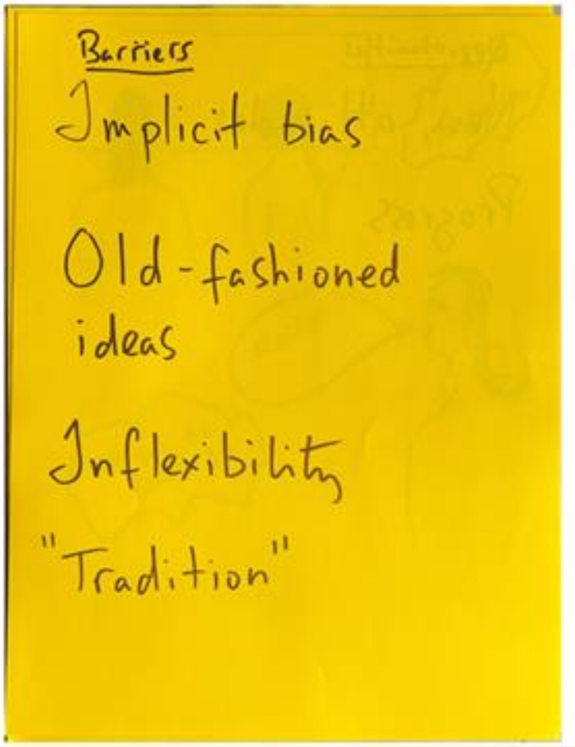
Barriers
Implicit bias
Old-fashioned ideas
Inflexibility
"Tradition"

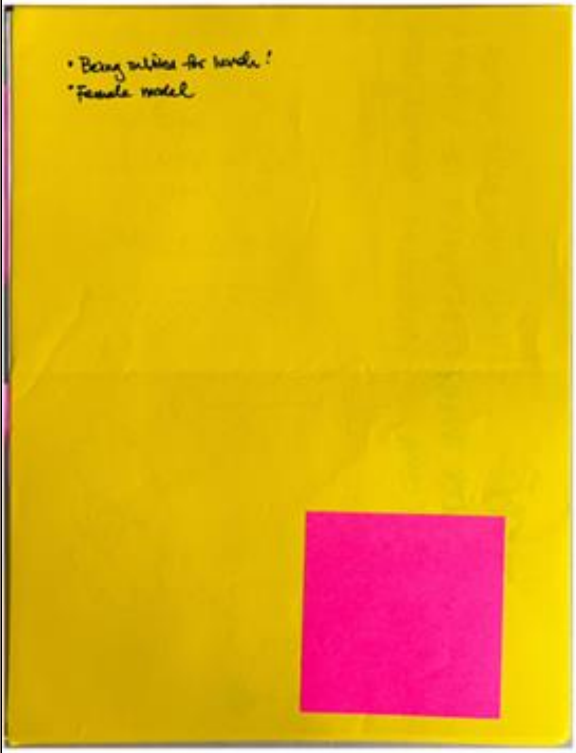

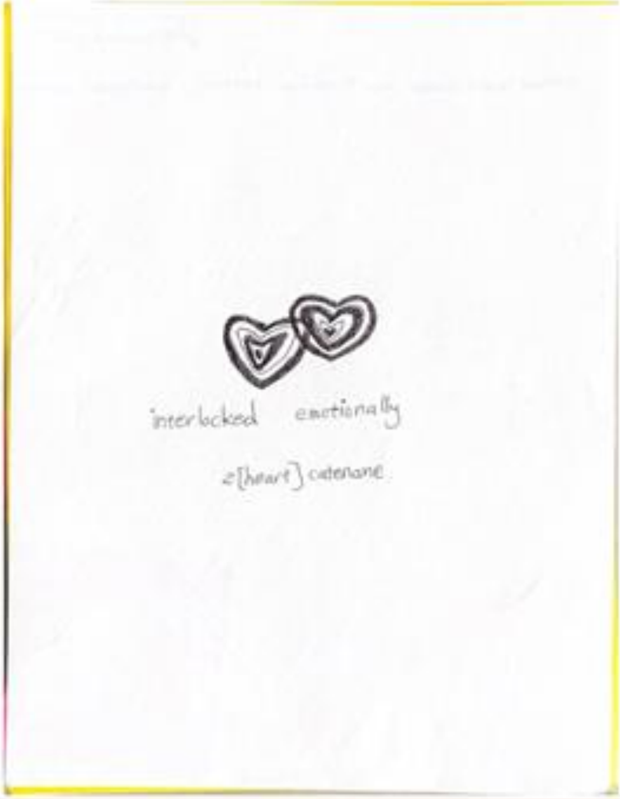
interlocked emotionally
z[heart] catenane

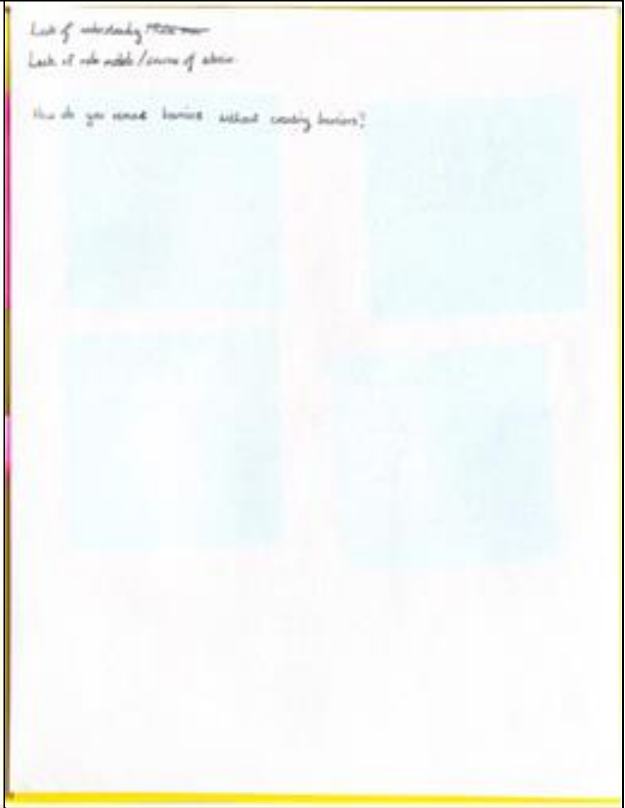

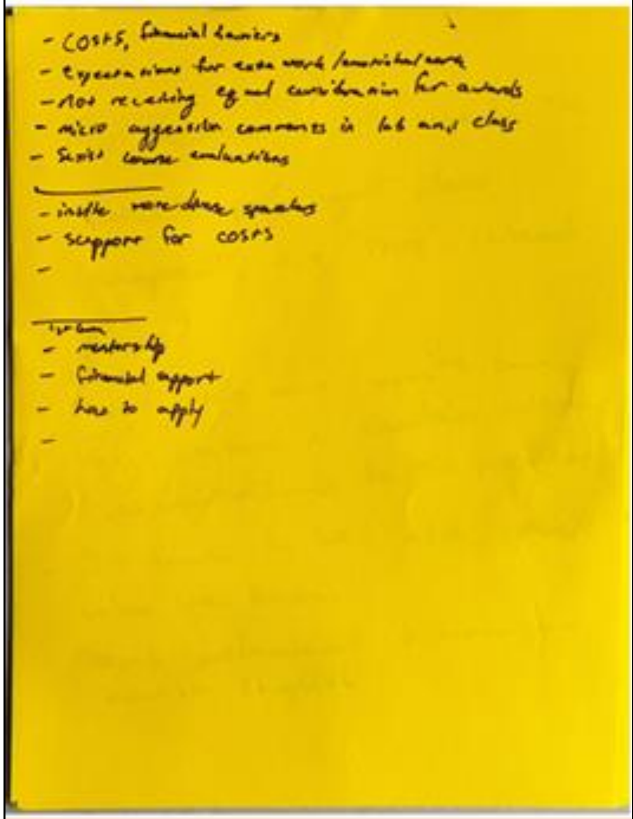
- costs, financial barriers
- micro aggression comments in lab and class
- Sexist course evaluations
- support for costs
- mentorship
- financial support
- how to apply

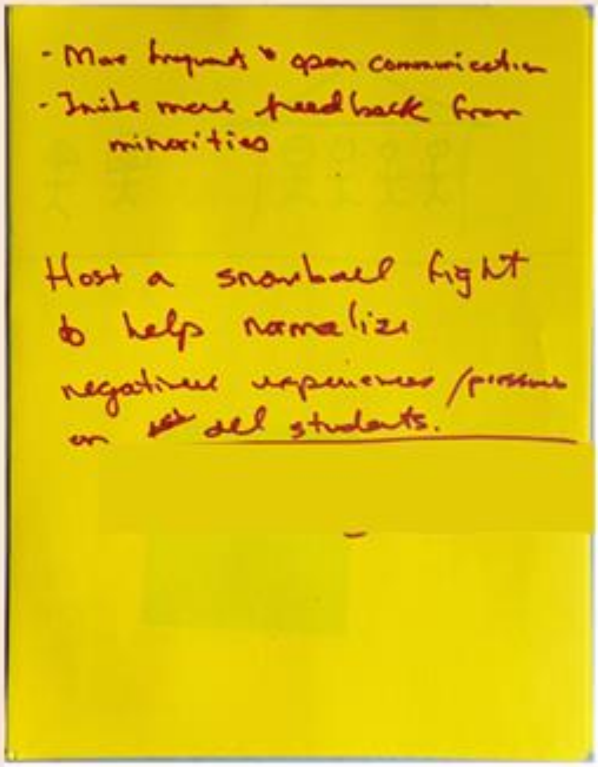
- More frequent + open communication
- Invite more feedback from minorities
Host a snowball fight to help normalize negative experiences / pressure on all students.

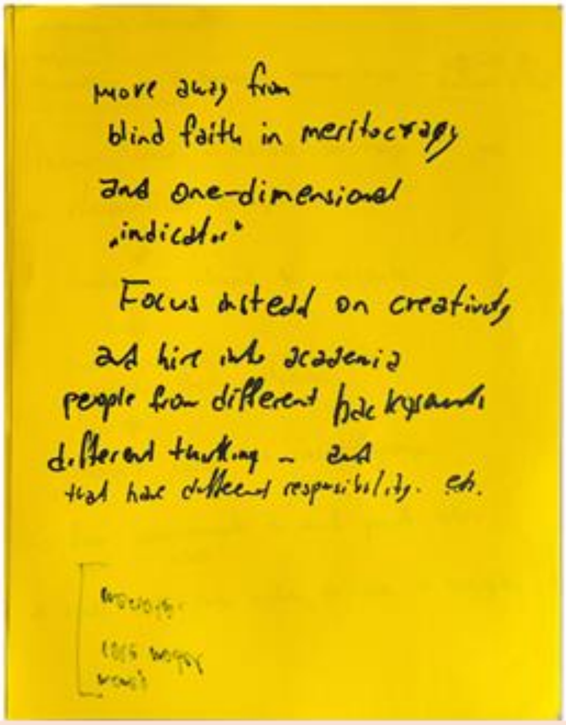
move away from blind faith in meritocracy and one-dimensional „indicator"
Focus instead on creativity and hire into academia people from different backgrounds different thinking – and that have different responsibility. etc.

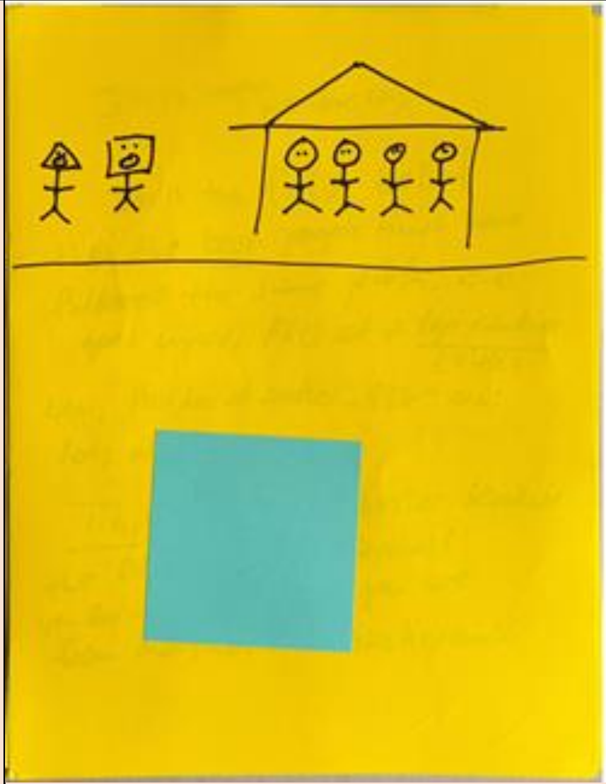

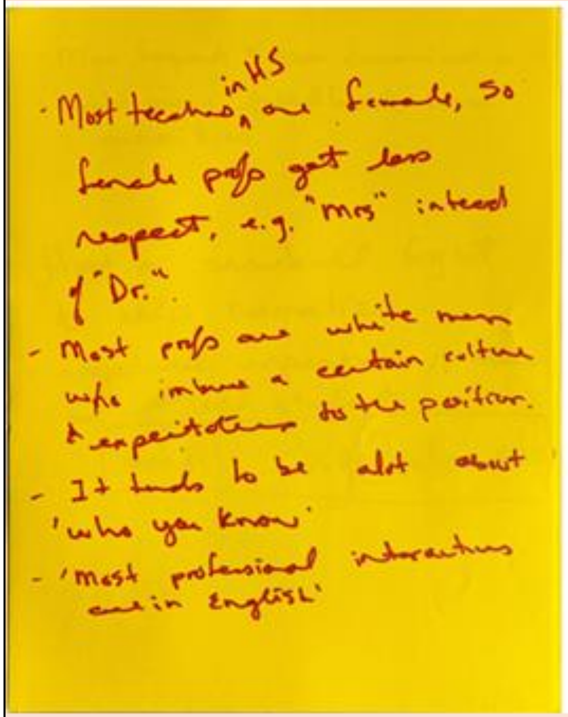
- Most teachers in HS are female, so female profs get less respect, e.g. "Mrs" instead of "Dr.".
- Most profs are white men who imbue a certain culture & expectations to the position.
- It tends to be alot about 'who you know'
- 'most professional interactions are in English'

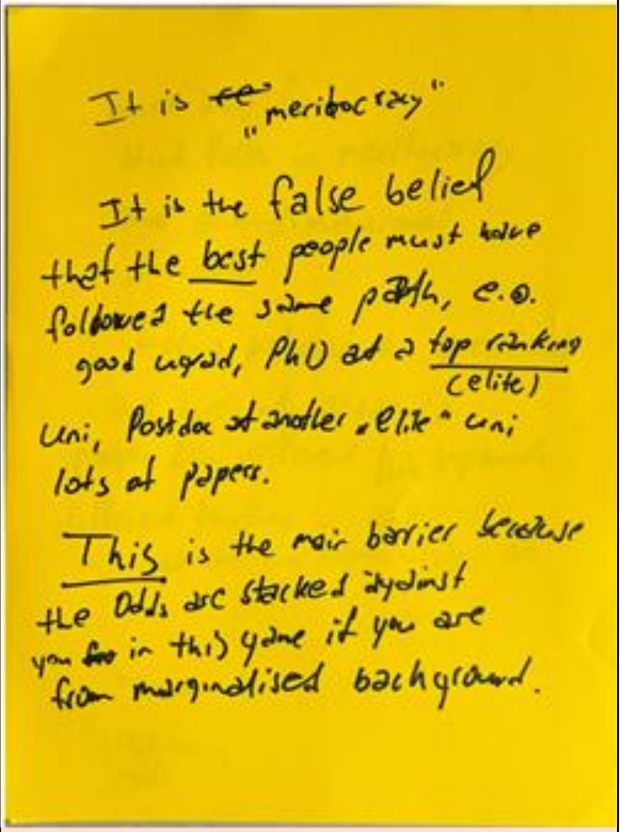
It is "meritocracy"
It is the false belief that the best people must have followed the same path, e.g. good ugrad, PhD at a top ranking (elite) uni, Postdoc at another "elite" uni lots of papers.
This is the main barrier because the odds are stacked against you in this game if you are from marginalised background.

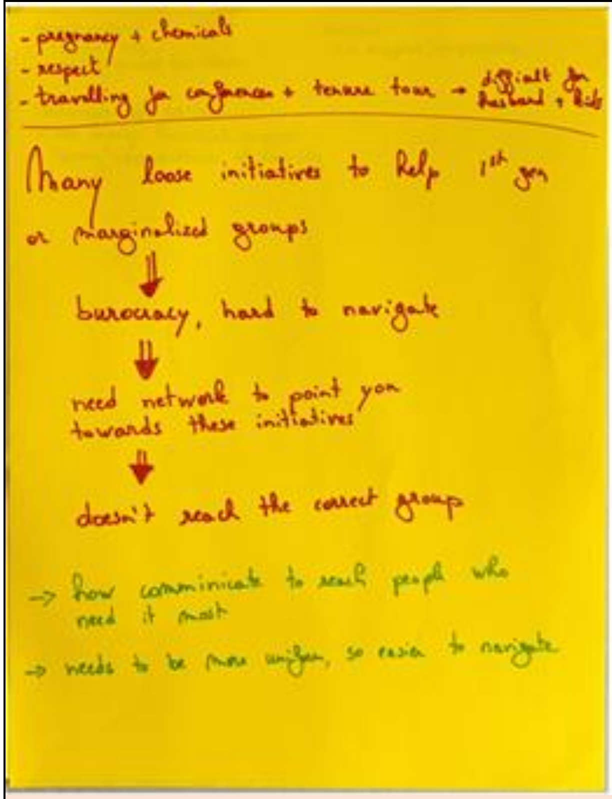
Many loose initiatives to help 1st gen or marginalized groups
burocracy, hard to navigate
need network to point you towards these initiatives
doesn't reach the correct group
-> how comminicate to reach people who need it most
-> needs to be more unifyed, so easier to navigate

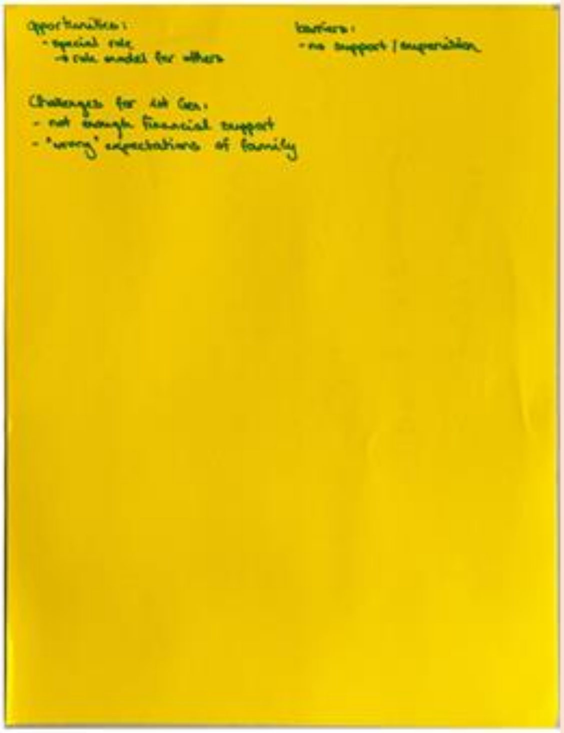
Opportunities:
- special role
-> role model for others
Barriers:
- no support / supervision
Challenges for 1st Gen.:
- not enough financial support
- "wrong" expectations of family

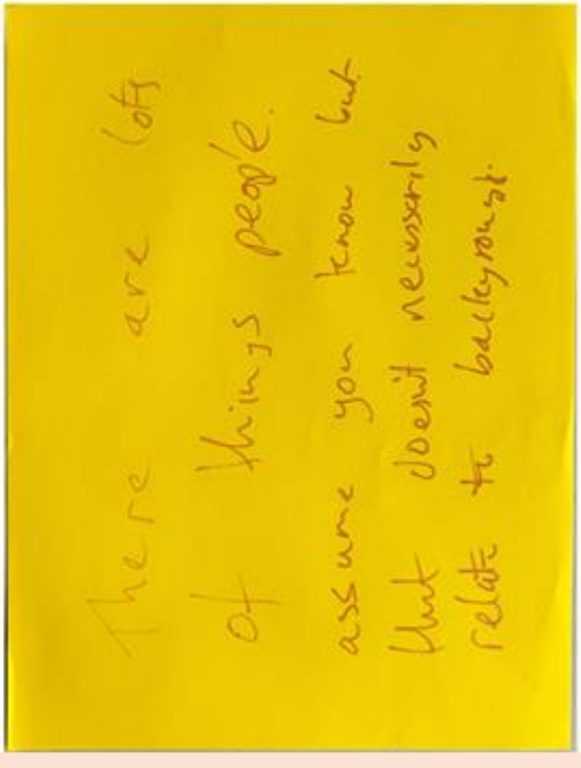
There are lots of things people assume you know but that doesn't necessarily relate to background.

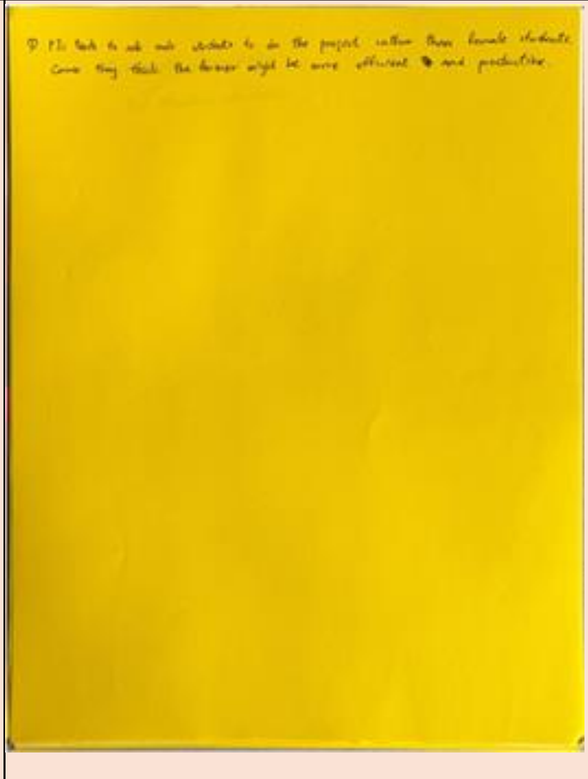

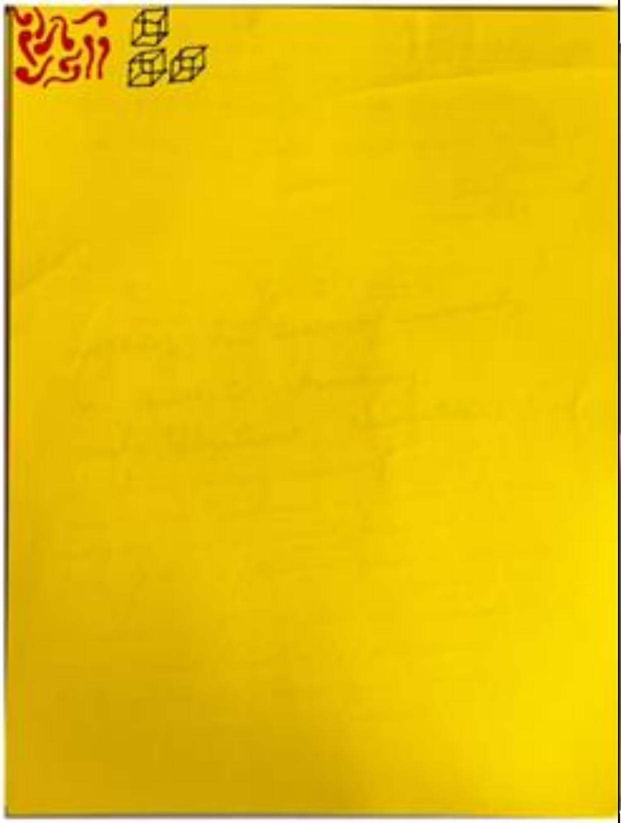

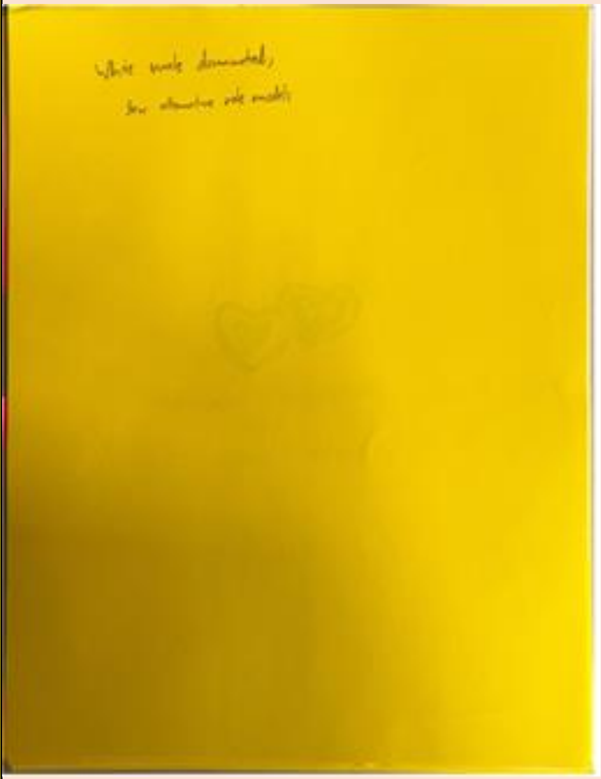
White male dominated,
few alternative role models

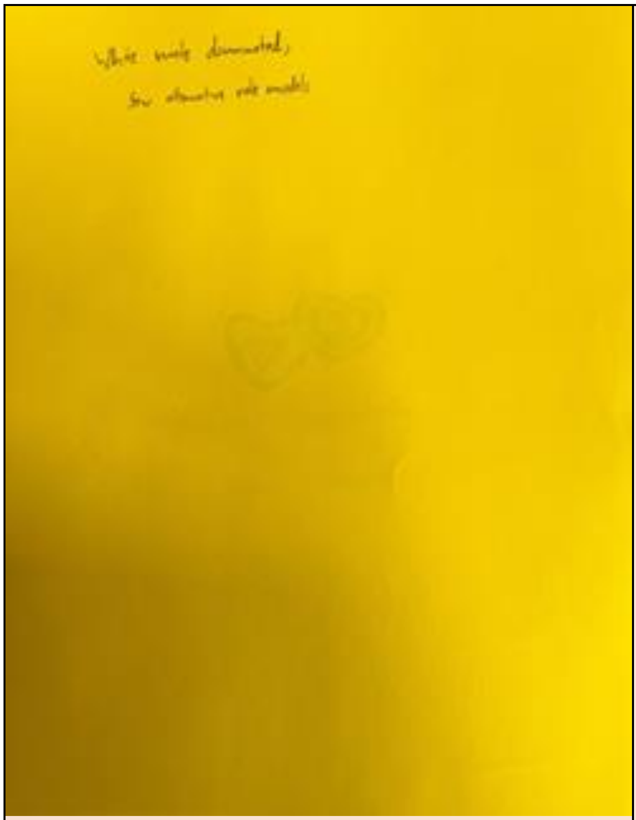

Fig. S3: Examples of data from ISMSC 2022

7.4 Online note board from CALIX 2022

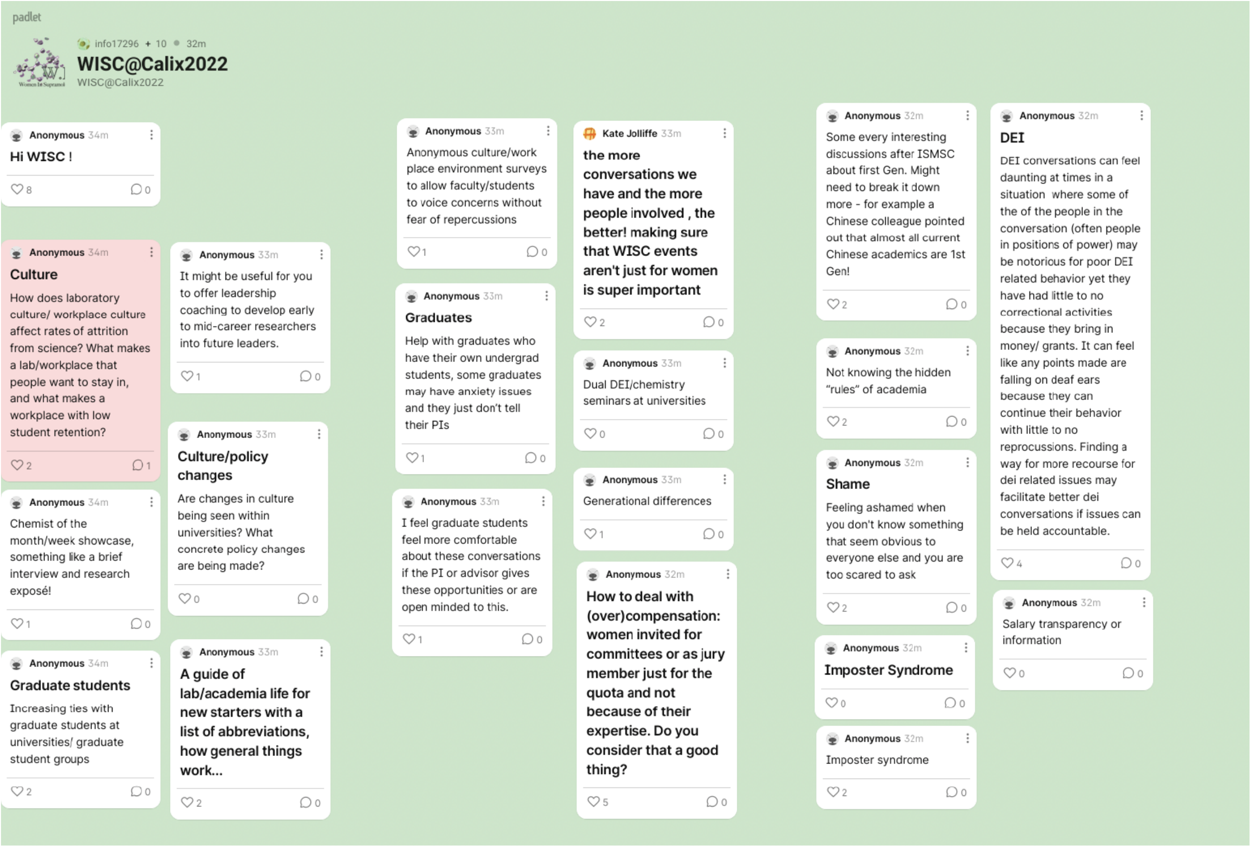

Fig. S4: Virtual note board from CALIX2022.

Section 8 Tables

Table S1: Categorized sources by main theme.

**Category**		**Number of sources**
1.	Included Texts	10
2.	Academia	54
3.	STEM	38
4.	Chemistry	7
5.	EDI	28
6.	Women	3
7.	Mentoring	7

Table S2: Summaries of 10 texts included in category one from the 158 found in the semi-systematic literature review.

Category 1		Notes
1.	Stockard, J., Rohlfing, C. M. & Richmond, G. L. Equity for women and underrepresented minorities in STEM: Graduate experiences and career plans in chemistry. *Proc Natl Acad Sci U S A* 118, (2021).	This study surveyed PhD students in chemistry departments in the US to explore the experiences of Black and minority students of graduate study. They found First Gen students were more likely to be from minority backgrounds. Their data also revealed women and women in unrepresented minorities were less satisfied with their supervisory relationships. Minority students were more than twice as likely to say their financial support was not adequate and women and minority students were less likely to be satisfied with their peer and post-doc support.
2.	Bancroft, S. F., Fowler, S. R., Jalaeian, M. & Patterson, K. Levelling the Field: Flipped Instruction as a Tool for Promoting Equity in General Chemistry. *J Chem Educ* 97, 36–47 (2020).	This study implemented a unique 'flipped course' design to measure student outcomes against a traditional 'General Chemistry 1' course in a college in the US. All students benefited in this new course mode, although students with low socioeconomic status appeared to benefit the least. The study observed increased attendance, more group problem solving and active learning in this flipped model. More positive performance was observed in completion of homework, exams and iClicker questions. Stressing that students from African American and Latin American background come from communities with historically less opportunity for economic growth, often are from low socio-economic backgrounds and first-generation college students, they emphasise the need for additional supports for these student groups. They state there is need for further investigation the out-of-class activities that burden student performance for those with low economic status. The first author is a Latin American First Gen and concludes that structural reform may be integral to better serving underserved student populations.
3.	Kennedy, S. A. *et al.* Faculty Professional Development on Inclusive Pedagogy Yields Chemistry Curriculum Transformation,	This study launched and measured a faculty professional development programme for faculty in Biology, Chemistry and Physics aimed to enhance the comprehension of these staff members of the marginalisation of particular student groups. Faculty were given various tasks such as analysing the retention rates of particular student groups within their courses. Faculty were taught various inclusion practices such as removing assumed knowledge in their communication with students and

	Equity Awareness, and Community. *J Chem Educ* 99, 291–300 (2022).	presenting information in various ways. The study found that following this programme faculty were more sensitive to the realities of minoritized student groups such as First Gens.
4.	Gangitano, G. College Transition : Voices of First-Generation Minority STEM Students. *Theses, Dissertations and Culminating Projects* (2021).	This thesis explores the experiences of First Gens who are also people of colour from low-income households in their first year of University. The thesis highlights a greater need to specialised support for this student group, the importance of peer support and effective mentorship in the initiation process. The findings also highlight the resilience and adaptability of First Gens of colour from low income households.
5.	Goonewardene, A., Offutt, C., Whitling, J. & Woodhouse, D. An Interdisciplinary Approach to Success for Underrepresented Students in STEM. *J Coll Sci Teach* 045, (2016).	This study examines the “Nano Scholar’s Program”, an interdisciplinary science programme for underrepresented undergraduate students, including First Gens, where they were taught in small cohorts and were offered specialised academic and social support. The authors emphasis that successful support for unrepresented students must include academic as well as social support, financial aid and smaller cohort sizes fostering a supporting learning community amongst the students.
6.	Macphee, D., Farro, S. & Canetto, S. S. Academic Self-Efficacy and Performance of Underrepresented STEM Majors: Gender, Ethnic, and Social Class Patterns. *Analyses of Social Issues and Public Policy* 13, 347–369 (2013).	This study explored the concept of self-efficacy in in undergraduate students from various STEM disciplines including chemistry. 80% of their sample were First Gens. This study recruited students from a STEM mentorship scheme and discovered that students with double-disadvantage versus their single-disadvantage (meaning they were disadvantaged only by one characteristic e.g. being First Gen) counterparts benefited more from the scheme itself but scored lower on every measure of academic performance. The study found that although women perceived themselves to be academically weaker at admission, by graduation the levels of self-efficacy between men and women were equal.
7.	Voigt, M., Hagman, J.E., Gehrtz, J., Ratliff, B., Alexander, N.N., & Levy, R.A. Justice through the lens of calculus: Framing new possibilities	This volume explores various equity, diversity and inclusion issues within Universities delivery introductory mathematics courses on a range of STEM degrees including chemistry. Several of the included studies measured the performance of student’s dependant on their First Gen status and other marginalised characteristics. Some case studies introduced learning assistants, active learning and low stake assessments as ways to lower barriers to success for all students and increase academic

	for diversity, equity, and inclusion. (2021).	capital. The volume emphasises the need for faculty and institution support in developing capital and sense of belonging in underrepresented student groups.
8.	Uche, A. *The Retention Of First-Generation College Students In Stem: An Extension Of Tinto's Longitudinal Model*. *Unc Charlotte Electronic Theses And Dissertations*. (2015).	The author focuses on the experiences of underrepresented students in gateway STEM courses, (computer science, engineering and chemistry) during their initiation to University. Half of the students included from these STEM majors were First Gen. The findings indicate that pre-University experiences and effective teaching in Maths and Science is key for First Gens pursuing STEM majors. The study also found that the pre reported 'chilly campus climate' for women remains prevalent in some STEM lab environments.
9.	Shedlosky-Shoemaker, R. & Fautch, J. M. Who leaves, who stays? Psychological predictors of undergraduate chemistry students' persistence. *J Chem Educ* 92, 408–414 (2015).	This study explores various reasons for persistence, switching or leaving in Chemistry majors. The study took into a account various characteristics in students such as First Gen status and ethnicity. The study states the First Gen status is not unrelated to persistence in their sample but is not the main focus of the study. Instead, authors highlight findings related to individual differences such as self-doubt and academic performance to be indicators of persistence. The study found, for example, that self-worth of persisters was less likely to be affected by competition or outcomes relevant to academic competence.
10.	Snodgrass Rangel, V., Vaval, L. & Bowers, A. Investigating underrepresented and first-generation college students' science and math motivational beliefs: A nationally representative study using latent profile analysis. *Sci Educ* 104, 1041–1070 (2020).	The study concerns high school students and their achievements and perception of maths and science including chemistry. They specifically concern students who took a third year of maths and science in high school, 47% of their sample for First Gens. The study found evidence of negative beliefs around maths and science for a portion of the First Gens however they academically performed comparably to their continuing generation counterparts at this stage.

Table S3: Codebook from Study 1. ***Child codes indicated in italics.***

**Code name**	**Code Description**
First Gen in academia	Concerning experiences or outcomes relating to First Gen in higher education.
First Gen in STEM	Concerning experiences or outcomes relating to First Generation STEM students in higher education.
Academic and institutional support	Concerning supports from universities extended to First Gen.
Mentoring	
Need of 1st gen project	
Role of supervision	
Barriers 1st gen face	Concerning negative education experiences associated with First Gen status.
Financial resources	
Belonging	
Social class	
Clothes	
Community	Concerning reference to social and academic communities.
Competition	Concerning reference to competitive pressures within academia.
Coping strategies	Concerning methods used by individuals to overcome the barriers they are experiencing within higher education/academia.
COVID--19	Concerning reference to the COVID-19 pandemic and its impact within higher education/academia.
Creativity	Concerning creative, original and/or artistic ideas and work of individuals associated with First Gen status.
Cultural and social capital	Concerning reference to types of information, privileges, and resources that could be described as cultural and social capital as described by Bourdieu [15]
Habitus	
Influence of family on career	
Residential mobility	
Discrimination	Concerning reference to behaviours in higher education/academic settings defined as discriminative based on protected characteristics.
Feminism	Concerning refence to acts, attitudes or beliefs associated with feminism.
First Gen term	Concerning the acronym 'First Gen' to refer to first-generation students or scholars.
Field specific	Concerning studies that were field/discipline specific in investigating the experiences of students and scholars in higher education/academia.

Harassment	Concerning reference to harassment of individuals in higher education/academic settings.
History of women in science	Concerning literature and reference to the historic exclusion of women in scientific research and work.
Identity	Concerning reference to personal and work identity of individuals working and/or studying in higher education.
Intersectionality	Concerning studies investigating the intersecting impacts of protected characteristics compiling in an individual affecting their experience of higher education/academia.
Racism	
Underrepresentation of Women in STEM	
URM	Underrepresented minorities
Imposter syndrome	Concerning reference to individuals' experiences of imposter syndrome and related terms such as 'imposter experience' and 'imposter phenomena' within higher education/academia.
Loneliness/isolation	Concerning reference to individuals' feelings of loneliness and isolation within higher education/academia.
Mental health	Concerning mental health and wellbeing of researchers and students in relation to being marginalised and otherwise.
Mentoring	Concerning reference to the role of mentoring within higher education/academia.
Motherhood	Concerning reference to the experiences of motherhood for students and researchers.
Organisations	Concerning reference to independent organisation separate to higher education institutions that do work in support of research and researchers and their roles in supporting marginalised scholars and First Gen.
Overwork	Concerning the experiences and demand for overwork within higher education/academia.
Pressure	Concerning reference to pressure applied to and experiences by individuals in higher education/academia.
Queen bees	Concerning reference to the concept of a 'Queen bee' within higher education institutions.
Research methods	Concerning reference to various kinds of research methods used in studies concerning the experiences of First Gen and other marginalised groups within higher education/academia.
Retention	Concerning the retention of marginalised researchers and students within higher education/academia.
Science interest	Concerning the levels of science interest in students in primary, secondary and post-secondary education prior to pursuing higher education.

Self-care	Concerning reference to various self-care practices used by marginalised students and researchers and First Gen in higher education/academia.
Statistics and numbers	Concerning the significant statistics that have been produced and published concerning the experiences of marginalised students and researchers in higher education and academia.
The body and embodiment	Concerning references to bodily experiences and embodiment practices used in understanding others' and one's own experiences within higher education/academia.
Verbal microaggressions	Concerning individuals' experiences of verbal microaggressions within higher education/academia.

Table S4: Full list of sources found in Study 1 by category.

**Category 2: Academia**
Bassett, B. S. Big enough to bother them? When low income, first-generation students seek help from support programs. *J Coll Stud Dev* 62, 19–36 (2021).
Beattie, I. R. Sociological Perspectives on First-Generation College Students. *Handbooks of Sociology and Social Research* 171–191 (2018).
Becerra, M. Mental Health and Academic Performance of First-Generation College Students and Continuing-Generation College Students. *UC Merced: Library*. (2017). Retrieved from https://escholarship.org/uc/item/4691k02z.
Bettencourt, G. M., Mansour, K. E., Hedayet, M., Feraud-King, P. T., Stephens, K. J., Tejada, M. M., & Kimball, E. Is First-Gen an Identity? How First-Generation College Students Make Meaning of Institutional and Familial Constructions of Self. *Journal of College Student Retention: Research, Theory & Practice*, *24*(2), 271-289 (2022).
Billings, K. R., & Young, K. M. How Cultural Capital Shapes Mental Health Care Seeking in College. Sociological Perspectives, 65(4), 637-660 (2022).
Bui, K. V. T. First-generation college students at a four-year university: background characteristics, reasons for pursuing higher education, and first-year experiences. *College Student Journal.*, *36*(1), 3–11 (2002).
Capriccioso (2006) - Aiding First-Generation Students NO LONGER AVAILABLE
Carnevale, A. P. & Smith, N. Balancing Work and Learning: Implications for<br><br>Low-Income Students. (2018).
Chemers, M. M., Hu, L.-t., & Garcia, B. F. Academic self-efficacy and first year college student performance and adjustment. *Journal of Educational Psychology, 93*(1), 55–64 (2001).
Costello, M., Ballin, A., Diamond, M. R. & Gao, L. First generation college students and non-first-generation college students: Perceptions of belonging. *J Nurs Educ Pract* 8, 58 (2018).
Dominguez-Whitehead, Y., Phommasa, M. & Caudillo, A. Unmasking First-Generation College Students and Professionals. *Journal of First-generation Student Success* 1, 145–155 (2021).
Engle et. al. Moving Beyond Access. College Success for Low-Income, First-Generation Students. (2008). Retrieved from www.pellinstitute.org.

Froggé, G. M. & Woods, K. H. Characteristics and Tendencies of First and Second-Generation University Students. The College Quarterly, *21(2).* (2018).

Gard Gardner, S. K. & Holley, K. A. 'Those invisible barriers are real': The

Progression of First-Generation Students Through Doctoral Education. *Equity and*

Excellence in Education 44, 77–92 (2011).

Gibbons, M. M. & Woodside, M. Addressing the Needs of First-Generation College Students: Lessons Learned From Adults From Low-Education Families. *Journal of College Counseling* 17, 21–36 (2014).

Gibbons, M. M. & Borders, L. D. Prospective First-Generation College Students: A Social-Cognitive Perspective. *Career Dev Q* 58, 194–208 (2010).

Gist-Mackey, A. N., Wiley, M. L., & Erba, J. "You're doing great. Keep doing what you're doing": socially supportive communication during first-generation college students' socialization. *Communication Education*, *67*(1), 52–72 (2017).

Goldman, J., Cavazos, J., Heddy, B. C., & Pugh, K. J. Emotions, values, and engagement: Understanding motivation of first-generation college students. *Scholarship of Teaching and Learning in Psychology, 10*(1), 1–15 (2024).

Gorard, S., & Smith, E. Beyond the 'learning society': what have we learnt from widening participation research? *International Journal of Lifelong Education*, *25*(6), 575–594 (2006).

Henderson et. al. 'First in Family' University Graduates in England | IZA - Institute of Labor Economics. (2019). Retrieved from https://www.iza.org/publications/dp/12588/first-in-family-university-graduates-in-england.

House, L. A., Neal, C. & Kolb, J. Supporting the Mental Health Needs of First Generation College Students. *J College Stud Psychother* 34, 157–167 (2020).

Ireland, E., Golden, S. & Morris, M. Evaluation of Integrated Aimhigher: Tracking Surveys of Young People. (2006).

Janke, S., Rudert, S. C., Marksteiner, T. & Dickhäuser, O. Knowing one's place: Parental Educational Background Influences Social Identification With Academia, test anxiety, and satisfaction with studying at university. *Front Psychol* 8, (2017).

Jenkins, S. R., Belanger, A., Connally, M. L., Boals, A. & Durõn, K. M. First-generation undergraduate students' social support, depression, and life satisfaction. *Journal of College Counseling* 16, 129–142 (2013).

Jones, P. J., Park, S. Y., & Lefevor, G. T. Contemporary college student anxiety: The role of academic distress, financial stress, and support. *Journal of College Counseling, 21*(3), 252–264 (2018)

Kurotsuchi Inkelas, K., Daver, Z. E., Vogt, K. E. & Brown Leonard, J. Living-learning programs and first-generation college students' academic and social transition to college. Research in Higher Education 48 (4), 403-434 (2006).

Lev Levine, K. J. & Aley, M. Career Barriers Affecting First-Generation College Students: Can Socializing Messages Increase Career Confidence? *The Southern Communication Journal*86, 498–510 (2021).

Long Longwell-Grice, R., Adsitt, N. Z., Mullins, K. & Serrata, W. The First Ones: Three Studies on First-Generation College Students. *NACADA Journal* 36, 34–46 (2016).

López, M. J., Santelices, M. V. & Taveras, C. M. Academic performance and adjustment of first-generation students to higher education: A systematic review. *Cogent Education* 10, (2023).

Ma, P.-W. W., & Shea, M. First-Generation College Students' Perceived Barriers and Career Outcome Expectations: Exploring Contextual and Cognitive Factors. Journal of Career Development, 48(2), 91-104. (2021).

Means, D. R. & Pyne, K. B. Finding my way: Perceptions of institutional support and belonging in low-income, first-generation, first-year college students. *J Coll Stud Dev* 58, 907–924 (2017).

Moore, J., Sanders, J. & Higham, L. Literature review of research into widening participation to higher education. Report to HEFCE and OFFA by ARC Network August 2013. (2013).

Moschetti, R. V. & Hudley, C. Social Capital and Academic Motivation Among First Generation Community College Students. *Community Coll J Res Pract* 39, 235–251 (2015).

Nguyen, T.-H., & Nguyen, B. M. D. Is the "First-Generation Student" Term Useful for Understanding Inequality? The Role of Intersectionality in Illuminating the Implications of an Accepted—Yet Unchallenged—Term. *Review of Research in Education*, *42*(1), 146-176 (2018)

Noel, J. K., Lakhan, H. A., Sammartino, C. J. & Rosenthal, S. R. Depressive and anxiety symptoms in first generation college students. *J Am Coll Health* 71, 1906–1915 (2023).

Oliver, A. I. G. & King, C. A. Reaching Individual Success and Empowerment (RISE): A First-generation, Co-curricular, Academic, and Social Engagement Model. *Journal of Business Diversity* 18, (2018).

Park Parker M. & Pollock, D. First gen, PhD: Understanding the information horizons of first-generation graduate students pursuing research-intensive careers. *Education (Chula Vista)*(2019).

Pascarella, E. T., Pierson, C. T., Wolniak, G. C., & Terenzini, P. T. (First-Generation College Students: Additional Evidence on College Experiences and Outcomes. *The Journal of Higher Education*, *75*(3), 249–284. (2004)

Patfield, S., Gore, J. & Fray, L. Reframing first-generation entry: how the familial habitus shapes aspirations for higher education among prospective first-generation students. *Higher Education Research and Development* 40, 599–612 (2021).

Quantifying Differences in Cultural Capital of First- and Continuing-Generation Undergraduates: Parental Support, Involvement, and Communication. FirstGenFORWARD. (2020).

Pike, G. R., & Kuh, G. D. First- and Second-Generation College Students: A Comparison of Their Engagement and Intellectual Development. *The Journal of Higher Education*, *76*(3), 276–300. (2005).

Press Pressimone Beckowski, C. M. & Winfield, J. D. Toward a Culture of First Generation Student Success: An Analysis of Mission Statements From First-gen Forward Institutions. *Journal of First-generation Student Success* 1, 73–91 (2021).

Lynch, J., Walker-Gibbs, B. & Herbert, S. Re-conceptualising sustainable widening participation: evaluation, collaboration and evolution. *Higher Education Research & Development* 34, 383–396 (2014).

Rockwell, D. M. & Kimel, S. Y. A systematic review of first-generation college students' mental health. *J Am Coll Health* (2023).

Roksa, J., Feldon, D. F. & Maher, M. The Journal of Higher Education First-Generation Students in Pursuit of the PhD: Comparing Socialization Experiences and Outcomes to Continuing-Generation Peers. (2018).

Rondini, A. C. "Dream like the Whites": Disjunctures in Racial Experiences and Interpretations of Low-Income First-Generation Students of Color and Their Parents. *Soc Probl* 70, 616–634 (2023).

Rowan-Kenyon et al. Technology and Engagement. *Technology and Engagement* (2018).

Shandera, S., Matsick, J. L., Hunter, D. R., & Leblond, L. RASE: Modeling cumulative disadvantage due to marginalized group status in academia. *PloS one*, *16*(12). (2021).

Soria Soria, K. M. & Stebleton, M. J. First-generation students' academic engagement and retention. *Teaching in Higher Education* 17, 673–685 (2012).
Stephens, N. M., Fryberg, S. A., Markus, H. R., Johnson, C. S., & Covarrubias, R. Unseen disadvantage: How American universities' focus on independence undermines the academic performance of first-generation college students. *Journal of Personality and Social Psychology, 102*(6), 1178–1197 (2012).
Strayhorn, T. L. Factors Influencing the Academic Achievement of First-Generation College Students. *NASPA Journal*, *43*(4), 82–111 (2006)
Tominaj, T. PERCEPTIONS OF 1 ST GENERATION COLLEGE STUDENTS. Cedar Crest College. (2021).
Toyokawa, T. & DeWald, C. Perceived Career Barriers and Career Decidedness of First-Generation College Students. *Career Development Quarterly* 68, 332–347 (2020).
Warbuton, E.C., Bugarin, R., Nuñez, A-M. & Caroll, C.D. Statistical Analysis Report Postsecondary Education Descriptive Analysis Reports Bridging the Gap Academic Preparation and Postsecondary Success of First-Generation Students. (2001).

**Category 3: STEM**
Allen, J. M., Muragishi, G. A., Smith, J. L., Thoman, D. B. & Brown, E. R. To Grab and To Hold: Cultivating communal goals to overcome cultural and structural barriers in first generation college students' science interest. *Transl Issues Psychol Sci* 1, 331–341 (2015).
Armstrong, M. A., & Jovanovic, J. The intersectional matrix: Rethinking institutional change for URM women in STEM. *Journal of Diversity in Higher Education, 10*(3), 216–231 (2017).
Besecke et al. Factors Influencing Career Choice for Women in Science, Mathemathics, and Technology. Advancing Women in Leadership Journal, 21, (2021).
Bett Bettencourt, G. M., Manly, C. A., Kimball, E. & Wells, R. S. STEM degree completion and first-generation college students: A cumulative disadvantage approach to the outcomes gap. *Review of Higher Education* 43, 753–779 (2020).
Canning, E. A., LaCosse, J., Kroeper, K. M., & Murphy, M. C. Feeling Like an Imposter: The Effect of Perceived Classroom Competition on the Daily Psychological Experiences of First-Generation College Students. *Social Psychological and Personality Science*, *11*(5), 647-657 (2020).

Chen, S., Binning, K. R., Manke, K. J., Brady, S. T., McGreevy, E. M., Betancur, L., Limeri, L. B., & Kaufmann, N. Am I a Science Person? A Strong Science Identity Bolsters Minority Students' Sense of Belonging and Performance in College. *Personality and Social Psychology Bulletin*, *47*(4), 593-606 (2021).

Codiroli Mcmaster, N. Who studies STEM subjects at A level and degree in England? An investigation into the intersections between students' family background, gender and ethnicity in determining choice. *Br Educ Res J* 43, 528–553 (2017).

Dawson, E. Equity, Exclusion and Everyday Science Learning. *Equity, Exclusion and Everyday Science Learning* (2019).

Dewsbury, B. M., Taylor, C., Reid, A. & Viamonte, C. Career Choice among First-Generation, Minority STEM College Students. *J Microbiol Biol Educ* 20, (2019).

Ellis, J. M., Powell, C. S., Demetriou, C. P., Huerta-Bapat, C., & Panter, A. T. Examining first-generation college student lived experiences with microaggressions and microaffirmations at a predominately White public research university. *Cultural Diversity & Ethnic Minority Psychology, 25*(2), 266–279. (2019).

Estrada, M. *et al.* Improving Underrepresented Minority Student Persistence in STEM. *CBE Life Sci Educ* 15, (2016).

National Data Fact Sheets on First-generation College Students. https://firstgen.naspa journal-and-research/national-data-fact-sheets-on-first-generation-college-students-and-graduates-2023..org/

Dalton Foltz, L. G. Factors That Contribute to the Persistence of Minority Students in STEM Fields. Planning for Higher Education 42(4) (2014).

Gangitano G. College Transition : Voices of First-Generation Minority STEM Students. *Theses, Dissertations and Culminating Projects* (2021).

Gayles, J. G. & Smith, K. N. Advancing Theoretical Frameworks for Intersectional Research on Women in STEM. *New Directions for Institutional Research* 2018, 27–43 (2018).

Goodman, I. F. *et al.* Final report of the women's experiences in college engineering (WECE) project. (2002)

Goonewardene, A., Offutt, C., Whitling, J. & Woodhouse, D. An Interdisciplinary Approach to Success for Underrepresented Students in STEM. *J Coll Sci Teach* 045, (2016).

Henley MEd, L. & Roberts, P. M. Perceived Barriers to Higher Education in STEM Among Disadvantaged Rural Students: A Case Study. *Inquiry: The Journal of the Virginia Community Colleges* 20 (2016).

Ireland, D. T., Freeman, K. E., Winston-Proctor, C. E., DeLaine, K. D., McDonald Lowe, S., & Woodson, K. M. (Un)Hidden Figures: A Synthesis of Research Examining the Intersectional Experiences of Black Women and Girls in STEM Education. *Review of Research in Education*, *42*(1), 226-254 (2018).

Ro, H. K., Fernandez, F. & Alcott, B. Social Class, Human Capital, and Enrollment in STEM Subjects at Prestigious Universities: The Case of England. *Educational Policy* 35, 422–449 (2021).

Tougher grading is one reason for high STEM dropout rate | Cornell Chronicle. https://news.cornell.edu/stories/2010/04/tougher-grading-one-reason-high-stem-dropout-rate#. (2010).

MacPhee, D., Farro, S., & Canetto, S. S. Academic self-efficacy and performance of underrepresented STEM majors: Gender, ethnic, and social class patterns. *Analyses of Social Issues and Public Policy (ASAP), 13*(1), 347–369 (2013).

Martin, J. P., Stefl, S. K., Cain, L. W. & Pfirman, A. L. Understanding first-generation undergraduate engineering students' entry and persistence through social capital theory. *Int J STEM Educ* 7, 1–22 (2020).

Metcalf, H., Russell, D., & Hill, C. Broadening the science of broadening participation in STEM through critical mixed methodologies and intersectionality frameworks. *American Behavioral Scientist, 62*(5), 580–599 (2018).

M , Morgan, M. Study expectations of 1st/2nd generation STEM postgraduate taught students. *Quality Assurance in Education* 22, 169–184 (2014).

Myers, C. B. & Pavel, D. M. Underrepresented students in STEM: The transition from undergraduate to graduate programs. *J Divers High Educ* 4, 90–105 (2011).

Women, Minorities, and Persons with Disabilities in S&E | NCSES | NSF. https://www.nsf.gov/statistics/women/. (2021).

Open University UK. Horizons in STEM Higher Education Conference Proceedings Making Connections, Innovating and Sharing Pedagogy. (2021).

Snodgrass Rangel, V., Vaval, L. & Bowers, A. Investigating underrepresented and first-generation college students' science and math motivational beliefs: A nationally representative study using latent profile analysis. *Sci Educ* 104, 1041–1070 (2020).

Shedlosky-Shoemaker, R. & Fautch, J. M. Who leaves, who stays? Psychological predictors of undergraduate chemistry students' persistence. *J Chem Educ* 92, 408–414 (2015).

Steams, E., Mickelson, A. E., Moller, S., Bottia, C. M. ROOTS of STEM | Executive Summary. http://pages.charlotte.edu/rootsofstem/executive-summary/. (2010).

Strayhorn, T. L. Work in progress - Academic and social barriers to black and latino male collegians in engineering. *Proceedings - Frontiers in Education Conference, FIE* (2009).

Uche, A. *The Retention Of First-Generation College Students In Stem: An Extension Of Tinto's Longitudinal Model*. *Unc Charlotte Electronic Theses And Dissertations*. (2015).

Verdin, D., Godwin, A., Sonnert, G. & Sadler, P. M. Understanding how first-generation college students' out-of-school experiences, physics and STEM identities relate to engineering possible selves and certainty of career path. *Proceedings - Frontiers in Education Conference, FIE* 2018-October, (2018).

Voigt, M., Hagman, J.E., Gehrtz, J., Ratliff, B., Alexander, N.N., & Levy, R.A. Justice through the lens of calculus: Framing new possibilities for diversity, equity, and inclusion. (2021).

Whitcomb, K. M., Cwik, S. & Singh, C. Not All Disadvantages Are Equal: Racial/Ethnic Minority Students Have Largest Disadvantage Among Demographic Groups in Both STEM and Non-STEM GPA. *AERA Open* 7, (2021).

Whittaker J. A. & Montgomery, B. L. Cultivating Diversity and Competency in STEM: Challenges and Remedies for Removing Virtual Barriers to Constructing Diverse Higher Education Communities of Success. *Journal of Undergraduate Neuroscience Education* 11, A44 (2012).

Wilkins-Yel, K. G., Bernstein, B. L., Bekki, J. M., Zounlome, N. O. & Reed, A. Intersectional perspectives: Interpersonal contributors to moments of doubt for graduate women of color in STEM. *CoNECD 2019 - Collaborative Network for Engineering and Computing Diversity* (2019).

Category 4: Chemistry

Arseneau, J. R. *et al.* Career Success of Women in the Chemical Industry, Part 2: Navigating Workplace Challenges. *ACS Symposium Series* 1255, 145–172 (2017).

Mallaburn, A., Seton, L. & Brennan, V. Widening Participation By Effective Outreach In Chemistry. *Edulearn21 Proceedings* 1, 10729–10729 (2021).

Paquin, J. D. *et al.* Career Success of Women in the Chemical Industry, Part 3: Getting on the Same Page. *ACS Symposium Series* 1255, 173–204 (2017).

Smith, D. K. The race to the bottom and the route to the top. *Nature Chemistry 2020 12:2*12, 101–103 (2020).

Cossairt, B. M., Dempsey, J. L. & Young, E. R. The Chemistry Women Mentorship Network (ChemWMN): A Tool for Creating Critical Mass in Academic Chemistry. *ACS Cent Sci* 5, 1625–1629 (2019).

Davis, R. D., Winfield, L., Spivak, D. & Wilson-Kennedy, Z. S. Role of International Research Experiences in the Development of Women of Color in Chemistry. *J Chem Educ*99, 104–112 (2022).

Damkaci, F., Braun, T. F. & Gublo, K. Peer Mentor Program for the General Chemistry Laboratory Designed To Improve Undergraduate STEM Retention. *J Chem Educ* 94, 1873–1880 (2017).

Category 5: EDI

Adams, T. L. & McBrayer, J. S. The Lived Experiences of First-Generation College Students of Color Integrating into the Institutional Culture of a Predominantly White Institution. *The Qualitative Report* 25, 733–757 (2020).

Alrehaly, E. D. Parental Attitudes and the Effects of Ethnicity: How They Influence Children's Attitudes toward Science Education. *Online Submission* (2011).

Bañuelos, M. & Flores, G. M. 'I could see myself': professors' influence in first-generation Latinx college students' pathways into doctoral programs. *Race Ethn Educ* 27, 599–619 (2024).

Bowl, M. Experiencing the barriers: Non-traditional students entering higher education. *Res Pap Educ* 16, 141–160 (2001).

Canfield, K. N. *et al.* Science Communication Demands a Critical Approach That Centers Inclusion, Equity, and Intersectionality. *Front Commun (Lausanne)* 5, 488799 (2020).

Daniels, J. *et al.* Improving Completion Rates for Underrepresented Populations. *Inquiry: The Journal of the Virginia Community Colleges* 22, (2019).

Davis, R. D., Winfield, L., Spivak, D. & Wilson-Kennedy, Z. S. Role of International Research Experiences in the Development of Women of Color in Chemistry. *J Chem Educ* 99, 104–112 (2022).

Dennis, J. M., Phinney, J. S. & Chuateco, L. I. The role of motivation, parental support, and peer support in the academic success of ethnic minority first-generation college students. *J Coll Stud Dev* 46, 223–236 (2005).

Garriott, P. O., Ko, S. J. “Stella”, Grant, S. B., Jessen, M. & Allan, B. A. When Race and Class Collide: Classism and Social-Emotional Experiences of First-Generation College Students 25, 509–532 (2021).

Crozier, G., Reay, D., Clayton, J., Colliander, L. & Grinstead, J. Different strokes for different folks: diverse students in diverse institutions – experiences of higher education. *Res Pap Educ* 23, 167–177 (2008).

Henderson, C. E. All Eyez on Me: On Being Black, Female, and a First-Gen Leader in the Academy. *Palimpsest: A Journal on Women, Gender, and the Black International* 10, 125–133 (2021).

Herrmann, S. D., Varnum, M. E. W., Straka, B. C. & Gaither, S. E. Social Class Identity Integration and Success for First-Generation College Students: Antecedents, Mechanisms, and Generalizability. *Self and Identity* (2022).

Herrmann, S. D. & Varnum, M. E. W. Utilizing social class bicultural identity integration to improve outcomes for first-generation college students. *Transl Issues Psychol Sci* 4, 165–175 (2018).

Jack, A. A. Culture shock revisited: The social and cultural contingencies to class marginality. *Sociological Forum* 29, 453–475 (2014).

Kennedy S. A. *et al.* Faculty Professional Development on Inclusive Pedagogy Yields Chemistry Curriculum Transformation, Equity Awareness, and Community. *J Chem Educ* 99, 291–300 (2022).

Miele, J. & Nguyen, V. H. On par with the rest: first-generation college students and cultural intelligence. *J Furth High Educ* 44, 809–817 (2020).

Montero-Hernandez, V. & Drouin, S. The journey into a New me: Narratives of self-development and community revitalization among first-generation, Latinx, adult graduate students. *Journal of Adult and Continuing Education* 27, 175–204 (2021).

Nguyen, B. M. D. & Nguyen, M. H. Extending cultural mismatch theory: in consideration of race/ethnicity. *International Studies in Sociology of Education* 29, 224–249 (2020).

Nichols, S. & Stahl, G. Intersectionality in higher education research: a systematic literature review. *Higher Education Research and Development* 38, 1255–1268 (2019).

Reay, D., Gill Crozier, ; & Clayton, J. ‘Fitting in’ or ‘standing out’: working-class students in UK higher education. (2009).

Roksa, J. & Kinsley, P. The Role of Family Support in Facilitating Academic Success of Low-Income Students. *Res High Educ* 60, 415–436 (2019).

Santa-Ramirez, S., Wells, T., Sandoval, J. & Koro, M. Working through the experiences of first-generation students of color, university mission, intersectionality, and post-subjectivity. *International Journal of Qualitative Studies in Education* 35, 109–124 (2022).

Sheared, V. *et al. Race and Adult Education The Handbook of Race Adult Education*. (2010).

Torres, V. N. Ascendance and Transformation: Humanizing TRIO First Generation Students of Color and their STEM Empowerment Agents. *The Macksey Journal* 1, 58 (2020).

Williams, Q., Williams, B. M. & Brown, L. C. Exploring Black Girl Magic: Identity Development of Black First-Gen College Women. *J Divers High Educ* 15, 466–479 (2022).

Categories 6: Women

Bagihole et. al. (2013) - Generation And Gender In Academia

Buchmann, C., & DiPrete, T. A. The Growing Female Advantage in College Completion: The Role of Family Background and Academic Achievement. *American Sociological Review*, *71*(4), 515-541 (2006).

Kim J, Miller, S. M., Hwang, J. & Olson, J. S. Female first-generation college students: A review of challenges and successes. *Journal of Global Education and Research* 5, 136–150 (2021).

Category 7: Mentoring

Ahmed, M., Muldoon, T. J. & Elsaadany, M. Employing Faculty, Peer Mentoring, and Coaching to Increase the Self-Confidence and Belongingness of First-Generation College Students in Biomedical Engineering. *J Biomech Eng* 143 (2021).

Ceballos, N. The first in my family: mentoring first generation college students in academia and beyond. *Proceedings of the 2015 Mentoring Conference, pages 131 – 134 (2015).*

Cruz, C., Rajpal, G., Lecocke, M., Martines, I., & Lurie, A. Peer Coaching Program Development: A Framework of First-Year Latina/o Student Persistence Pursuing STEM Pathways at a Hispanic Serving Institution. *Journal of Hispanic Higher Education*, *20*(4), 365-384 (2021).

Curtin, N., Malley, J. & Stewart, A. J. Mentoring the Next Generation of Faculty: Supporting Academic Career Aspirations Among Doctoral Students. *Res High Educ* 57, 714–738 (2016).

Damkaci, F., Braun, T. F. & Gublo, K. Peer Mentor Program for the General Chemistry Laboratory Designed To Improve Undergraduate STEM Retention. *J Chem Educ* 94, 1873–1880 (2017).

Marshall, A. G. *et al.* The importance of mentors and how to handle more than one mentor. *Pathog Dis* 80, (2022).

Rockinson-Szapkiw, A. & Wendt, J. L. The benefits and challenges of a blended peer mentoring program for women peer mentors in science, technology, engineering and mathematics (STEM). *International Journal of Mentoring and Coaching in Education*10, 1–16 (2021).

Table S5: Full list of survey questions.

Survey Question	
**1.**	What stage of your study or career are you at?
**2.**	What is your gender?
**3.**	Which country are you located in for your work or study?
**4.**	Which country were you born and/or raised in?
**5.**	If you have moved countries, please would you tell us whether it was for career or personal reasons?
**6.**	How would you describe your ethnicity?
**7.**	Do you have any protected equality, diversity and inclusion characteristics (these include race, religion, sexuality for example)?
**8.**	Do you have caring responsibilities?
**9.**	If yes, who do you care for?
**10.**	Would you consider yourself a supramolecular chemist?
**11.**	Do you consider yourself 1st Gen (that is the first in your family to enter Higher Education at university or college level)<br>a. If you are not sure or feel conflicted about your answer please explain why.
**12.**	How did you decide what you wanted to study? How did you inform yourself about your choice? Who supported you in this process?
**13.**	What fears did you have with regard to your chosen field of study or your professional future?
**14.**	Was the financing of your studies an issue that worried you before or during your studies? If so, why?
**15.**	Who did you go to for the resources and support that you needed to begin your studies or academic career?
**16.**	Did you know a lot about career paths for the future generally or in supramolecular chemistry specifically when your started studying? If not, where and when did you get this information?
**17.**	How will you or did you make career decisions after completing your undergraduate degree? Did you receive support or advice regarding your decision and career choice? If so, who supported you?
**18.**	If applicable, what contributed to your decision to study or persist in supramolecular chemistry?
**19.**	Did you stay with the same research group after completing your undergraduate or postgraduate degree, or, if you have not finished your studies would you want to stay with the same group or change group? What are the reasons for this?
**20.**	Has there been anything that feels unfamiliar or alien to you regarding your academic studies or career?
**21.**	How conscious are you of being – or someone in your private or professional environment being – the first in your/their family to attend college or university? What does this mean to you/them?

**22.**	In your opinion, what personal and educational experiences do 1st Gen chemists have that non 1st Gen chemists do not?
**23.**	If you supervise 1st Gen students, do you try to support them in a special way? If so, how? If you are 1st Gen, what kind of supervision did you get or need?
**24.**	Whether you are 1st Gen or not, would you say that being First Generation in academia had or still has any disadvantages? If yes, how would you describe them?
**25.**	If you were to give one piece of advice to a 1st Gen chemistry student, or to a supervisor of a 1st Gen chemistry student, what would it be?
**26.**	Please tell us if you have any thoughts or ideas about how you would like to see the WISC network support the retention and progression of 1st Gen scientists and supramolecular chemists?

Table S6: Study 1 participant demographics.

Demographic	Frequency	Percentage
Respondents	136	100
First Gen	45	33.1
not First Gen	91	66.9
Men	56	41.2
Women	76	55.9
Undergraduate	17	12.5
Postgraduate	61	44.9
Early career	11	8.1
Mid career	26	19.1
Late career	12	8.8
Other	3	2.2
Missing data	2	1.5
white	104	76.5
not white	32	23.5
At least one protected characteristic	49	36
Disabled	3	2.2
Racially minoritised	2	1.5
Religious minority	3	2.2
LGBTQIA+	6	4.4
Caring for children	27	19.9
Caring for elderly	9	6.6
Moved country	106	77.9
Did not move country	30	22.1
Continent of work or study		4
Africa	6	4.4
Asia	5	3.7
Europe	94	69.1
North America	25	18.5

Australia	5	3.7
Missing	1	0.7
Continent of birth		
Africa	8	5.9
Asia	14	10.3
Europe	87	64
North America	17	12.5
Australia	5	3.7
South America	1	0.7
Missing	4	2.9

Table S6a: Cross-tabulation of gender and being First Gen.

			not first gen	first gen	total
What is your gender-categorized	woman	Count	24	52	76
		% of Total	17.8%	38.5%	56.3%
	other	Count	1	2	3
		% of Total	0.7%	1.5%	2.2%
	man	Count	19	37	56
		% of Total	14.1%	27.4%	41.5%
Total		Count	44	91	135
		% of Total	32.6%	67.4%	100.0%

Table S6b: Cross-tabulation of career stage and being First Gen.

			not first gen	first gen	total
What stage of your study or career are you at-categorized	undergraduate	Count	5	12	17
		% of Total	3.7%	9.0%	12.7%
	postgraduate	Count	22	39	61
		% of Total	16.4%	29.1%	45.5%
	other	Count	4	3	7
		% of Total	3.0%	2.2%	5.2%
	mid stage professional	Count	7	19	26
		% of Total	5.2%	14.2%	19.4%
	later stage professional	Count	4	8	12
		% of Total	3.0%	6.0%	9.0%
	early stage professional	Count	2	9	11
		% of Total	1.5%	6.7%	8.2%
Total		Count	44	90	134
		% of Total	32.8%	67.2%	100.0%

Table S6c: Cross-tabulation of protected characteristics and being First Gen.

			not first gen	first gen	total
Protected characteristics - categorized	sexual orientation	Count	4	2	6
		% of Total	3.6%	1.8%	5.5%
	religion	Count	1	2	3
		% of Total	0.9%	1.8%	2.7%
	race	Count	0	2	2
		% of Total	0.0%	1.8%	1.8%
	none	Count	30	57	87
		% of Total	27.3%	51.8%	79.1%
	more than one	Count	1	2	3
		% of Total	0.9%	1.8%	2.7%
	disability	Count	1	2	3
		% of Total	0.9%	1.8%	2.7%
	Yes	Count	0	6	6
		% of Total	0.0%	5.5%	5.5%
Total		Count	37	73	110
		% of Total	33.6%	66.4%	100.0%

Table S6d: Cross-tabulation of ethnicity and being First Gen.

			not first gen	first gen	total
Ethnicity – categorized	White	Count	34	70	104
		% of Total	26.2%	53.8%	80.0%
	Non-white	Count	9	17	26
		% of Total	6.9%	13.1%	20.0%
Total		Count	43	87	130
		% of Total	33.1%	66.9%	100.0%

Table S6e: Cross-tabulation of moving country and being First Gen.

		not first gen	first gen	total
did not move country	Count	10	20	30
	% of Total	7.4%	14.7%	22.1%
moved country	Count	35	71	106
	% of Total	25.7%	52.2%	77.9%
Total	Count	45	91	136

Table S6f: Cross-tabulation of caring responsibilities and being First Gen.

			not first gen	first gen	total
Carer responsibilities - categorized	yes	Count	15	25	40
		% of Total	11.1%	18.5%	29.6%
	no	Count	29	66	95
		% of Total	21.5%	48.9%	70.4%
Total		Count	44	91	135
		% of Total	32.6%	67.4%	100.0%

Table S6g: Cross-tabulation of identifying as a supramolecular chemist and being First Gen.

			not first gen	first gen	total
Supramolecular chemist - categorized	yes	Count	28	56	84
		% of Total	20.7%	41.5%	62.2%
	no	Count	16	35	51
		% of Total	11.9%	25.9%	37.8%
Total		Count	44	91	135
		% of Total	32.6%	67.4%	100.0%

Table S7: Codebook from Study 2. Parent codes are indicated in bold.

Code Name	Code description
**First Gen disadvantages**	Concerning hardships associated with First Gen status as described by participants.
Anxiety and stress	
Belonging	
Confidence	
Family understanding	
Finances	
Guidance and expectations	
Isolation	
Mental burden	
Motivation	
Networking	
Seeking help	
Lack of skills	
Fear of leaving family	
**Progression and retention**	This theme describes how people describe moving from one stage of their academic career to the next.
Balance and wellbeing	
'easier' option	
Intuitive next step	
Less obvious or harder choice	
**Advice for First Gens**	Participants, whether identifying as First Gen themselves or not, offered some advice to First Gen and chemistry.
Belonging and confidence	
Experiences and networking	
Mentors	
Resources and information	
Resilience	
Seeking help	
**Bias and marginalisation**	Some participants explicitly mentioned experiences bias and marginalisation during their research careers.
**Capital**	Capital here is referred to as the types of knowledge and understandings described by participants as needed for progression through the academic pathway.

Academic careers and research	
Application processes	
Career	
How to work	
Networking	
Science and chemistry relevant	
**Chemicals, illness and pregnancy**	Some participants mentioned worry and stress associated with exposure to chemicals and experiencing illness. A few participants expressed being specifically concerned about chemical exposure during pregnancy.
**Competitive landscape**	Some participants mentioned worries associated with the competitive landscape in research and in science/chemistry.
**Conflicting commitments (work or personal)**	Some participants mentioned the challenge balancing their research careers with personal and additional work commitments.
**Employment opportunities and career enhancements**	Some participants described their pursuit of academic careers as an opportunity to enhance their current careers and/or their employment opportunities.
**Expression of no support**	Some participants described having had no support before or during their academic careers.
**Feelings about 1st Gen status**	The child codes to this parent code categorise participant feelings associated with First Generation status, whether your own or someone else's.
Conscious for others	
Family and parents	
Gratitude	
Not conscious	
Other disadvantages	
Pride	
Self-consciousness	
Additional pressure and responsibility	
**Financial burden**	This code categorises participants as to whether they expressed experiencing financial burden with child codes, 'yes' or 'no'.
No	
Yes	
**Feeling about financing academia**	This code categorises feelings associated with the financing of academia into negative or positive sentiment and describe said feelings.

NEGATIVE	
POSITIVE	
Dependants	
Employment alongside studies	
Inadequate	
Lack of familial support	
Lack of governmental support	
Familial support	
Other funding	
Scholarships and grants	
**Immigration and language related challenges**	Some participants described experiencing challenges specifically associated with moving countries and/or learning languages in pursuit of their academic career.
**Imposter feelings**	Some participants described experiencing what is commonly known as ‘imposter syndrome’ and discussed as ‘imposter experience/feelings’ throughout this study.
Belonging feelings	
**Institutional support**	Some participants describe different kinds of institutional support during their academic careers which are categorised by child codes under this parent code.
Research Environment	
**Research group retention**	Participants were asked if they stayed or left their research group after studying for their post graduate degree (or what they were planning to do if still studying).
Left	
Other	
Stayed	
**Mental health concerns**	Some participants describe concerns or challenges related to their mental health associated with their research studies and/or career.
**Motherhood and parenthood**	Some participants mentioned experiences specifically related to being a mother and/or a women associated with their experiences of research.
**Natural Affinity and Competence**	Some participants described following their natural affinity or competence/talent in pursuing chemistry research. Self-determination theory defines desirability for competence as one of the three key determinates of student and worker decisions.
**Personal interest or curiosity**	Some participants described interest or curiosity in addition or instead of competence for their decision.

**Non-state and independent groups**	Some participant name and mention independent groups and organisations as key resources in entering or progressing in their research studies and/or career.
**Occupational and academic mentors**	Some participants described mentors in their workplace or research institution who were integral to their entry or progression in research. In this instance, 'mentor' was defined as a figure senior in their career or studies to the individual, who was depended on for information, guidance and/or support.
Other 'mentor'	
Supervisors and PIs	
Directing to recourses and information	
Emotional and mental support	
Other	
Role modelling	
**Online recourses**	Some participants described using websites and other online recourses as integral sources of information and guidance concerning entry to or progression withing research.
**Peer Behaviours and Support**	Some participants mentioned relying on their peers for information, support and as inspiration for entering and progressing in research. Literature suggests that friends and school peers are significantly influential in the decision making and engagement of students.
**Perceived or actual workload**	Some participants described what they imagined or experienced the workload to be as a barrier to entry and/or progression in research.
**Prestige and personal betterment**	Some participants mentioned prestige or personal betterment as motivation for entry and/or progression within research.
**Race and ethnicity related worries**	Some participants described race and ethnicity related worries as a barrier to entry and/or progression within academia.
**Religious influence**	Some participants described religious beliefs influencing their decisions regarding entry and/or progression within research.
**School and Teachers**	Some participants described their school experiences as well as individual teachers as integral recourses to entry into higher education.
**Science specific challenges**	Some participants described the nature of scientific research specifically, as opposed to other disciplines, as a challenge or barrier to entry and/or progression within research.
**State support**	Some participants described receiving support from the state as integral to their entry and/or progression within research.

**Success in science**	Some participants defined what "success in their scientific careers/studies" could be defined as for them.
Autonomy	
Culture	
Funding	
Recognition	
Scientific breakthrough	
Wellbeing and content	
**Successful Traits**	Some participants described individual traits they perceived to be traits of a successful person.
Hard work and resilience	
Passion and interest	
**Family role**	Some participants described their family's role as an integral recourse in their entry and or/progression within research.
Academic recourses	
Financial	
Mental and emotional	
Science or chemistry related	
**Unfamiliarity**	Some participants described experiencing unfamiliarity during their research studies and/or career.
Academic challenge	
Funding	
New country	
Next steps	
None	
Other	
Process and politics	
Several	
Underrepresented	
**Work experience and internships**	Some participants described work experience and internships as an integral resource to their entry and/or regression within research.

Table S8a: Correlations between being First Gen & feeling financial burden.

Model		Unstandardized Coefficients		Standardized Coefficients		
		B	Std. Error	Beta	t	Sig.
1	(Constant)	.222	.071		3.123	.002
	first gen	.283	.087	.271	3.257	.001

Dependent Variable: do they feel a financial burden?

Table S8b: Correlations with feeling financial burden.

Model		Unstandardized Coefficients		Standardized Coefficients		
		B	Std. Error	Beta	t	Sig.
1	(Constant)	.209	.095		2.188	.030
	first gen	.286	.088	.273	3.245	.001
	dummy variable man	-.057	.084	-.057	-.680	.498
	at least one protected characteristic	.027	.089	.026	.300	.765
	do you have caring responsibilities?	.078	.094	.072	.826	.410
	dummy for moved country	.013	.101	.011	.132	.895

Dependent Variable: do they feel a financial burden?

Table S8c: Correlations being First Gen & receiving family support.

Model		Unstandardized Coefficients		Standardized Coefficients		
		B	Std. Error	Beta	t	Sig.
1	(Constant)	.444	.064		6.911	<.001
	first gen	-.258	.079	-.272	-3.277	.001

Dependent Variable: family support

Table S8d: Correlations with receiving family support.

Model		Unstandardized Coefficients		Standardized Coefficients	t	Sig.
		B	Std. Error	Beta		
1	(Constant)	.520	.085		6.144	<.001
	first gen	-.258	.078	-.273	-3.297	.001
	dummy variable man	-.139	.075	-.154	-1.855	.066
	at least one protected characteristic	.004	.079	.005	.057	.955
	do you have caring responsibilities?	.044	.083	.045	.528	.599
	dummy for moved country	-.150	.090	-.140	-1.678	.096

Dependent Variable: family support

Table S8e: Correlation of no support and being First Gen.

Model		Unstandardized Coefficients B	Std. Error	Standardized Coefficients Beta	t	Sig.
1	(Constant)	1.388E-17	.037		.000	1.000
	first gen	.099	.045	.187	2.206	.029

Dependent Variable: expression of no support

Table S8f: Correlation of isolation with imposter syndrome.

		Unstandardized Coefficients		Standardized Coefficients		
Model		B	Std. Error	Beta	t	Sig.
1	(Constant)	.208	.037		5.596	<.001
	do they struggle with isolation	.428	.131	.272	3.278	.001

Dependent Variable: do they have imposter syndrome?

Table S8g: Correlation of UG with caring responsibilities.

Model		Unstandardized Coefficients B	Std. Error	Standardized Coefficients Beta	t	Sig.
1	(Constant)	.336	.041		8.237	<.001
	dummy variable for undergraduates	-.336	.115	-.244	-2.912	.004

Dependent Variable: do you have caring responsibilities?

Table S8h: Correlation of MCR with moving country.

		Unstandardized Coefficients		Standardized Coefficients		
Model		B	Std. Error	Beta	t	Sig.
1	(Constant)	.173	.039		4.464	<.001
	dummy variable for mid-stage researcher	.250	.088	.237	2.829	.005

Dependent Variable: dummy for did not move

Table S8i: Correlation of gender and online resources.

	Unstandardized Coefficients		Standardized Coefficients		
	B	Std. Error	Beta	t	Sig.
(Constant)	.175	.037		4.777	<.001
dummy variable man	-.121	.057	-.181	-2.127	.035

Dependent Variable: using online resources

Table S8j: Correlation of MCR with caring responsibilities.

Model		Unstandardized Coefficients		Standardized Coefficients		
		B	Std. Error	Beta	t	Sig.
1	(Constant)	.209	.040		5.174	<.001
	dummy variable for mid-stage researcher	.445	.092	.384	4.812	<.001

Dependent Variable: do you have caring responsibilities?

Table S8k: Correlation of peer influence with family support.

Model		Unstandardized Coefficients		Standardized Coefficients		
		B	Std. Error	Beta	t	Sig.
1	(Constant)	.314	.043		7.301	<.001
	peer influence	-.203	.095	-.184	-2.135	.035

Dependent Variable: family support

Table S9: Cross-tabulation of gender and career stage.

	men		women		other		
Career stage	count	%	count	%	count	%	**Total count (%)**
Undergraduate	6	35.3%	11	64.7%	-	-	**17(12.5%)**
Postgraduate	25	41.0%	35	57.4%	1	1.6%	**61(44.9%)**
Early career	7	63.6%	3	27.3%	1	9.1%	**11(8.1%)**
Mid career	7	26.9%	19	73.1%	-	-	**26(19.1%)**
Late career	8	66.7%	4	33.3%	-	-	**12(8.8%)**
other	3	42.9%	3	42.9%	1	14.3%	**7(5.1%)**
Missing data							**2**
**Total count (%)**	**56(41%)**		**76(56%)**		**4(3%)**		**136(100%)**

Section S9 Supplementary References

84. C. S. Collins, C. M. Stockton, *Int. J. Qual. Met.* 2018, **17**, 1609406918797475.

85. H. Coombs, First-in-Family Students, www.hepi.ac.uk, 2022, (accessed 08/01/2025).

86. T. T. Ishitani, *J. High. Ed.,* 2006, **77**, 861-885.

87. R. D. Padgett, M. P. Johnson, E. T. Pascarella, *J. Coll. Stud. Dev.,* 2012, **53**, 243–266.

88. L. Horn, A-M. Nuñez, *Statistical Analysis Report Postsecondary Education Descriptive Analysis Reports Mapping the Road to College: First-Generation Students' Math Track, Planning Strategies, and Context of Support,* NCES, 2000.

89. P. T. Terenzini, L. Springer, P. M. Yaeger, E. T. Pascarella, A. Nora, *Res. High. Educ.,* 1996, **37**, 1–22.

90. J. A. Redford, K. Mulvaney Hoyer, J. Ralph, *First-Generation and Continuing Generation College Students: A Comparison of High School and Postsecondary Experiences Stats in Brief*, NCES, 2017.

91. C. P. Zalaquett, *Psychol. Rep.,* 1999, ***85***(2), 417-421.

92. M. M. Jæger, *Am. Sociol. Rev.,* 2012, **77**, 903–922.

93. First Generation Students in Higher Education, Postsecondary National Policy Institute, PNPI, 2023, https://pnpi.org/factsheets/first-generation-students/ (accessed 08/01/2025).

94. V. B. Saenz, S. Hurtado, D. Barrera, D. Wolf, F. Yeung, *First in my family: A profile of first-generation college students at four year institutions since 1971,* The Foundation for Independent Higher Education, 2007.

95. M. Estrada, M. Burnett, A. G. Campbell, P. B. Campbell, W. F. Denetclaw, C. G. Gutiérrez, S. Hurtado, G. H. John, J. Matsui, R. McGee, C. M. Okpodu, T. J. Robinson, M. F. Summers, M. Werner-Washburne, M. Zavala, *CBE Life Sci. Educ.*, 2016, **15**, Fall;15(3):es5.

96. S. Nichols, G. Stahl, *High. Educ. Res. Dev.,* 2019, **38**, 1255–1268.

97. D. R. Means, K. B. Pyne, *J. Coll. Stud. Dev.,* 2017, **58**, 907–924.

98. Y. K. Kim, L. J. Sax, *Research in Higher Education: Journal of the Association for Institutional Research,* 2009, **50**, 437–459.

99. R. Covarrubias, I. Valle, G. Laiduc, M. Azmitia, *J. Adolesc. Res.,* 2019, **34**, 381–410.

100. J. Engle, V. Tinto. *Moving Beyond Access.* www.pellinstitute.org, 2008, (accessed 08/01/2025).

101. K. N. Upah, *Comparing First-Generation and Continuing-Generation College Students' Self Efficacy, Campus Involvement, and Academic Students' Self-Efficacy, Campus Involvement, and Academic Performance* https://scholarworks.uni.edu/hpt, 2017, (accessed 08/01/2025).

102. B. N. Richards, *Soc. Probl.,* 2022*,* **69**, 241–260.

103. P. Bourdieu, Outline of a Theory of Practice, *Cambridge University Press*, 1977.

104. K. Thomas, *WPLL*, 2015, **17**, 37-49.

105. D. B. Bills, D. B. Credentials, *Rev. Educ. Res.,* 2003, **73**, 441–469.

106. S. R. Jenkins, A. Belanger, M. L. Connally, A. Boals, K.M. Durõn, *J. Coll. Couns.,* 2013, **16**, 129–142.

107. Royal Society of Chemistry, A sense of belonging in the chemical sciences, *Royal Society of Chemistry,* 2021.

108. C. Halse in Interrogating Belonging for Young People in Schools. (C. Halse, Ed.) *Springer International Publishing,* 2018.

109. V. May, *Soc.*, 2021, **45,** 363-378.

110. J. Leigh, J. Sarju, A. Slater in Belonging in STEM. (C. Kandiko-Smith, M. Kingsbury, Eds.) *UCL Press*, 2024.

111. T. H. Zunder, *Eur. Transp. Res. Rev.*, 2021, **13**, 1–14.

112. Google Scholar. https://scholar.google.com/ (accessed 08/01/2025).

113. First Gen Forward. https://firstgen.naspa.org/about-firstgen-forward/about-firstgen-forward , (accessed 08/01/2025).

114. Women in Supramolecular Chemistry. https://www.womeninsuprachem.com, (accessed 08/01/2025).

115. T. Kim, W. Ng, *Policy Rev. High. Educ.,* 2019, **3**, 94–119.

116. V. Boyd, *Intl. J. Disabil. Dev. Educ.,* 2014, 61(**4**), 377–387.

117. K. D. Parry, B. G. Clarkson, E. J. Kavanagh, R. Sawiuk, L. Grubb, *Int. Rev. Sociol. Sport*, 2023, **58**, 867–888.

118. Diversity and inclusion survey (DAISY) question guidance. https://edisgroup.org/resources/practical-tools-and-guidance/diversity-and-inclusion-survey-daisy-question-guidance-v2/, *Wellcome Trust*, 2022 (accessed 08/01/2025).

119. V. Braun, V. Clarke, *Qual. Res. Psychol.* 2021, **18**, 328–352.

120. D. F. Polit, C. T. Beck, *Int. J. Nurs. Stud.*, 2010, **47**, 1451–1458.
